# Population Survival Kinetics Derived from Clinical Trials of Potentially Curable Lung Cancers

**DOI:** 10.3390/curroncol31030122

**Published:** 2024-03-20

**Authors:** David J. Stewart, Katherine Cole, Dominick Bosse, Stephanie Brule, Dean Fergusson, Tim Ramsay

**Affiliations:** 1 Ottawa Hospital Research Institute, University of Ottawa, Ottawa, ON K1H 8L6, Canada; katcole@toh.ca (K.C.); sbrule@toh.ca (S.B.); dafergusson@ohri.ca (D.F.);; 2 Department of Epidemiology and Biostatistics, University of California, San Francisco, CA 94143, USA

**Keywords:** lung cancer, progression-free survival, population survival kinetics, tutorial on population survival kinetics

## Abstract

Using digitized data from progression-free survival (PFS) and overall survival Kaplan–Meier curves, one can assess population survival kinetics through exponential decay nonlinear regression analyses. To demonstrate their utility, we analyzed PFS curves from published curative-intent trials of non-small cell lung cancer (NSCLC) adjuvant chemotherapy, adjuvant osimertinib in resected *EGFR*-mutant NSCLC (ADAURA trial), chemoradiotherapy for inoperable NSCLC, and limited small cell lung cancer (SCLC). These analyses permit assessment of log–linear curve shape and estimation of the proportion of patients cured, PFS half-lives for subpopulations destined to eventually relapse, and probability of eventual relapse in patients remaining progression-free at different time points. The proportion of patients potentially cured was 41% for adjuvant controls, 58% with adjuvant chemotherapy, 17% for ADAURA controls, not assessable with adjuvant osimertinib, 15% with chemoradiotherapy, and 12% for SCLC. Median PFS half-life for relapsing subpopulations was 11.9 months for adjuvant controls, 17.4 months with adjuvant chemotherapy, 24.4 months for ADAURA controls, not assessable with osimertinib, 9.3 months with chemoradiotherapy, and 10.7 months for SCLC. For those remaining relapse-free at 2 and 5 years, the cure probability was 74%/96% for adjuvant controls, 77%/93% with adjuvant chemotherapy, 51%/94% with chemoradiation, and 39%/87% with limited SCLC. Relatively easy population kinetic analyses add useful information.

## 1. Introduction

Progression-free survival (PFS) and overall survival (OS) Kaplan–Meier plots have several potential uses in the analysis and reporting of clinical trials, including estimations of medians and calculation of hazard ratios. We have also used clinical trial data from digitized PFS and OS Kaplan–Meier curves for exponential decay nonlinear regression analyses to assess population survival kinetics.

Population survival kinetics model the disappearance rate of patients from a population, analogous to pharmacokinetics modeling the rate of drug disappearance from the blood [1]. These analyses permit the calculation of PFS and OS half-lives (time to progression or death of half the remaining patients) and assessment of log–linear curve shape. For PFS and OS, the half-lives and medians correlate strongly (R^2^ ≥ 0.96) with each other [2].

Importantly, the PFS log–linear curve shape varies significantly with therapy type [3,4]. These curves generally follow first-order kinetics, approximating a straight line on log–linear plots, unless factors that impact therapy efficacy cause deviations from straight lines. Consequently, log–linear curve shapes provide insight into factors impacting therapy efficacy. For example, many combination chemotherapy PFS curves demonstrate convexity starting at 3–4 months since progression accelerates following therapy interruption upon the completion of induction [4].

Distinct subpopulations with differing progression rates can result in a rightward inflection on PFS log–linear curves, with curve data fitting two-phase decay nonlinear regression models. The probability of PFS curve two-phase decay is low for some therapies but high for others (e.g., EGFR inhibitors in unselected patients, where *EGFR* wild-type patients progress rapidly, while *EGFR*-mutant patients progress slowly) [4]. PFS 2-phase decay should prompt a search for a present-vs-absent factor predicting high-vs-low therapy benefit.

Population survival kinetics models also make PFS gain a good predictor of OS gain [2]. Drug approval can be delayed when using OS endpoints since the assessment of OS gain is compromised by crossover and because more patients and longer follow-ups are needed for adequate statistical power with OS endpoints [5]. Consequently, PFS endpoints are increasingly used for drug approval [6]. While PFS hazard ratios are weak predictors of OS hazard ratios [2,7,8,9], PFS half-life gain reliably predicts OS half-life gain. In 124 solid tumor trials with low crossover rates, a statistically significant PFS half-life gain ≥1.5 months predicted an OS half-life gain ≥2 months, with positive and negative predictive values of ≥80% [2]. Higher PFS gains reliably predicted even higher OS gains [2].

However, PFS gain underpredicted OS gain for immune checkpoint inhibitors [2]. PFS gain was also unreliable for prostate cancer (since prostate cancer metastasizes predominantly to bone, and bone metastases are difficult to measure) or for toxic high-dose therapies (where residual toxicity from first-line therapy can preclude effective second-line therapy). If prostate cancer (11 trials) and high-dose therapies (two trials) were excluded, then a PFS gain of ≥1.5 months predicted an OS gain of ≥2 months with positive and negative predictive values of 90% and 86%, respectively (DJ Stewart, unpublished data).

Population kinetic analyses can also guide the optimal frequency of follow-up scans [10] and reveal that, on average, 4% of the remaining patients with metastatic non-small cell lung cancer (NSCLC) die each week that therapy initiation is delayed [11].

In this paper, we present population survival kinetics analyses of PFS from clinical trials of lung cancer patients treated with curative intent. We assessed the use of this approach to estimate the proportion of the population that is cured, the PFS half-life for patients destined to eventually relapse, and the probability of future relapse for patients remaining relapse-free at different time points. In an online appendix, we provide a tutorial for clinicians, trainees, and others on how to use readily available tools for these analyses.

## 2. Materials and Methods

We identified relevant papers in PubMed. (See below.) For a publication to be included, it had to present a Kaplan–Meier PFS curve, relapse-free survival curve, or disease-free survival curve. Below, we use “PFS” to also refer to relapse-free and disease-free survival. As previously described [2,4,10,11], we used the application https://apps.automeris.io/wpd/ (accessed on 19 January 2024) to digitize the PFS curves from these publications.

The use of Kaplan–Meier curves as the source for our data adjusted for data censoring. We excluded curves derived from <50 patients to reduce potential variability arising solely from low patient numbers. Survival curves based on small sample sizes are subject to greater error, and the exclusion of these small trials could potentially improve the comparability of the remaining studies. The decision to use this particular cut point was arbitrary and could potentially be a source of bias. We have not tested the impact of using a higher or lower cut point.

We employed GraphPad Prism7 (GraphPad Software, La Jolla, CA, USA) built-in options for 1-phase and 2-phase exponential decay nonlinear regression analysis of digitized data and to replot data on log–linear scales. In these analyses, we applied the constraints “Y_0_ = 100” and “plateau = 0” since all curves start at 100% PFS and since all would eventually reach 0% survival if followed long enough.

The models provide an R^2^ value as an indicator of goodness of fit of the model for a particular curve. They also provide 95% confidence intervals and standard errors for the model parameters. However, the method has no specific control for experimental error that might be intrinsic within data for a given curve. To reduce the potential impact of experimental error associated with an individual curve, we calculated medians and ranges for model parameters across curves for a given treatment setting.

As in our earlier publications on population survival kinetics [2,4,10,11], we defined a curve as fitting a 2-phase decay model if the model identified two subgroups, each comprising ≥1% of the total population, with the PFS half-life for the group with longer PFS being more than twice as long as the half-life for the group with shorter PFS. Curves fitting 2-phase decay models typically demonstrate rightward inflection points on log–linear plots.

As previously described [10], we calculated the proportion of patients that we projected would remain progression-free at specific future time points using the Excel spreadsheet formula “=EXP(− t_n_ × 0.693/t_1/2_)” where “t_n_” is the time interval of interest from therapy initiation, “*” signifies multiplication, “0.693” is the natural log of 2, and “t_1/2_” is the subpopulation’s PFS half-life. 

A different way of expressing this formula would be “x = 2^(− t_n_/t_1/2_)” [10].

In this analysis, we assessed PFS curves for postoperative platinum-based adjuvant chemotherapy or control groups not receiving adjuvant chemotherapy for the 4 clinical trials included in the NSCLC LACE meta-analysis (Supplementary Online Table S1) [12,13,14,15,16], adjuvant osimertinib and the control group not receiving adjuvant osimertinib in resected *epidermal growth factor receptor*-mutant NSCLC (the ADAURA trial) [17], curative-intent chemoradiotherapy trials published from 2010 to February 2022 for inoperable NSCLC (57 curves from 37 clinical trials) (Supplementary Online Table S2) [18,19,20,21,22,23,24,25,26,27,28,29,30,31,32,33,34,35,36,37,38,39,40,41,42,43,44,45,46,47,48,49,50,51,52], and limited small cell lung cancer (SCLC) trials published 1990–2021 (55 curves) (Supplementary Online Table S3) [53,54,55,56,57,58,59,60,61,62,63,64,65,66,67,68,69,70,71,72,73,74,75,76,77,78,79,80,81,82,83,84,85,86,87]. 

Publications for chemoradiotherapy were identified in PubMed searches with the filter “Clinical Trial” that included the terms “radiotherapy or radiation or irradiation”, “non-small cell lung OR adenocarcinoma of the lung OR squamous cell carcinoma of the lung”, “locally advanced OR stage III OR stage 3”, “NOT metastatic”, “NOT stereotactic”, and “NOT neoadjuvant”. We identified 379 trials and excluded 143 that had fewer than 50 patients per trial arm, 2 with mixed populations of small cell and non-small cell lung cancer, 11 with no published PFS curves, 4 that did not include both chemotherapy and radiotherapy, and 132 that did not have PFS, relapse-free survival or disease-free survival as an endpoint. From the eligible trials, we also excluded 6 curves with the longest follow-up of less than 25 months. 

Publications relating to limited small cell lung cancer were identified in PubMed searches with the filter “Clinical Trial” that included the terms “small cell lung”, “NOT non-small cell lung”, and “limited”. We identified 754 trials and excluded 218 with fewer than 50 patients per trial arm, 10 with no published PFS curves, 55 that included previously treated patients, 125 that included mixed populations of limited and extensive small cell lung cancer or small cell lung cancer plus non-small cell lung cancer, and 236 that did not have PFS as an endpoint.

For comparisons between the groups, we used GraphPad Prism7 to perform nonparametric statistical analyses.

## 3. Results

### 3.1. Overall PFS Half-Lives

We used one-phase decay models to estimate the overall trial arm PFS half-lives (Table 1). The median value for overall PFS half-lives was 50.0 months for control arms on adjuvant chemotherapy trials, 61.1 months for adjuvant chemotherapy arms, 25.0 months for the ADAURA control arm, 109.1 months for adjuvant osimertinib, 13.9 months for NSCLC chemoradiation, and 16.2 months for limited SCLC. Note that for this outcome and for outcomes below, we combined data from trials that included patients with different characteristics and treatment details. These differences across trials will have impacted the outcomes and contributed to the heterogeneity seen across trials. Future trials using individual patient data and/or more homogeneous populations would improve the reliability of the conclusions.

### 3.2. 2-Phase Decay Modeling

All curves except seven SCLC curves [54,58,61,66,73,79], one chemoradiation curve [88] and the ADAURA osimertinib curve [17] fit two-phase decay models, in keeping with distinct potentially cured (“slow t_1/2_”) vs. relapsing (“fast t_1/2_”) subpopulations (for the ADAURA control population, the curve’s terminal vertical drop was excluded for two-phase decay assessment).

Curves were less likely to fit two-phase decay models if the curve length (maximum follow-up) was short. Only two of the seven SCLC curves that failed to fit two-phase models had a maximum follow-up of >42 months compared to 45 of 48 curves fitting two-phase models (*p* = 0.0003). Similarly, the chemoradiotherapy curve that did not fit a two-phase model had a maximum follow-up of 36 months, while 52 of 56 curves fitting two-phase models had follow-ups of >36 months. All curves would probably fit two-phase decay models with longer follow-ups since all would have cured vs. relapsing subpopulations.

### 3.3. Models “Hitting Constraints”

GraphPad specified that the nonlinear regression models “hit constraints” if calculations were unreliable near the constraint values. For models “hitting constraints”, upper and/or lower boundaries of 95% confidence intervals may not be calculated for the relevant model parameter(s).

Of the 113 PFS curves meeting our definition of fitting two-phase models, 62 “hit constraints”. In exploratory analyses, removing the constraint “Y = 100” generally did not alter “hit constraint” notifications. Removing the constraint “plateau = 0” generally did eliminate “hit constraint” notifications. However, when this constraint was removed, data generally no longer fit two-phase models, despite there being compelling evidence of two-phase decay (rightward inflection on the log–linear curves and the presence of distinct cured vs. relapsing subpopulations). Hence, we elected to maintain this constraint, recognizing that confidence intervals might not be provided for some parameters.

Shorter PFS curves increased the probability of hitting the constraints (Table 2).

### 3.4. Proportion of Patients in Potentially Cured Subpopulations

Via two-phase decay analyses, the median value across trial arms for the proportion of the population in the potentially cured subpopulation was 41% for the adjuvant control groups, 58% with adjuvant chemotherapy, 17% for the ADAURA controls, not yet assessable for the ADAURA osimertinib arm, 15% for chemoradiotherapy, and 12% for SCLC (Table 1). Longer follow-up will be needed before we can assess this for the ADAURA osimertinib arm.

Across all series, the proportion of patients in the potentially cured subpopulation was 12% for curves hitting constraints vs. 19% for other curves (*p* = 0.006). The upper 95% confidence interval boundaries could not be calculated for percent cured for 3 of the 62 curves hitting constraints vs. 3 of 48 curves not hitting the constraints. The maximum PFS curve length was a median of 65 months when 95% confidence interval upper boundaries could be calculated and 50 months where they could not be calculated (*p* = 0.0003).

### 3.5. PFS Half-Lives for Relapsing Subpopulations

The median PFS half-life for the relapsing subpopulation was 11.9 months for the adjuvant trial control patients, 17.4 months with adjuvant chemotherapy, not definable for the ADAURA osimertinib arm, 24.4 months for the ADAURA control patients, 9.3 months with chemoradiotherapy, and 10.7 months for SCLC (Table 1). Across the series, the PFS half-life for the relapsing subpopulations was 10.3 vs. 9.9 months for curves hitting vs. not hitting constraints (*p* = 0.09).

For the PFS half-life for relapsing subpopulations, 95% confidence interval upper boundaries could not be calculated for 45 of 62 curves hitting constraints vs. 13 of 49 curves not hitting constraints. The maximum curve length was a median of 72 months where 95% confidence interval upper boundaries could be calculated and 60 months where they could not be (*p* = 0.06).

### 3.6. PFS Half-Lives for Potentially Cured Subpopulations

The median PFS half-life of the potentially cured subpopulation was 396.2 months with adjuvant chemotherapy, 1.1 × 10^12^ months for adjuvant controls, 3.3 × 10^15^ months for chemoradiotherapy, and 3.7 × 10^15^ months for SCLC (Table 1).

The upper boundaries of the 95% confidence intervals for long half-lives could not be calculated for any of the 62 curves hitting constraints, and lower boundaries were non-calculable for 61 of these 62 curves. For curves not hitting the constraints, the upper boundaries could not be calculated for 31 of 49 curves, but lower boundaries could be calculated for all 49. Very wide or indeterminate confidence intervals would be expected for this parameter since the duration of patient follow-up was much shorter than the cured subpopulation’s projected PFS half-life. The maximum curve length was a median of 98 months where 95% confidence interval upper boundaries could be calculated and 60 months where they could not be (*p* < 0.0001).

In many cases, PFS half-lives for potentially cured subpopulations were much longer than human life expectancy. Hence, two-phase decay models frequently overestimated true PFS half-lives for potentially cured subpopulations. True PFS half-lives were probably long, but we cannot reliably estimate how long. We need a much longer follow-up to narrow the 95% confidence intervals. For curves for which both upper and lower boundaries of 95% confidence intervals could be calculated, the median PFS half-life value for potentially cured subpopulations across trials was 112.7 months (9.4 years).

The observation that the PFS half-life for the potentially cured subpopulation was shorter with adjuvant chemotherapy than for controls might be due to the imprecision of the analytical method. Alternatively, we might hypothesize that chemotherapy substantially delays relapse in some patients (e.g., by inducing reversible senescence [89]) without killing all of the remaining tumor cells. If so, we might eventually expect three distinct subgroups on three-phase decay exponential decay nonlinear regression analysis: a rapidly relapsing subgroup, a slowly relapsing subgroup, and a cured subgroup. Three of four adjuvant chemotherapy PFS curves could be fit using three-phase decay models, but the 95% confidence intervals were very wide. The assessment of individual patient data might provide added insight.

### 3.7. Early PFS Curve Convexity on Log–Linear Plots

**Figure 1:**
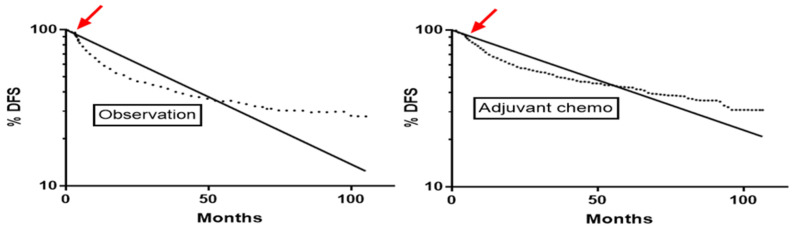
Onset of convexity (arrow) on disease-free survival curves at 3.3 months in the observation arm and at 4.8 months in the adjuvant chemotherapy arm of the ANITA trial [12]. Curves after this early convexity follow 2-phase decay. The dotted line is the Kaplan-Meier curve and the solid line is the log linear one-phase decay nonlinear regression line.

All except one curve had initial convexity, with the curve steepening after an initial brief plateau or shallow decline (Figure 1). For adjuvant osimertinib, this convexity started at 33 months, in keeping with therapy being discontinued at 36 months (Table 1). The convexity began at a median of 3.3 months for control arms in adjuvant chemotherapy and ADAURA trials, in keeping with the convexity probably being an artefact due to lack of detection of progression until the first follow-up scan. Convexity onset was at a median of 6.1 months for adjuvant chemotherapy trial arms, 4.6 months with chemoradiotherapy, and 6.2 months with SCLC, in keeping with therapy delaying tumor progression in some patients who are not cured by therapy. Across the series, the time of convexity onset was similar for curves hitting vs. not hitting constraints (4.6 vs. 4.4 months, *p* = 0.29).

For 59 of the 115 PFS curves with a late convexity point, there was also an earlier convexity point at a median (range) of 2.9 (1.0–7.1) months. We hypothesized that the early convexity point was generally based on an artefactual delay in identifying early progression due to the timing of the first follow-up scans, while the later convexity point, with further steepening of log–linear curves, was due to tumor growth acceleration in non-cured patients following therapy discontinuation.

### 3.8. Proportion of Remaining Patients Who Would Continue to Be Progression-Free at Different Time Points

The Excel formula “=Exp(− t_n_ × 0.693/t_1/2_)” permitted estimation of the proportion of patients in relapsing subpopulations who would remain progression-free at different time points (Table 3). With adjuvant chemotherapy (both treated and control groups), chemoradiotherapy and SCLC, our analyses suggest that 11–20% of the rapidly progressing subpopulations would already have progressed by 3 months post therapy initiation.

Due to early convexities on the PFS curves, these calculations might overestimate or underestimate the proportion progressing by 3 months, but these curve convexities would have less impact on calculations for later time points since half-life estimates included the deviation caused by early convexities. The estimates indicate that frequent follow-up scans would initially be important to detect early relapses.

For the calculations of the percent of patients destined to eventually progress shown in Table 3, we coded the size of the potentially cured subpopulations as being constant for the adjuvant trial controls and chemoradiotherapy and SCLC groups. This assumption was based on the long PFS half-lives for these subpopulations. This would somewhat overestimate the size of this subpopulation at later times since at least some patients would have died from other causes, even if none relapsed.

For those receiving adjuvant chemotherapy, we coded the size of the potentially cured subpopulation as decreasing slowly over the time of interest, based on the subpopulations’ half-lives. It is unknown if the gradual decrease in size of this adjuvant chemotherapy subpopulation was due to eventual tumor progression vs. deaths from other causes. In addition, these calculations might change for very late time points if there were a second rightward inflection point on the PFS log–linear plots, as discussed above.

Acknowledging the limitations previously noted, the data presented in Table 3 can be used to estimate the risk of future progression for patients who remain progression-free at different time points. Across these different patient groups, the probability of cure would be 39–77% for those remaining relapse-free at 2 years, 75–91% for patients remaining relapse-free at 4 years and 87–96% for those remaining relapse-free at 5 years. There would continue to be a small possibility of relapse (but <1%) at 10 years and beyond.

### 3.9. Optimum Frequency of Follow-Up Scans

For all groups, the relapsing subpopulation would become a lower proportion of the entire remaining population over time, and it would be reasonable to perform scans progressively less frequently over time (Table 4). For example, in the “adjuvant controls” group in Table 4, 17% of the patients would be found to have progressed on a scan undertaken 6 months after surgery, but for patients who remained progression-free 24 months after surgery, only 8% would subsequently be found to have progressed on a scan performed 6 months later at 30 months after surgery. Hence, across these different curative therapy situations, relatively frequent scans might be considered early following therapy initiation, with no further follow-up scans at later time points except for investigation of concerning new symptoms or for screening for new primaries.

### 3.10. Changes in SCLC Outcomes over Time

We also performed additional exploratory assessments. In SCLC, the outcome was better for studies published 2011–2021 than for studies published 1990–2010, with the median value for PFS half-lives increasing to 19.5 months from 15.0 months (*p* = 0.003), with the proportion in the potentially cured subpopulation increasing to 22% from 12% (*p* = 0.006), and with 92% vs. 87% potentially cured among those progression-free at 5 years. This apparent improvement might be due to stage migration (with increasing staging with PET/CT scanning) or to improved radiotherapy approaches.

### 3.11. Durvalumab Addition to Chemoradiation

For the PACIFIC trial comparing durvalumab to placebo following chemoradiotherapy [25], the durvalumab arm had an overall PFS half-life of 23.6 months, and the proportion of the patients in the potentially cured arm was high, at 57%. This favorable outcome was due in part to patient selection since patients could only receive durvalumab if they completed chemoradiotherapy without progressing. In keeping with this, the proportion of potentially cured patients in the control group was also relatively high, at 26%.

The PFS half-life for the potentially cured durvalumab subpopulation was shorter than for many other trials, at 61.4 months. Overall, durvalumab substantially prolonged PFS, but further follow-up will be required to determine if it is increasing the probability of true cure. If it is only delaying progression, then PFS curves with longer follow-up may eventually demonstrate three-phase decay, with a relapsing subpopulation, a cured subpopulation, and an intermediate group with delayed progression, as discussed earlier for adjuvant chemotherapy.

PFS half-lives in the relapsing subpopulation were similar on the durvalumab and placebo arms. In our earlier assessments of PFS curves for different therapy types, immune checkpoint inhibitors appeared to either have a marked beneficial effect or else almost no benefit in patients with metastatic disease [4]. The same may be happening in this post chemoradiation setting.

### 3.12. Chemotherapy Regimen Added to Curative Radiotherapy

Acknowledging the limitations of cross-study comparisons, we found no evidence of benefit from induction chemotherapy prior to concurrent chemoradiotherapy or of consolidation chemotherapy following chemoradiotherapy (Table 5), in keeping with published meta-analyses [90]. We also saw no evidence of a meaningful difference in outcome between pemetrexed, a topoisomerase inhibitor or a vinca alkaloid when used concurrently with radiation.

However, we noted some variability in the efficacy of different chemotherapy regimens, in keeping with prior publications [91]. Specifically, the use of taxane plus platinum concurrently with radiation was associated with a shorter overall PFS half-life than the use of another agent with platinum (11.5 months vs. 13.7 months, *p* = 0.03) and with a slight reduction in the percent of patients in the potentially cured subpopulation (10.5% vs. 13%, *p* = 0.049). The overall PFS half-life was 11.4 months for a carboplatin regimen concurrently with radiation vs. 13.7 months with a cisplatin regimen (*p* = 0.04), and the proportion of patients in the potentially cured subpopulation was 11% vs. 12.5% (*p* = 0.14).

Ten of the thirteen trials using carboplatin also used taxane, and ten of the fourteen trials using taxane also used carboplatin. Hence, if drug choice has a true impact, it is unclear if the major impact is taxanes vs. other agents or if it is carboplatin vs. cisplatin. Nevertheless, while some differences were statistically significant, they were small and of limited clinical significance.

## 4. Discussion

We have previously published work on the use of population survival kinetics analyses in relation to incurable solid tumors [2,3,4,10,11], with limited prior data presented on their use in potentially cured populations [3]. Here, we expand on the use of the methodology in potentially cured populations. These analyses permit estimation of the proportion of patients potentially cured, the PFS half-life for the subpopulation destined to eventually relapse, and the probability of eventual progression in patients remaining progression-free at different time points after therapy initiation.

Similar assessments could also potentially be undertaken using other available methods, but this method is convenient since it can be easily and rapidly performed using readily available tools. Within minutes, published PFS and OS curves can be digitized, converted to log–linear plots, and analyzed. Here, and in earlier publications [2,3,4,10,11], we have discussed how population survival kinetics analyses might offer insights from clinical trial data over and above the insights typically gained from the calculation of medians, hazard ratios, *p* values, etc.

The premise of this approach is that PFS and OS curves generally follow first-order kinetics, with log–linear plots that approximate straight lines, and that major deviations from a straight log–linear line may offer useful insights. For example, early curve convexity at 1–3 months is seen since early progression is generally not identified until a patient’s first follow-up scan. Later curve convexity, typically starting at 3.5 months or longer after therapy initiation, can occur due to the late acceleration of progression or death. This might arise from drug dose reduction or discontinuation prior to tumor progression, from late initiation of an additional agent or measure that reduced drug absorption or escalated drug elimination, or from the late acceleration of the development of a resistance mechanism. The assessment of individual patient data could elucidate potential causes. Rightward log–linear curve deviation at an inflection point suggests the presence of two distinct subpopulations based on a dichotomous present vs. absent factor that impacts prognosis or therapy efficacy.

Population survival kinetics analyses can be used to assess if two subpopulations or trial arms differ with respect to the overall slope of their PFS or OS curves, the extent of late acceleration of progression or death, the relative size of a favorable subpopulation, and the rapidity of progression of the rapidly progressing subpopulation. One can estimate the proportion of the population that is potentially cured, the probability of future relapse at any time point, and the proportion of remaining patients who will remain progression-free or alive at different time points in the future. These analyses would permit the easy incorporation of these parameters into standard reporting of clinical trial outcomes.

The shape of the PFS and OS curves can be interpreted in different ways. For example, if two curves cross or initially overlap and then separate, one common explanation is that “therapy benefit occurs late”. Conversely, the population survival kinetics explanation is that the better curve has two distinct subpopulations: one subpopulation derives little or no added benefit from the new therapy and has a PFS similar to that of the control group. Conversely, the second subpopulation derives substantial benefit from the new therapy and is doing much better than the control group.

When curves initially separate and then come together, one potential explanation is that a treatment is losing its effect over time. Population survival kinetics assessments suggest that it instead often means that for a therapy that was controlling a tumor, therapy interruption is followed by rapid progression.

Since we first undertook population survival kinetics assessments, our approaches and perspectives have evolved, and we anticipate that they will continue to change as we and others further test this approach.

With respect to future applications, population survival kinetics methods could be applied to a wide range of both oncology data and non-oncology biological states. Artificial intelligence approaches could be explored to rapidly retrieve and analyze very large amounts of clinical and research data. As noted above, using individual patient data could facilitate the use of approaches such as nonlinear mixed effects modeling to assess the impact of relevant clinical variables on outcomes.

## 5. Limitations

Our proposed population survival kinetics methods have some potential limitations. As noted previously, early PFS curve convexities could result in overestimation or underestimation of the proportion of patients progressing at 3 months but would be expected to have less of an impact on estimates of progression at later time points. In addition, based on the long PFS half-lives for the adjuvant trial controls and chemoradiotherapy and SCLC groups, we coded the size of the potentially cured subpopulations as being constant. This somewhat overestimates the size of this subpopulation at later times since, even in the absence of relapse, at least some patients would die from other causes.

As noted previously, the method has no specific control for experimental error that might be intrinsic within data for a given curve. However, in the online Supplementary Tables S1–S3, we included the sample size for each trial to help the reader assess the strength of the evidence.

We also recognize that conclusions from across-study comparisons (e.g., with respect to the induction or consolidation chemotherapy with radiotherapy) have to be interpreted cautiously.

Furthermore, we have not yet tested this methodology with individual patient data and real-world evidence. Using individual patient data could facilitate the assessment of the role of dose reductions, therapy interruptions and other factors on curve convexity. It could also facilitate the assessment of the impact on outcomes of various patient and biological characteristics, such as tumor size, tumor differentiation, PD-L1 expression, tumor mutations, gender, age, race/ethnicity, etc. Methods such as nonlinear mixed effects modeling [92] might prove useful in this. This could facilitate increased personalization of optimal scan frequency, prediction of the probability of future relapse, etc.

Another limitation is that short patient follow-up is as limiting for these analyses as for any other approach used in assessing clinical trial data. In our two-phase decay analyses, 95% confidence intervals were generally narrow for the proportion of patients in the relapsing subpopulation, but they were typically very wide or not calculable for PFS half-lives for potentially cured subpopulations. As would be expected, more mature data with longer patient follow-ups were associated with a greater probability that 95% confidence intervals could be calculated.

Low patient numbers at the terminal portion of a survival curve mean that the loss of one patient might lead to a large drop in the curve. Conversely, the presence of a single long survivor might impact assessment by producing a very long tail on the curve. In some of our earlier analyses [2,4,10], we truncated curves if there were less than an estimated 10 remaining patients. We have not yet adequately assessed the impact of this truncation, but in preliminary assessments, it generally had minimal impact on the overall curve half-life but did somewhat reduce the probability of a curve fitting two-phase decay models (D. Stewart, unpublished data). Our requirement that each subpopulation had to be ≥1% of the entire population for a curve to be designated as fitting a two-phase decay model also means that the method would miss very small favorable subpopulations.

As with any methodology, there is at least some risk of incorrect conclusions due to misinterpretation of data. As much caution is required in the assessment of results using these methods as with any analytical method. One advantage of this approach is that these analyses can be reassessed easily by others since they use accessible published data and readily available analytical tools.

## 6. Conclusions

In summary, population survival kinetics analyses can be performed easily and rapidly using readily available tools. These analyses may provide useful insights into clinical trial data, including PFS log–linear curve shape, PFS half-life for the overall population, the relative size of distinct subpopulations, PFS half-lives for subpopulations, and the probability of future relapse for patients remaining relapse-free at a given time point.

## Figures and Tables

**Table 1:** Medians (ranges) for population survival kinetics PFS variables for potentially cured lung cancer.

Variable	Adjuvant NSCLC Controls Group ^a^	Adjuvant NSCLC Chemotherapy Group ^a^	ADAURA NSCLC *EGFR*-Mutant Control Group	ADAURA NSCLC *EGFR*- Mutant Adjuvant Osimertinib Group	Locally Advanced NSCLC Chemoradiation ^b^	Limited SCLC ^c^
No. curves	4	4	1	1	57	55
Overall PFS half-life (months)	50.0 (35.0–64.8)	61.1 (46.2–100.0)	25.0	109.1	13.9 (7.2–40.3)	16.2 (7.7–30.0)
Onset of late curve convexity (months)	3.3 (3.0–4.3)	6.1 (4.3–7.7)	2.9	30.0 ^g^	4.6 (1.5–13.1) ^h^	6.2 (3.1–19.0)
% short PFS ^d^	59 (50–94)	42 (40–60)	83	No fit for 2-phase decay model	85 (4–95) ^h^	88 (67–97) ^i^
Fast t_1/2_ (months) ^e^	11.9 (10.0–55.4)	17.4 (9.9–29.5)	24.4		9.3 (4.0–15.7) ^h^	10.7 (5.4–15.6) ^i^
Slow t_1/2_ (months) ^f^	1.1 × 10^14^ (207.9–4.1 × 10^15^)	396.2 (114.2–3.8 × 10^15^)	1.2 × 10^12^		3.3 × 10^15^ (21.8–6.1 × 10^15^) ^h^	3.7 × 10^15^ (59.5–6.1 × 10^15^) ^i^

^a^. Data for individual trials are presented in Supplementary online Table S1. ^b^. Data for individual trials are presented in Supplementary online Table S2. ^c^. Data for individual trials are presented in Supplementary online Table S3. ^d^. The proportion of the population destined to relapse (the subpopulation with short PFS). ^e^. PFS half-life for the subpopulation destined to relapse (the subpopulation with short PFS). ^f^. PFS half-life for the potentially cured subpopulation (the subpopulation with long PFS). ^g^. The ADAURA adjuvant osimertinib curve also had an earlier downward convexity starting at 10.5–14.5 months. ^h^. One curve did not demonstrate an early curve convexity, and one could not be fit by a 2-phase model. ^i^. Seven curves could not be fit using a 2-phase decay model.

**Table 2:** Two-phase decay models and hitting the constraint “plateau = 0”, across all series.

Characteristic	Hit Constraint	Did Not Hit Constraint	*p*
No. curves	62	49	
Maximum curve length (median, months)	64.8	85.6	0.03
Proportion of population progression-free at last follow-up (median, %)	17.4	15.4	0.29
Proportion in potentially cured subpopulation (median, %)	12	19	0.006
Upper boundary 95% CI could be calculated (% of curves) ^a^	95	94	0.99
Lower boundary 95% CI could be calculated (% of curves) ^a^	97	98	0.99
PFS half-life for the relapsing subpopulation (median, months)	10.3	9.9	0.09
Upper boundary 95% CI could be calculated (% of curves) ^a^	27	73	<0.0001
Lower boundary 95% CI could be calculated (% of curves) ^a^	97	100	0.50
PFS half-life for the potentially cured subpopulation (median, months)	4.3 × 10^15^	370.5	<0.0001
Upper boundary 95% CI could be calculated (% of curves) ^a^	0	37	<0.0001
Lower boundary 95% CI could be calculated (% of curves) ^a^	2	94	<0.0001
Onset PFS curve convexity (median, months)	4.6	4.4	0.29
2-phase decay model R^2^ (median)	0.96	0.98	<0.0001

^a^ 95% CI: 95% confidence interval.

**Table 3:** A proportion of patients remaining progression-free at different time points.

Time ^a^	% of Relapsing Subpopulation Who Remain Progression-Free ^e^				% of Long PFS Adjuvant ^c^ Patients Alive and Progression-Free ^f^	% of All Remaining Patients Destined to Eventually Relapse ^g^				% of Those Remaining Progression-Free Who May Be Cured ^i^			
	Adjuvant Controls ^b^	Adjuvant ^c^	NSCLC Chemoradiation ^d^	SCLC		Adjuvant Controls ^b^	Adjuvant ^c,h^	NSCLC Chemoradiation ^d^	SCLC	Adjuvant Controls ^b^	Adjuvant ^c,h^	NSCLC Chemoradiation ^d^	SCLC
0	100	100	100	100	100	59	≥42	86	88	41	≤58	14	12
2	89	92	86	88	99.7	56	≥40	83	87	44	≤60	17	13
3	84	89	80	82	99.5	55	≥39	82	86	45	≤61	18	14
4	79	85	74	77	99.3	53	≥38	81	85	47	≤62	19	15
6	71	79	64	68	99	50	≥37	78	83	50	≤63	22	17
8	63	73	55	60	98.6	47	≥35	74	81	53	≤65	24	19
9	59	70	51	56	98.4	46	≥34	73	80	54	≤66	26	20
12	50	62	41	46	98	42	≥31	70	77	58	≤69	30	23
18	35	49	26	31	97	34	≥27	60	70	66	≥73	40	30
24	25	38	17	21	96	26	≥23	49	61	74	≤77	51	39
36	12	24	7	10	94	15	≥16	28	42	85	≤84	72	58
48	6	15	3	4	92	8	≥10	14	25	92	≤90	86	75
60	3	9	1	2	90	4	≥7	6	13	96	≤93	94	87
120	0.1	0.1	0.01	0.04	81	0.1	≥0.7	0.1	0.3	99.9	≤99.3	99.9	99.7

^a^. Months from initiation of therapy. ^b^. Control group for NSCLC adjuvant chemotherapy trials. ^c^. NSCLC adjuvant chemotherapy group. ^d^. Locally advanced NSCLC chemoradiation. ^e^. Calculated using the Excel formula “=EXP(−t_n_ × 0.693/t_1/2_)”, where t_n_ is the time of interest from therapy initiation (3, 6, 9 months, etc.), 0.693 is the natural log of 2, and t_1/2_ is the median PFS half-life for the relapsing subpopulation (12.9 months for the adjuvant trial control group, 12.5 months for the adjuvant chemotherapy group, 9.3 months for NSCLC chemoradiation patients and 10.7 months for limited SCLC patients). ^f^. The proportion of long PFS subpopulation of adjuvant chemotherapy patients who would remain progression-free if there is no later inflection point on the PFS curve that reveals a third subpopulation with longer control. If there is a second inflection point, the proportion alive and progression-free at later time points would be somewhat higher than those presented. ^g^. The proportion of the entire remaining population that will eventually relapse. This is calculated by multiplying the proportion of the entire population that belonged to the relapsing subpopulation (59% in the adjuvant trials control group, 86% in the NSCLC chemoradiation group and 88% in the limited SCLC group) by the % of the relapsing subpopulation remaining progression-free at the time point of interest and dividing by the total remaining population. ^h^. The median PFS half-life for the “potentially cured” subpopulation is shorter for the adjuvant chemotherapy group than for other groups assessed. This could arise from calculation artefact, from late progression or from accelerated patient death (e.g., from chronic chemotherapy toxicity). The proportion of adjuvant chemotherapy patients destined to relapse or be cured would depend on whether drop-off in the long PFS subpopulation is due to progression vs. death from other causes and on whether there is eventually a second inflection point on the PFS curve. Because of this uncertainty, we added “≥” and “≤” modifiers for this group. ^i^. 100% minus percent of all remaining patients destined to eventually relapse.

**Table 4:** The percent of patients who remain progression-free at different time intervals (0–120 months) after therapy initiation who would be found to have progressed on a subsequent scan performed 2, 4, 6, or 12 months later.

Months between Scans	2	4	6	12	2	4	6	12	2	4	6	12	2	4	6	12	2	4	6	12
Interval start (months from therapy initiation)	For patients remaining progression-free at interval start, % who would progress by the next scan for scans performed after 2, 4, 6, or 12 months ^a^																			
	Adjuvant controls				Adjuvant chemotherapy				ADAURA control				Chemoradiation				SCLC ^b^			
0	6	12	17	30	3	6	9	16	5	9	13	24	12	22	31	50	11	20	28	48
2	6	12	16	28	3	6	8	15	5	9	13	24	12	22	30	49	10	20	28	47
4	6	11	15	27	3	6	8	15	5	9	13	24	11	21	29	48	10	20	27	46
6	6	11	15	25	3	5	8	14	5	9	13	23	11	20	28	46	10	19	27	45
8	5	10	14	24	3	5	7	13	5	9	13	23	11	20	27	45	10	19	26	44
12	5	9	12	21	3	5	7	12	5	9	12	23	10	18	25	41	9	18	25	42
18	4	7	10	17	2	4	6	10	4	8	12	22	8	16	21	35	8	16	22	38
24	3	6	8	13	2	3	5	9	4	8	11	21	7	13	18	29	7	14	19	33
36	2	3	4	8	1	2	3	6	4	7	10	18	4	7	10	16	5	10	13	22
48	1	2	2	4	1	2	2	4	3	6	9	16	2	4	5	8	3	6	8	13
60	<1	1	1	2	<1	1	1	3	3	5	8	14	1	2	2	4	2	3	4	7
120	<1	<1	<1	<1	<1	<1	<1	<1	1	2	2	4	<1	<1	<1	<1	<1	<1	<1	<1

^a^ The numbers progressing on the next scan decrease over time as patients progress and as the subpopulation destined to eventually relapse becomes a progressively smaller proportion of the entire remaining population. These calculations assume that patients in the “potentially cured” subpopulation will not be found to have progressed on follow-up scans and that the drop off in the “potentially cured” subpopulation for the adjuvant chemotherapy group is due to patients dying from other causes, and not from progression. ^b^ For SCLC, we used 12% for the proportion cured. In more recent studies, 22% of patients were potentially cured. Hence, the proportion progressing by the next scan would be somewhat lower than presented here.

**Table 5:** PFS for chemotherapy used concurrently with radiotherapy in locally advanced NSCLC.

Therapy ^a^	No. Studies	PFS Overall t_1/2_ (Months)			% Potentially Cured		
		Median	95% Cis ^e^		Median	95% Cis ^e^	
			Low	High		Low	High
Platinum used							
Cisplatin	28	13.7	13.0	16.7	12.5	12	18
Carboplatin ^b^	13	11.4	10.0	13.7	11	9	14
Agent combined with a platinum							
Taxane ^c^	14	11.5	10.1	13.4	10.5	9	14
+ Carboplatin	10	11.8	9.9	14.5	11.5	9	16
+ Cisplatin	4	10.9	8.7	12.8	9.5	5	13
Topoisomerase inhibitor ^d^	4	11.9	7.3	17.4	12.5	7	17
Pemetrexed	5	14.1	12.9	16.3	11	7	15
Vinca alkaloid	11	13.6	10.6	17.6	13	11	22
S-1	3	16.4	8.5	26.1	21	0	43
Induction or Consolidation chemotherapy before or after concurrent chemoradiotherapy							
Induction	14	14.9	12.8	18.3	13.5	11	22
Consolidation	8	13.2	10.4	17.3	12.5	9	18.5
Neither	27	13.7	12.2	16.2	13	13	25

^a^. For this analysis, we excluded trials that used a choice of different systemic therapy options in the same trial and trials for which the details of systemic therapy were not provided. ^b^. A taxane was the concurrent agent for 10 of the 13 evaluable carboplatin trials. ^c^. The concurrent platinum was carboplatin for 10 of 14 taxane trials and cisplatin for the other 4. ^d^. Etoposide for three trials and irinotecan for one. ^e^. 95% CIs: 95% confidence intervals.

## Data Availability

Data are available in Tables S1–S3.

## Supplementary Materials

The following supporting information can be downloaded at: https://www.mdpi.com/article/10.3390/curroncol31030122/s1, Table S1: PFS population survival kinetics assessment for NSCLC postoperative adjuvant platinum regimens vs controls, Table S2: PFS population survival kinetics assessment of chemoradiation for locally advanced NSCLC, Table S3: PFS population survival kinetics assessment of limited small cell lung cancer, Tutorial S1 on Population Survival Kinetics Methodology.
