# Supplementary material for: Population Survival Kinetics Derived from Clinical Trials of Potentially Curable Lung Cancers
Source: Curr Oncol. 2024 Mar 20;31(3):1600–17. doi: 10.3390/curroncol31030122 (PMC10968953; doi:10.3390/curroncol31030122)
Supplement: Supplementary file 1 [file curroncol-31-00122-s001.zip › curroncol-2869688-supplementary.pdf]

| Table S1. PFS population survival kinetics assessment for NSCLC postoperative adjuvant platinum regimens vs controls | | | | | | | | | | | | | | |
|---|---|---|---|---|---|---|---|---|---|---|---|---|---|---|
| Study arm | 1-phase decay models | | | | 2-phase decay models | | | | | | | | | |
| | Overall half-life[a] | 95% CI[a,b] | | $R^2$ | Relapsing subpopulation | | | | | | Potentially cured subpopulation | | | $R^2$ |
| | | | | | % of total | 95% CI[b] | | Half-life[a] | 95% CI[a,b] | | Half-life[a] | 95% CI[a,b] | | |
| | | Low | High | | | Low | High | | Low | High | | Low | High | |
| ANITA control [12] | 35.0 | 31.0 | 39.3 | 0.79 | 60 | 55 | 66 | 10.0 | 8.8 | 11.5 | 207.9 | 137.0 | 503.3 | 0.99 |
| ANITA chemo [12] | 47.2 | 44.5 | 50.0 | 0.82 | 41 | 38 | 44 | 9.9 | 8.9 | 11.0 | 114.2 | 103.1 | 129.6 | 0.99 |
| IALT control [13] | 40.1 | 37.8 | 42.4 | 0.82 | 57 | 54 | 60 | 12.9 | 12.2 | 13.8 | 212.7 | 170.9 | 290.0 | 0.99 |
| IALT chemo [13] | 46.2 | 44.1 | 48.5 | 0.99 | 42 | 40 | 45 | 11.7 | 10.9 | 12.5 | 120.8 | 111.2 | 133.4 | 0.99 |
| CALGB control [14] | 59.9 | 57.4 | 62.5 | 0.82 | 94 | ? | 96 | 55.4 | ? | ? | 1x10$^{14}$ | ? | ? | 0.91 |
| CALGB chemo [14] | 74.9 | 71.9 | 78.2 | 0.89 | 60 | 56 | 62 | 29.5 | 27.5 | 30.7 | 4x10$^{15}$ | ? | ? | 0.99 |
| BR10 control [15] | 64.8 | 58.3 | 72.1 | 0.27 | 50 | 49 | 51 | 10.9 | 10.4 | ? | 4x10$^{15}$ | ? | ? | 0.99 |
| BR10 chemo [15] | 100.0 | 95.3 | 105 | 0.84 | 40 | 31 | 48 | 23.0 | 18.0 | 28.0 | 671.6 | 314.2 | ? | 0.99 |
| a. months<br>b. 95% CI: 95% confidence intervals<br>?: could not be defined | | | | | | | | | | | | | | |

| Table S2: PFS population survival kinetics assessment of chemoradiation for locally advanced NSCLC | | | | | | | | | | | | | | | |
|---|---|---|---|---|---|---|---|---|---|---|---|---|---|---|---|
| Study | Arm[a] | 1-phase decay models | | | | 2-phase decay models | | | | | | | | | |
| | | | | | | Relapsing subpopulation | | | | | | Potentially cured subpopulation | | | $R^2$ |
| | | Overall half-life[b] | 95% CIs[b,c] | | $R^2$ | % of total | 95% CIs[c] | | Half-life[b] | 95% CIs[b,c] | | Half-life[b] | 95% CIs[b,c] | | |
| | | | Low | High | | | Low | High | | Low | High | | Low | High | |
| Ahn **[18]** | cis + doce | 9.3 | 8.8 | 9.7 | 0.98 | 95 | 84 | 97 | 8.2 | 7.4 | 8.7 | 7.3x10$^{13}$ | 32.6 | ? | 0.99 |
| Ahn **[18]** | cis + doce + consol | 10.1 | 9.5 | 10.7 | 0.97 | 91 | 87 | 93 | 8.0 | 7.5 | ? | 499.0 | 90.3 | ? | 0.99 |
| Atagi elderly **[19]** | daily carb | 9.6 | 9.1 | 10.1 | 0.95 | 91 | 88 | 93 | 7.8 | 7.4 | 8.2 | 180.3 | 104.2 | 649.7 | 0.99 |
| Bradley **[20]** | 74 Gy carb + pacl | 11.4 | 10.9 | 12.0 | 0.94 | 89 | 86 | 90 | 8.5 | 8.1 | ? | 5.4x10$^{15}$ | ? | ? | 0.97 |
| Bradley **[20]** | 60 Gy carb + pacl | 15.5 | 14.8 | 16.1 | 0.92 | 85 | 82 | ? | 10.2 | 9.7 | ? | 5.6x10$^{15}$ | ? | ? | 0.97 |
| Butts **[21]** | plat chemo | 11.8 | 10.9 | 12.8 | 0.88 | 79 | 75 | 82 | 6.5 | 6.1 | 6.9 | 197.7 | 100.2 | ? | 0.99 |
| Butts **[21]** | plat chemo + temcemotide | 13.9 | 13.0 | 14.7 | 0.89 | 79 | 75 | 81 | 7.8 | 7.2 | ? | 740.5 | 137.6 | ? | 0.99 |
| Carter **[22]** | carb + pacl + maintenance | 9.1 | 8.6 | 9.7 | 0.88 | 90 | 86 | 93 | 7.2 | 6.7 | 7.9 | 4.2x10$^{15}$ | ? | ? | 0.91 |
| Carter **[22]** | carb + pacl | 15.0 | 14.3 | 15.9 | 0.88 | 85 | 82 | 88 | 10.4 | 9.4 | 11.3 | 1.5x10$^{13}$ | 74.8 | ? | 0.92 |
| Chang **[23]** | proton + carb + pacl | 18.0 | 16.7 | 19.3 | 0.90 | 77 | 66 | 86 | 10.6 | 9.1 | 12.1 | 111.1 | 60.0 | 5239 | 0.97 |

| | | | | | | | | | | | | | | |
|---|---|---|---|---|---|---|---|---|---|---|---|---|---|
| Choy **[24]** | cis + pem + consol | 16.0 | 15.3 | 16.6 | 0.94 | 93 | ? | ? | 14.5 | ? | ? | 5.7x$10^{15}$ | ? | ? | 0.94 |
| Faivre-Finn **[25]** | plat chemo | 10.0 | 8.9 | 11.2 | 0.75 | 74 | 71 | 77 | 4.1 | 3.8 | 4.4 | 97.2 | 69.6 | 165.8 | 0.99 |
| Faivre-Finn **[25]** | plat chemo + durvalumab | 23.6 | 22.1 | 25.2 | 0.66 | 43 | 41 | 45 | 4.0 | 3.7 | 4.3 | 61.4 | 57.2 | 66.4 | 0.99 |
| Fenwick **[26]** | cis + vino | 25.9 | 25.0 | 26.9 | 0.94 | 67 | 44 | 79 | 13.5 | 9.9 | ? | 158.3 | 54.5 | ? | 0.97 |
| Flentje [27] | cis + vino + maintenance | 8.1 | 7.6 | 8.7 | 0.93 | 89 | 87 | 90 | 6.1 | 5.8 | ? | 4.9x$10^{15}$ | ? | ? | 0.98 |
| Flentje [27] | cis + vino | 7.2 | 6.5 | 7.9 | 0.90 | 78 | 74 | 82 | 4.3 | 4.0 | 4.6 | 68.0 | 47.3 | 123.3 | 0.99 |
| Fournel [28] | chemo induction + concur | 12.0 | 11.3 | 12.8 | 0.93 | 89 | 86 | 91 | 9.1 | 8.6 | ? | 4.6x$10^{15}$ | ? | ? | 0.97 |
| Fournel [28] | chemo concur + consol | 13.6 | 12.0 | 15.3 | 0.77 | 81 | 78 | 83 | 7.4 | 6.9 | ? | 4.0x$10^{15}$ | ? | ? | 0.94 |
| Garrido [29] | chemo induction + concur | 13.1 | 12.7 | 13.5 | 0.97 | 85 | 70 | 88 | 9.5 | 8.3 | ? | 5.8x$10^{15}$ | ? | ? | 0.98 |
| Glinski **[52]** | Cis + vino | 40.3 | 38.1 | 42.6 | 0.86 | 48 | 41 | 57 | 11.1 | 9.5 | 13.1 | 258.7 | 144.6 | ? | 0.99 |
| Govindan [30] | carb + pem + cetuximab | 13.7 | 13.2 | 14.1 | 0.96 | 90 | 78 | 93 | 10.9 | 9.7 | 11.7 | 9.4x$10^{13}$ | 45.0 | ? | 0.97 |
| Hoang [31] | carb + pacl + thalidomide | 8.4 | 8.1 | 8.7 | 0.97 | 95 | 87 | 99 | 7.6 | 7.1 | 8.2 | 6.1x$10^{15}$ | ? | ? | 0.98 |
| Hoang [31] | carb + pacl | 8.5 | 8.2 | 8.8 | 0.97 | 90 | 84 | 92 | 6.8 | 6.3 | 7.2 | 5.7x$10^{15}$ | ? | ? | 0.99 |
| Horin-ouchi [32] | chemo concur + consol | 16.9 | 15.3 | 18.5 | 0.89 | 87 | 85 | 88 | 11.4 | 10.8 | ? | 4.3x$10^{15}$ | ? | ? | 0.98 |
| Imamura [33] | hyperfrac + boost +chemo | 25.6 | 23.3 | 28.1 | 0.85 | 65 | 55 | 74 | 10.2 | 8.5 | 12.1 | 148.8 | 85.1 | 1227 | 0.97 |
| Isla [34] | cis + etop | 11.8 | 11.2 | 12.4 | 0.95 | 85 | 62 | 89 | 8.2 | 6.5 | ? | 185.0 | 28.4 | ? | 0.97 |
| Isla [34] | cis + vino | 13.8 | 13.3 | 14.4 | 0.95 | 85 | 83 | 86 | 9.5 | 9.0 | 9.9 | 5.8x$10^{11}$ | 66.4 | ? | 0.98 |
| Kawaguchi [35] | chemo concur + consol | 12.8 | 12.2 | 13.4 | 0.94 | 88 | 84 | 91 | 9.7 | 9.0 | ? | 3.8x$10^{15}$ | ? | ? | 0.96 |
| Kerner [36] | chemo induction + concur | 24.1 | 22.2 | 26.2 | 0.85 | 80 | 75 | 82 | 13.6 | 12.5 | ? | 3.7x$10^{15}$ | ? | ? | 0.96 |
| Landau [93] | Cis + vino | 23.5 | 22.6 | 24.3 | 0.95 | 9 | ? | ? | 12.4 | ? | ? | 25.0 | ? | ? | 0.95 |
| Lawrence [37] | hyperfrac + chemo + amifostine | 12.1 | 11.4 | 12.8 | 0.94 | 88 | 82 | 90 | 9.1 | 8.3 | ? | 5.9x$10^{15}$ | ? | ? | 0.97 |
| Lawrence [37] | hyperfrac + chemo | 12.6 | 12.1 | 13.1 | 0.97 | 91 | 82 | 93 | 10.0 | 9.0 | ? | 5.9x$10^{15}$ | ? | ? | 0.99 |
| Lerouge [38] | chemo induction + concur | 19.7 | 18.8 | 20.6 | 0.91 | 75 | 70 | 78 | 10.7 | 9.9 | ? | 3.7x$10^{15}$ | ? | ? | 0.98 |
| Liang [39] | cis + pacl | 11.6 | 10.9 | 12.4 | 0.92 | 90 | 87 | 92 | 9.3 | 8.7 | ? | 3.4x$10^{15}$ | ? | ? | 0.95 |
| Liang [39] | cis + etop | 16.6 | 15.6 | 17.6 | 0.93 | 86 | 83 | 88 | 11.8 | 11.1 | 12.6 | 5.6x$10^{15}$ | ? | ? | 0.97 |
| Lu [40] | chemo + AE-941 | 18.2 | 16.3 | 20.3 | 0.67 | 67 | 64 | 70 | 5.9 | 5.6 | 6.3 | 205.5 | 138.6 | 416.7 | 0.99 |
| Lu [40] | chemo induction + concur | 21.6 | 19.7 | 23.6 | 0.72 | 67 | 63 | 71 | 7.5 | 6.9 | 8.2 | 379.6 | 166.5 | ? | 0.98 |
| Niho [41] | cis + pem | 16.1 | 15.3 | 17.0 | 0.94 | 87 | 84 | 91 | 11.8 | 10.6 | 13.2 | 5.1x$10^{11}$ | 39.5 | ? | 0.96 |
| Niho [41] | cis + S-1 | 21.2 | 18.9 | 23.8 | 0.63 | 70 | 67 | 72 | 8.0 | 7.3 | ? | 3.6x$10^{15}$ | ? | ? | 0.95 |

| | | | | | | | | | | | | | | | |
|---|---|---|---|---|---|---|---|---|---|---|---|---|---|---|---|
| Park [42] | chemo- EGFR unknown | 14.1 | 13.0 | 15.2 | 0.82 | 76 | 75 | 78 | 7.1 | 6.8 | ? | 5.6x$10^{15}$ | ? | ? | 0.97 |
| Park [42] | chemo- EGFR WT | 15.6 | 14.8 | 16.4 | 0.91 | 83 | 72 | 85 | 9.9 | 8.6 | 10.6 | 3.2x$10^{15}$ | ? | ? | 0.97 |
| Price [43] | Gemcit | 16.9 | 15.3 | 18.7 | 0.94 | 27 | 21 | 36 | 5.5 | 4.1 | 7.4 | 35.2 | 32.0 | 39.8 | 0.96 |
| Provencio [44] | chemo induction + concur | 13.2 | 12.8 | 13.6 | 0.97 | 94 | 90 | 99 | 11.7 | 10.6 | ? | 7.7x$10^{12}$ | 20.7 | ? | 0.97 |
| Sasaki [45] | cis + vino | 13.6 | 12.9 | 14.3 | 0.93 | 89 | 83 | 94 | 10.9 | 9.8 | ? | 3.2x$10^{15}$ | ? | ? | 0.94 |
| Sasaki [45] | cis + S-1 | 16.4 | 15.8 | 17.1 | 0.90 | 79 | 78 | ? | 9.3 | 9.1 | ? | 4.6x$10^{10}$ | 137.0 | ? | 0.96 |
| Senan [46] | cis + etop | 12.0 | 11.3 | 12.7 | 0.96 | 92 | 88 | 94 | 9.7 | 9.1 | ? | 5.0x$10^{15}$ | ? | ? | 0.98 |
| Senan [46] | cis + pem | 14.1 | 13.3 | 14.9 | 0.95 | 89 | 85 | 91 | 10.3 | 9.6 | ? | 5.0x$10^{15}$ | ? | ? | 0.98 |
| Shimokawa [47] | cis + S-1 | 14.3 | 13.7 | 14.8 | 0.94 | 88 | 81 | 91 | 10.9 | 10.1 | 11.8 | 4.6x$10^{15}$ | ? | ? | 0.96 |
| Shimokawa [47] | Cis + doce | 21.0 | 20.6 | 21.3 | 0.99 | 4 | 0.2 | ? | 5.2 | 1.3 | ? | 21.8 | 20.9 | 73.3 | 0.99 |
| Tsuchiya-Kawano **[88]** | Carb + nab-pacl | 14.6 | 14.1 | 15.2 | 0.95 | No 2-phase model fit | | | | | | | | | |
| van Baardwijk **[48]** | chemo induction + concur | 19.7 | 18.9 | 20.7 | 0.93 | 80 | 73 | 83 | 15.7 | 11.5 | 13.8 | 4.7x$10^{15}$ | ? | ? | 0.96 |
| Vera **[49]** | chemo induction + concur | 16.5 | 16.0 | 16.9 | 0.96 | 89 | 77 | 93 | 13.3 | 12.3 | ? | 1.9x$10^{13}$ | 52.2 | ? | 0.97 |
| Wada **[50]** | 60 Gy + chemo | 10.6 | 9.7 | 11.7 | 0.83 | 84 | 82 | 86 | 7.3 | 6.8 | ? | 4.3x$10^{15}$ | ? | ? | 0.95 |
| Wada **[50]** | hyperfrac + chemo | 21.5 | 19.7 | 23.4 | 0.81 | 76 | 72 | 78 | 10.5 | 9.7 | 11.2 | 4.4x$10^{15}$ | ? | ? | 0.97 |
| Yamamoto **[51]** | carb + irinotecan | 8.9 | 8.2 | 9.6 | 0.88 | 89 | 88 | 89 | 6.3 | 6.0 | 6.5 | 1.2x$10^{11}$ | 179.8 | ? | 0.98 |
| Yamamoto**[51]** | cis + vindesine + mito | 10.2 | 9.4 | 10.9 | 0.89 | 88 | 86 | 89 | 7.1 | 6.7 | ? | 5.4x$10^{15}$ | ? | ? | 0.97 |
| Yamamoto **[51]** | carb + pacl | 11.0 | 10.4 | 11.7 | 0.91 | 88 | 87 | 90 | 7.9 | 7.5 | ? | 3.3x$10^{15}$ | ? | ? | 0.98 |

a. cis: cisplatin; doce: docetaxel; consol: consolidation chemotherapy; carb: carboplatin; pacl: paclitaxel; plat: platinum; chemo: chemotherapy; pem: pemetrexed; vino: vinorelbine; concur: concurrent chemotherapy; hyperfrac: hyperfractionated radiotherapy; etop: etoposide; EGFR WT: EGFR wild type; mito: myomycin-C
b. months
c. CIs: confidence intervals
? : could not be defined

Table S3. PFS population survival kinetics assessment of limited small cell lung cancer

| Study | Arm[a] | 1-phase decay models | | | | 2-phase decay models | | | | | | | | | |
|---|---|---|---|---|---|---|---|---|---|---|---|---|---|---|---|
| | | | | | | Relapsing subpopulation | | | | | | Potentially cured subpopulation | | | $R^2$ |
| | | Overall half-life[b] | 95% CIs[b,c] | | $R^2$ | % of total | 95% CIs[c] | | Half-life[b] | 95% CIs[b,c] | | Half-life[b] | 95% CIs[b,c] | | |
| | | | Low | High | | | Low | High | | Low | High | | Low | High | |
| Arriagada **[54]** | XRT 45-55Gy | 16.3 | 15.3 | 17.3 | 0.91 | 88 | 77 | 93 | 12.4 | 10.9 | ? | 4.4x$10^{15}$ | ? | ? | 0.93 |
| Arriagada **[54]** | XRT 65Gy | 15.0 | 14.1 | 16.0 | 0.92 | no 2-phase model fit | | | | | | | | | |

| | | | | | | | | | | | | | | | |
|---|---|---|---|---|---|---|---|---|---|---|---|---|---|---|---|
| Arriagada **[53]** | Alternating chemo/XRT | 15.2 | 14.4 | 16.0 | 0.95 | 88 | 86 | 89 | 11.0 | 10.6 | ? | 4.3x10$^{15}$ | ? | ? | 0.99 |
| Beith **[55]** | maintenance | 17.4 | 15.0 | 20.2 | 0.76 | 82 | 80 | 84 | 10.1 | 9.7 | 10.6 | 666 | 380.0 | 2845.0 | 0.99 |
| Beith **[55]** | no maintenance | 9.0 | 8.0 | 10.1 | 0.9 | 75 | 72 | 78 | 5.4 | 5.0 | 5.8 | 86.1 | 72.3 | 105.7 | 0.99 |
| Blackstock **[56]** | concurrent XRT | 10.7 | 9.9 | 11.7 | 0.94 | 82 | 75 | 88 | 7.4 | 6.6 | 8.3 | 105.8 | 63.3 | 330.7 | 0.98 |
| Blackstock **[56]** | split course XRT | 12.2 | 11.5 | 12.9 | 0.97 | 93 | 91 | 96 | 10.5 | 9.7 | 11.4 | 1.3x10$^{12}$ | 33.3 | ? | 0.97 |
| Bogart **[57]** | concurrent XRT | 15.1 | 14.7 | 15.6 | 0.96 | 91 | 60 | 98 | 12.9 | 10.9 | ? | 3.9x10$^{15}$ | ? | ? | 0.96 |
| Bonner **[58]** | once daily XRT | 17.8 | 17.3 | 18.4 | 0.96 | 88 | 83 | ? | 14.2 | ? | ? | 6.2x10$^{11}$ | 19.7 | ? | 0.96 |
| Bonner **[58]** | twice daily XRT | 16.8 | 16.3 | 17.4 | 0.95 | no 2-phase model fit, but 2-phase decay on log-linear plot | | | | | | | | | |
| Edelman **[59]** | concurrent XRT | 11.6 | 11.0 | 12.2 | 0.92 | 90 | 84 | 93 | 9.0 | 8.3 | ? | 4.8x10$^{15}$ | ? | ? | 0.94 |
| Ettinger **[60]** | hyperfract-ionated XRT | 16.8 | 15.8 | 17.9 | 0.91 | 84 | 80 | 87 | 11.2 | 10.4 | ? | 4.2x10$^{15}$ | ? | ? | 0.96 |
| Glisson **[61]** | hyperfract-ionated XRT | 25.5 | 25.0 | 26.1 | 0.98 | no 2-phase model fit | | | | | | | | | |
| Goodman **[62]** | CAV+etop | 17.3 | 16.5 | 18.1 | 0.96 | 95 | ? | ? | 15.6 | 14.0 | ? | 9.0x10$^{13}$ | ? | ? | 0.96 |
| Goodman **[62]** | cis-etop/CAV alternating | 16.4 | 15.7 | 17.1 | 0.96 | 93 | 72 | 97 | 14.3 | 12.4 | ? | 3.8x10$^{15}$ | ? | ? | 0.97 |
| Gregor **[63]** | sequential XRT | 12.7 | 12.1 | 13.4 | 0.94 | 92 | 87 | 95 | 10.6 | 9.7 | ? | 3.7x10$^{15}$ | ? | ? | 0.95 |
| Gregor **[63]** | alternating chemo/XRT | 10.2 | 9.8 | 10.7 | 0.95 | 96 | 93 | 97 | 9.2 | 8.7 | ? | 4.5x10$^{15}$ | ? | ? | 0.96 |
| Gronberg **[64]** | hyperfract-ionated 45Gy | 17.7 | 16.2 | 19.3 | 0.84 | 77 | 69 | 80 | 9.7 | 8.5 | ? | 5.2x10$^{15}$ | ? | ? | 0.94 |
| Gronberg **[64]** | hyperfract-ionated 60Gy | 25.4 | 24.1 | 26.9 | 0.91 | 75 | 58 | 78 | 13.9 | 11.3 | ? | 5.1x10$^{15}$ | ? | ? | 0.97 |
| Halvorsen **[65]** | comorbid | 11.2 | 10.0 | 12.4 | 0.9 | 87 | 84 | 91 | 8.0 | 7.1 | 9.0 | 2.3x10$^{13}$ | 52.3 | ? | 0.94 |
| Halvorsen **[65]** | no comorbid | 17.3 | 16.0 | 18.7 | 0.92 | 86 | 69 | 89 | 12.4 | 10.3 | 14.4 | 3.8x10$^{15}$ | ? | ? | 0.94 |
| Horn **[66]** | concurrent XRT | 16.3 | 15.4 | 17.2 | 0.92 | no 2-phase model fit | | | | | | | | | |
| Hugli **[67]** | hyperfract-ionated XRT | 17.9 | 16.2 | 19.8 | 0.75 | 75 | 71 | 76 | 7.8 | 7.1 | 8.4 | 3.3x10$^{15}$ | ? | ? | 0.96 |
| Jett **[68]** | CAV | 8.4 | 7.8 | 9.0 | 0.92 | 93 | 90 | 95 | 7.3 | 6.7 | ? | 5.3x10$^{15}$ | ? | ? | 0.94 |
| Jett **[68]** | CAV+etop | 11.5 | 10.6 | 12.5 | 0.9 | 88 | 83 | 89 | 8.6 | 7.9 | 9.0 | 7.3x10$^{14}$ | 169.1 | ? | 0.98 |
| Kelley **[69]** | concurrent XRT | 13.3 | 12.6 | 14.0 | 0.95 | 89 | 86 | 94 | 10.3 | 9.3 | 11.6 | 1.3x10$^{12}$ | 40.7 | ? | 0.96 |
| Komaki **[70]** | hyperfract-ionated XRT | 11.5 | 10.9 | 12.2 | 0.95 | 94 | 77 | 99 | 10.3 | 9.1 | ? | 4.2x10$^{15}$ | ? | ? | 0.95 |
| Kubota **[71]** | cis-etop | 21.2 | 18.9 | 23.6 | 0.82 | 70 | 54 | 84 | 9.8 | 7.4 | 12.7 | 105.1 | 52.0 | ? | 0.94 |
| Kubota **[71]** | cis-etop/ cis-irinotecan | 23.6 | 20.4 | 27.0 | 0.67 | 76 | 66 | 78 | 9.9 | 8.2 | ? | 3.7x10$^{15}$ | ? | ? | 0.93 |
| Laack **[73]** | concurrent XRT | 7.7 | 7.3 | 8.1 | 0.96 | no 2-phase model fit | | | | | | | | | |
| Le **[72]** | Tirapazamine /XRT | 13.5 | 12.9 | 14.2 | 0.95 | 92 | 42 | 97 | 11.2 | 7.8 | ? | 4.9x10$^{15}$ | ? | ? | 0.96 |
| Mano-Haran **[74]** | no PET scan | 23.3 | 21.2 | 25.6 | 0.79 | 78 | 73 | 81 | 12.3 | 11.1 | 13.7 | 4.4x10$^{15}$ | ? | ? | 0.93 |
| Mano-Haran **[74]** | PET scan | 28.3 | 26.0 | 30.8 | 0.81 | 74 | 68 | 76 | 13.0 | 11.8 | ? | 5.0x10$^{15}$ | ? | ? | 0.96 |
| Maurer **[75]** | no warfarin | 14.6 | 13.8 | 15.4 | 0.95 | 91 | 87 | 93 | 11.4 | 10.7 | 12.0 | 4.1x10$^{15}$ | ? | ? | 0.98 |
| Maurer **[75]** | warfarin | 23.0 | 21.6 | 24.5 | 0.88 | 70 | 64 | 77 | 10.8 | 9.6 | 12.0 | 138.5 | 88.8 | 380.7 | 0.99 |

| | | | | | | | | | | | | | | | |
|---|---|---|---|---|---|---|---|---|---|---|---|---|---|---|---|
| McClay **[76]** | No tamoxifen | 16.5 | 15.5 | 17.5 | 0.92 | 88 | 86 | 90 | 11.8 | 11.1 | 12.5 | 2.0x10$^{13}$ | 113.4 | ? | 0.97 |
| McClay **[76]** | Tamoxifen | 13.8 | 13.1 | 14.6 | 0.93 | 90 | 75 | 95 | 11.2 | 9.5 | ? | 184.7 | 41.0 | ? | 0.95 |
| Miller **[77]** | concurrent XRT | 15.0 | 14.1 | 16.0 | 0.86 | 79 | 77 | 81 | 8.4 | 7.8 | ? | 5.1x10$^{15}$ | ? | ? | 0.96 |
| Murray **[78]** | early XRT | 18.9 | 17.7 | 20.2 | 0.9 | 88 | 82 | 96 | 14.9 | 12.9 | 17.7 | 2.2x10$^{13}$ | 40.5 | ? | 0.91 |
| Murray **[78]** | late XRT | 14.6 | 13.7 | 15.7 | 0.91 | 91 | 73 | 97 | 12.3 | 10.6 | ? | 3.3x10$^{15}$ | ? | ? | 0.92 |
| Perry **[79]** | early XRT | 11.2 | 10.9 | 11.6 | 0.99 | no 2-phase model fit, but 2-phase decay on log-linear plot | | | | | | | | | |
| Perry **[79]** | late XRT | 13.1 | 12.6 | 13.7 | 0.98 | 97 | 90 | 99 | 12.4 | 11.4 | ? | 542.4 | 44.3 | ? | 0.98 |
| Perry **[79]** | no XRT | 8.2 | 7.9 | 8.6 | 0.97 | no 2-phase model fit, but 2-phase decay on log-linear plot | | | | | | | | | |
| Qiu **[80]** | hyperfract-ionated XRT | 16.8 | 15.7 | 17.9 | 0.93 | 89 | 75 | 94 | 12.9 | 11.2 | ? | 3.4x10$^{15}$ | ? | ? | 0.94 |
| Qiu **[80]** | once daily XRT | 26.5 | 24.7 | 28.6 | 0.84 | 70 | 62 | 75 | 13.3 | 11.4 | 15.4 | 3.7x10$^{15}$ | ? | ? | 0.92 |
| Salama **[81]** | concurrent XRT | 16.1 | 14.2 | 18.2 | 0.83 | 80 | 75 | 83 | 8.5 | 7.7 | ? | 361.3 | 131.5 | ? | 0.98 |
| Schild **[82]** | daily XRT | 16.7 | 15.9 | 17.5 | 0.93 | 84 | 80 | 86 | 11.2 | 10.5 | ? | 6.0x10$^{15}$ | ? | ? | 0.97 |
| Schild **[82]** | twice daily XRT | 17.6 | 16.7 | 18.6 | 0.91 | 81 | 79 | 84 | 10.9 | 10.1 | 11.8 | 1.7x10$^{13}$ | 200.2 | ? | 0.96 |
| Spiro **[83]** | early XRT | 12.6 | 11.0 | 14.4 | 0.81 | 85 | 83 | 86 | 7.9 | 7.4 | ? | 3.3x10$^{13}$ | ? | ? | 0.97 |
| Spiro **[83]** | late XRT | 16.2 | 14.5 | 18.1 | 0.87 | 84 | 79 | 85 | 9.7 | 8.9 | ? | 4.6x10$^{15}$ | ? | ? | 0.98 |
| Takada **[84]** | concurrent XRT | 16.7 | 15.2 | 18.2 | 0.87 | 83 | 79 | 85 | 10.4 | 9.6 | ? | 6.1x10$^{15}$ | ? | ? | 0.96 |
| Takada **[84]** | late XRT | 11.1 | 10.2 | 12.1 | 0.9 | 90 | 84 | 92 | 8.8 | 8.0 | 9.5 | 3.7x10$^{15}$ | ? | ? | 0.94 |
| Thomas **[85]** | SW-9229 | 10.8 | 10.2 | 11.3 | 0.95 | 95 | ? | ? | 9.8 | ? | 11.2 | 4.1x10$^{15}$ | ? | ? | 0.95 |
| Thomas **[86]** | SW-8269 | 18.2 | 16.1 | 20.5 | 0.92 | 59 | 54 | 64 | 7.5 | 6.7 | 8.3 | 59.5 | 52.5 | 68.6 | 0.99 |
| Xia **[87]** | XRT | 30.0 | 27.1 | 31.1 | 0.87 | 67 | 56 | 71 | 12.9 | 10.9 | 14.6 | 3.7x10$^{15}$ | ? | ? | 0.96 |

a. all groups received chemotherapy; XRT: radiation; Gy: Grays; CAV: cyclophosphamide + doxorubicin + vincristine; etop: etoposide; cis: cisplatin; comorbid: comorbidities

b. months

c. 95% confidence intervals

d. could not be fit to an EDNLRA

? could not be determined

**Tutorial S1 on Population Survival Kinetics Methodology:**

Population survival kinetics analyses can be useful in supplementing standard statistical analyses of clinical trials data. These analyses assume that progression-free survival (PFS) and overall survival (OS) curves generally approximate first-order kinetics. On log-linear plots, deviations of these curves from a straight line can offer biological insights into population behavior and factors that might influence this, and they can be useful in generating and testing hypotheses. Population survival kinetics analyses use exponential decay nonlinear regression analysis to calculate progression-free survival (PFS) and overall survival (OS) half-lives (time to progression or death of half the remaining patients), and these half-lives can be useful in performing further calculations.

Exponential decay nonlinear regression analyses can also assess whether PFS and OS curve data can be fit by 2-phase exponential decay models. If the curve can be fit by 2-phase decay models, this suggests that there are two distinct subpopulations with differing rates of tumor progression or death. The models can estimate the relative size of the two subpopulations and can estimate the PFS or OS half-lives for the rapidly progressing and slowly progressing subpopulations. For curves fitting 2-phase decay models, log-linear plots typically demonstrate a curve inflection point to the right.

As is generally the case with assessment of clinical research data, confidence in the interpretation of population survival kinetics results may vary with number of patients on the trial(s), length and maturity of patient follow up, model $R^2$ values, width of 95% confidence intervals, and consistency of observations across different trials of a therapy. If patient follow-up is relatively short, then 95% confidence intervals for parameter upper and lower boundaries may be very wide or undefinable, while they may be narrow with longer follow-up.

As a starting point for interested investigators, clinicians, and trainees, we have provided details on proposed population survival kinetics methodologies below. We have not compared our approaches to other potential approaches that might also be considered. For this illustration, we are using PFS data from patient populations with a potentially cured subpopulation, but the same approaches also apply for overall survival and for patients with incurable metastatic disease. We will start by illustrating how to digitize a published curve, and will follow with a discussion of exponential decay nonlinear regression analysis of the digitized data.

**How to digitize a published survival curve:**

1. Access the online data digitizing program https://apps.automeris.io/wpd/ (Copyright 2010-2022 Ankit Rohatgi) (or similar program) to digitize curves.

2. Save a screenshot of a survival curve of interest as a PNG file. Other file types may not work with this data digitizing program.

3. When opened, the online program appears as follows:

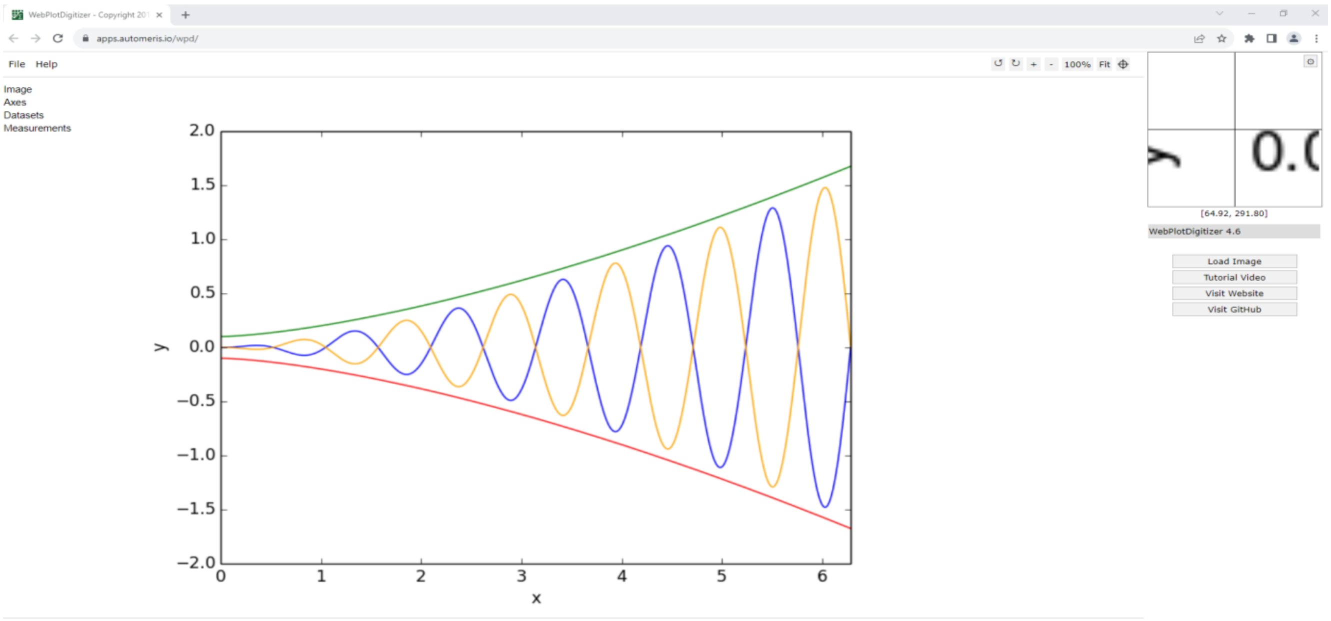

4. To load the curve of interest, click on “File” on the top left. A drop-down menu will appear. From this drop-down menu, click on “Load images”:

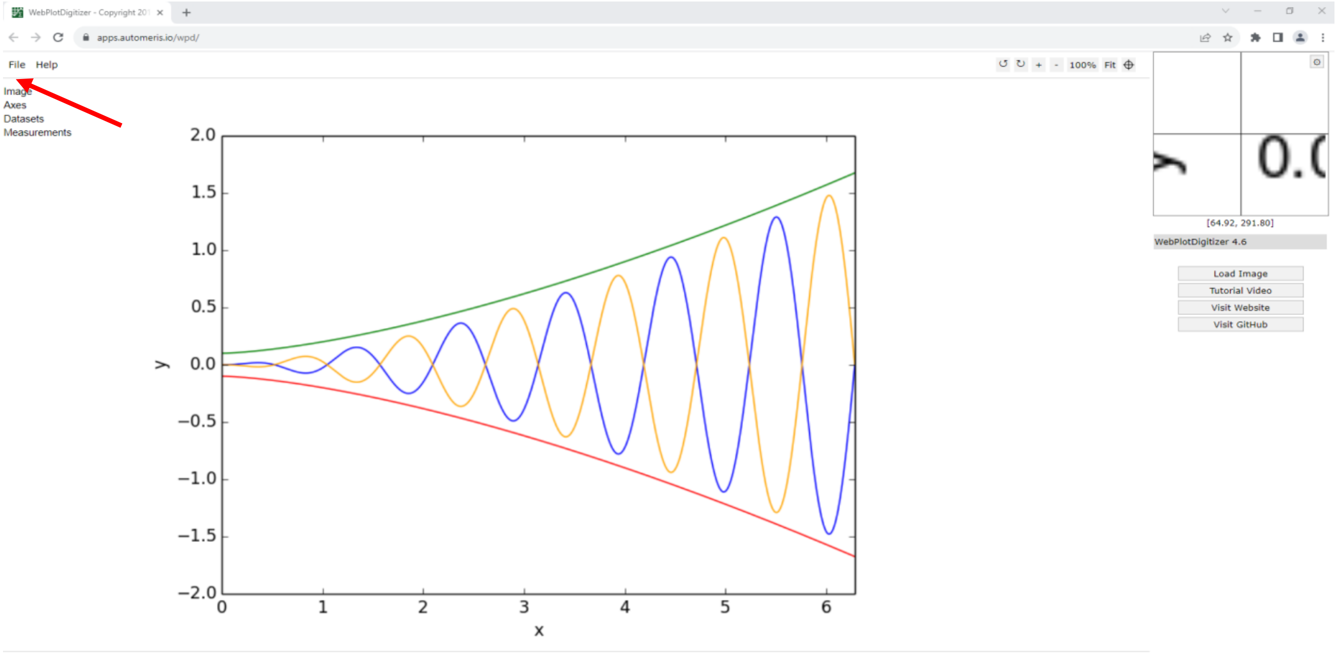


5. Click on Choose File:

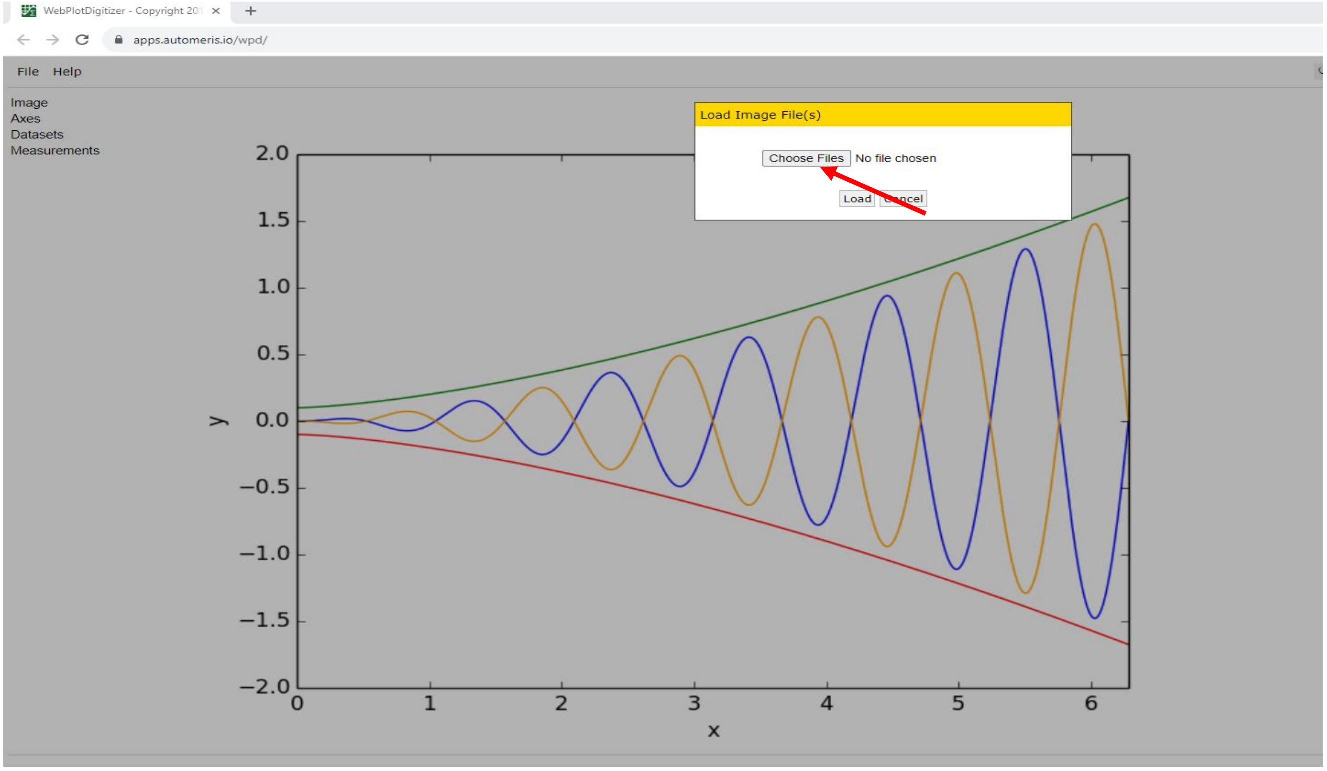


6. Go to files where PNG images are stored and click on the relevant PNG file:

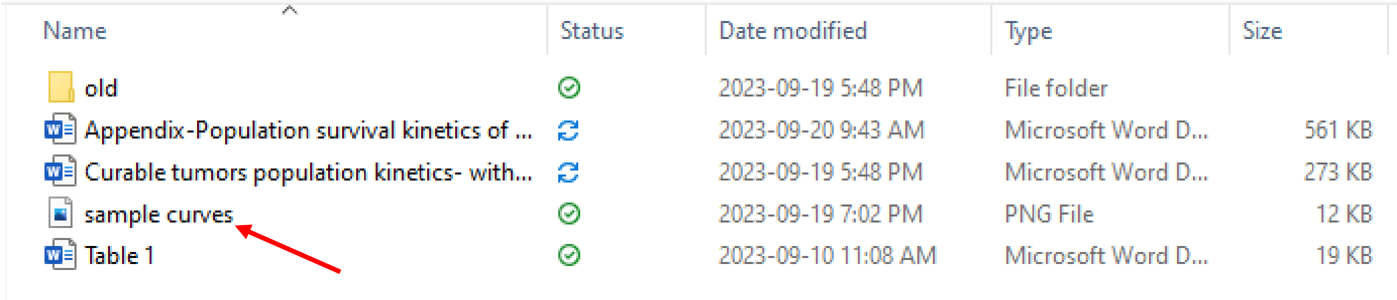


7. Our sample PNG file then appears:

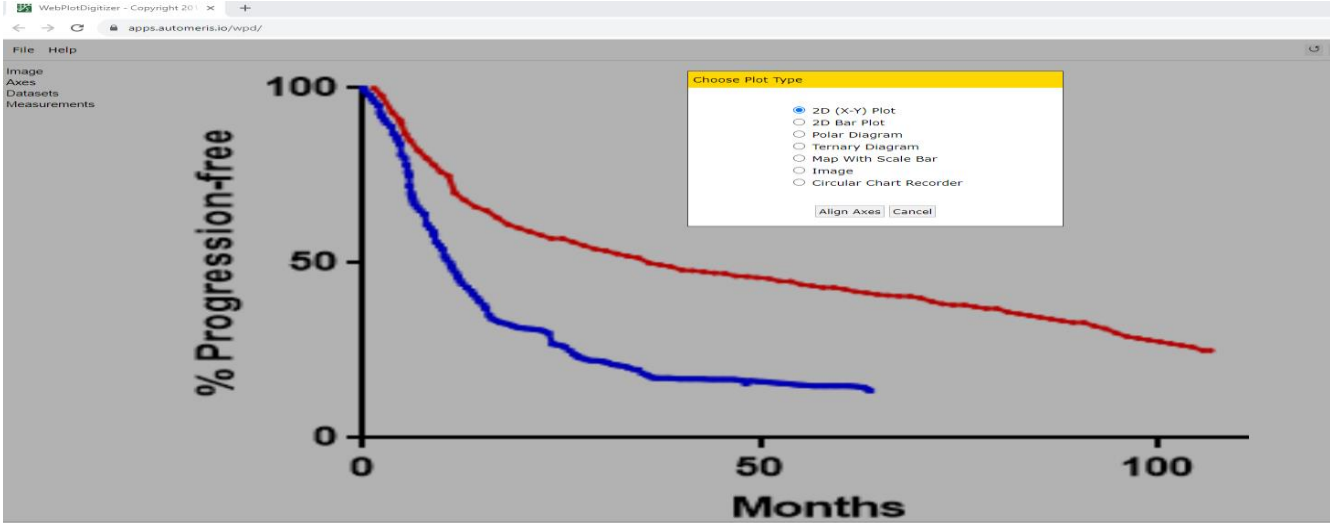


8. Click on “Align Axes”. This will set the scale that permits the program to define the proper co-ordinates for each point on the curve.

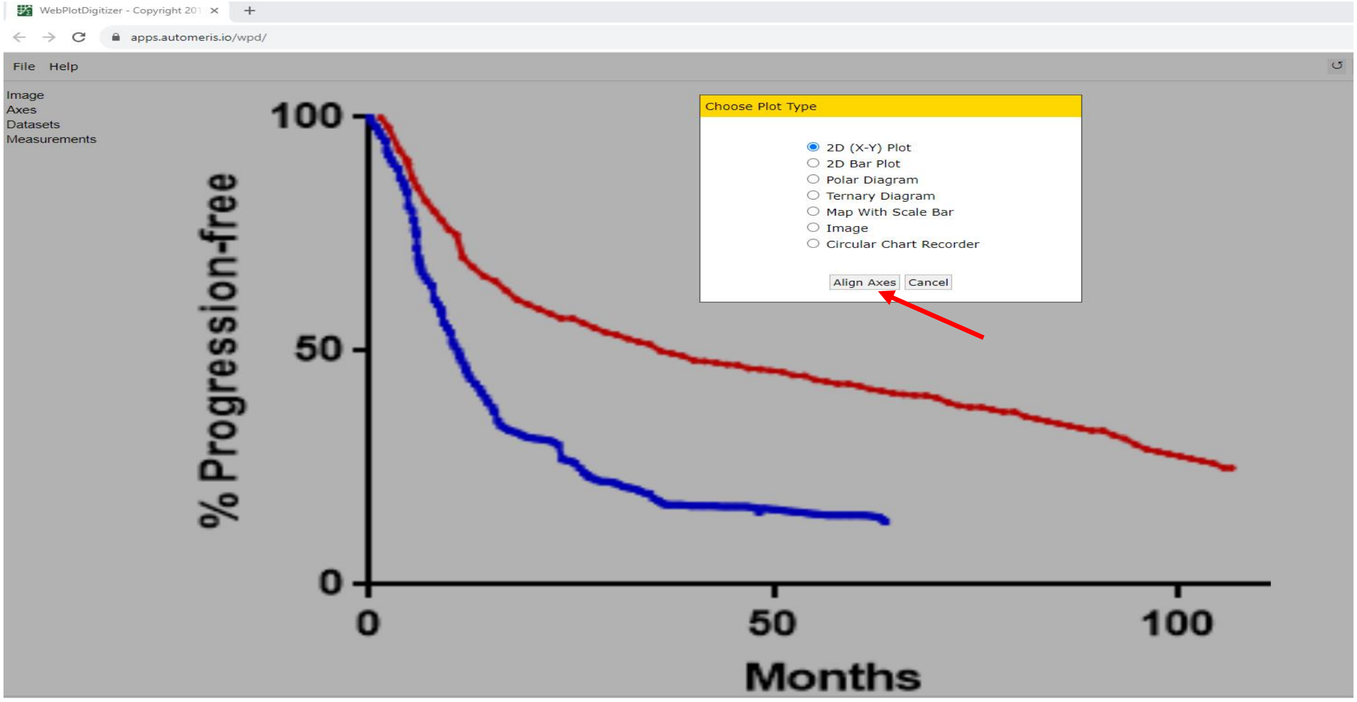

9. Click on “Proceed”

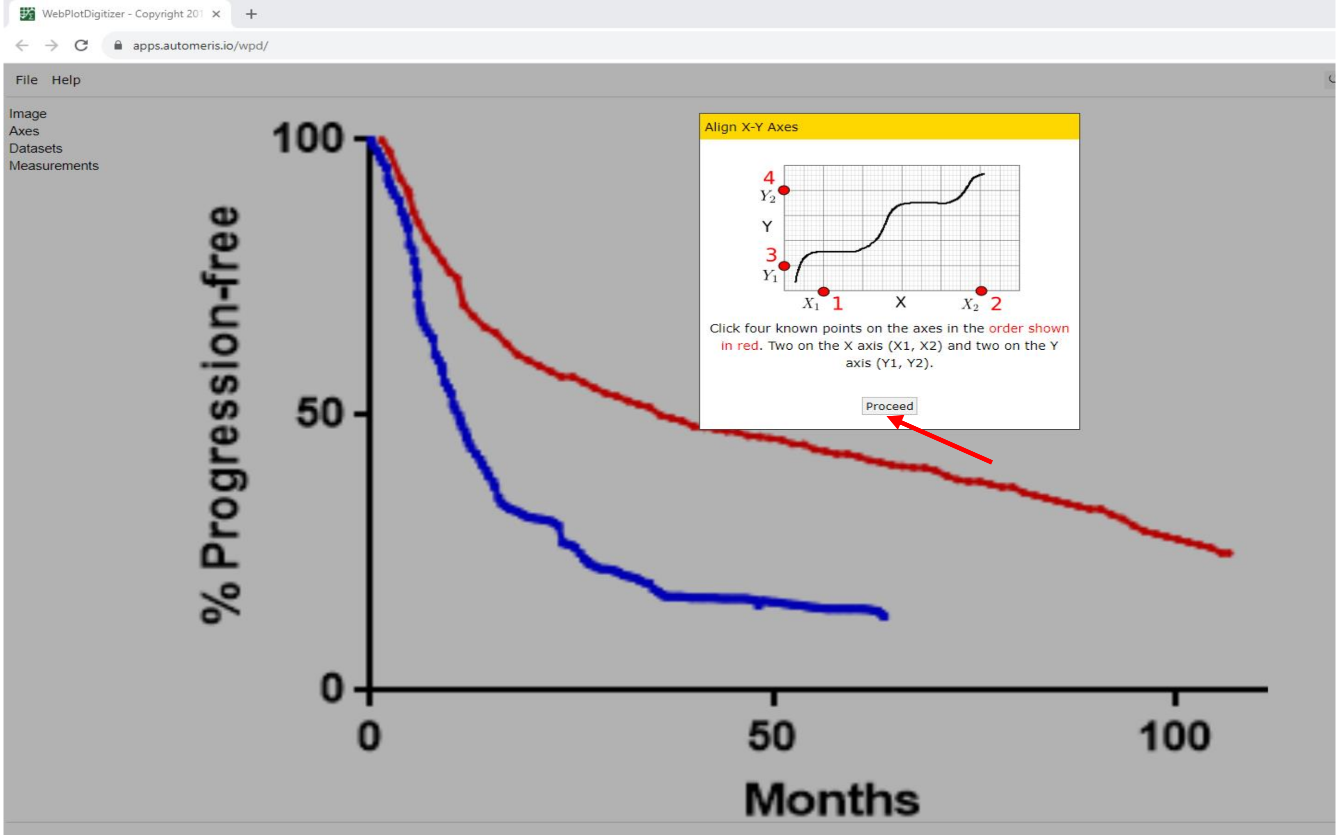


10. Click on 2 points on the x-axis (x1 and x2) and 2 points on the y-axis (y1 and y2):

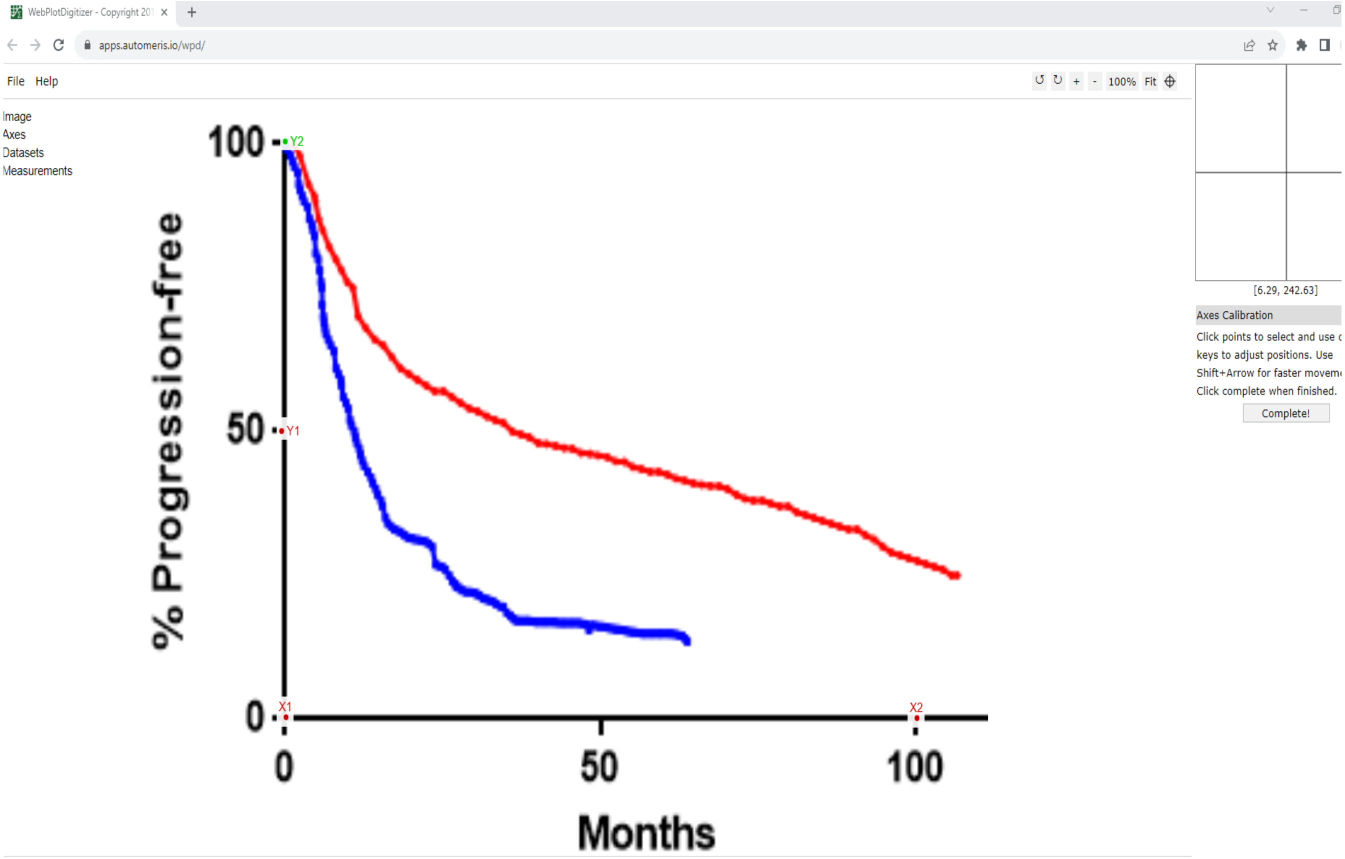


11. Click on “complete”:

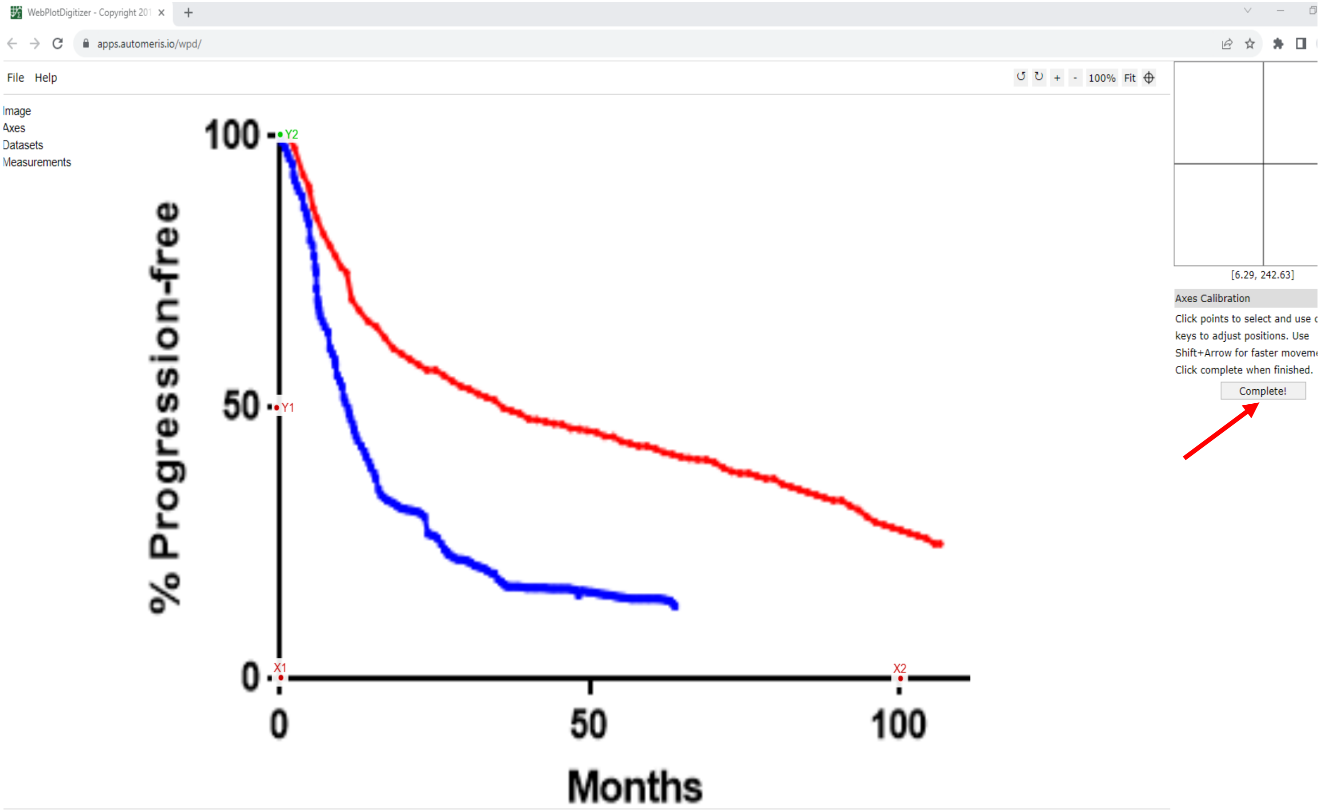


12. Fill in the values for Point 1 and Point 2, based on what you picked:

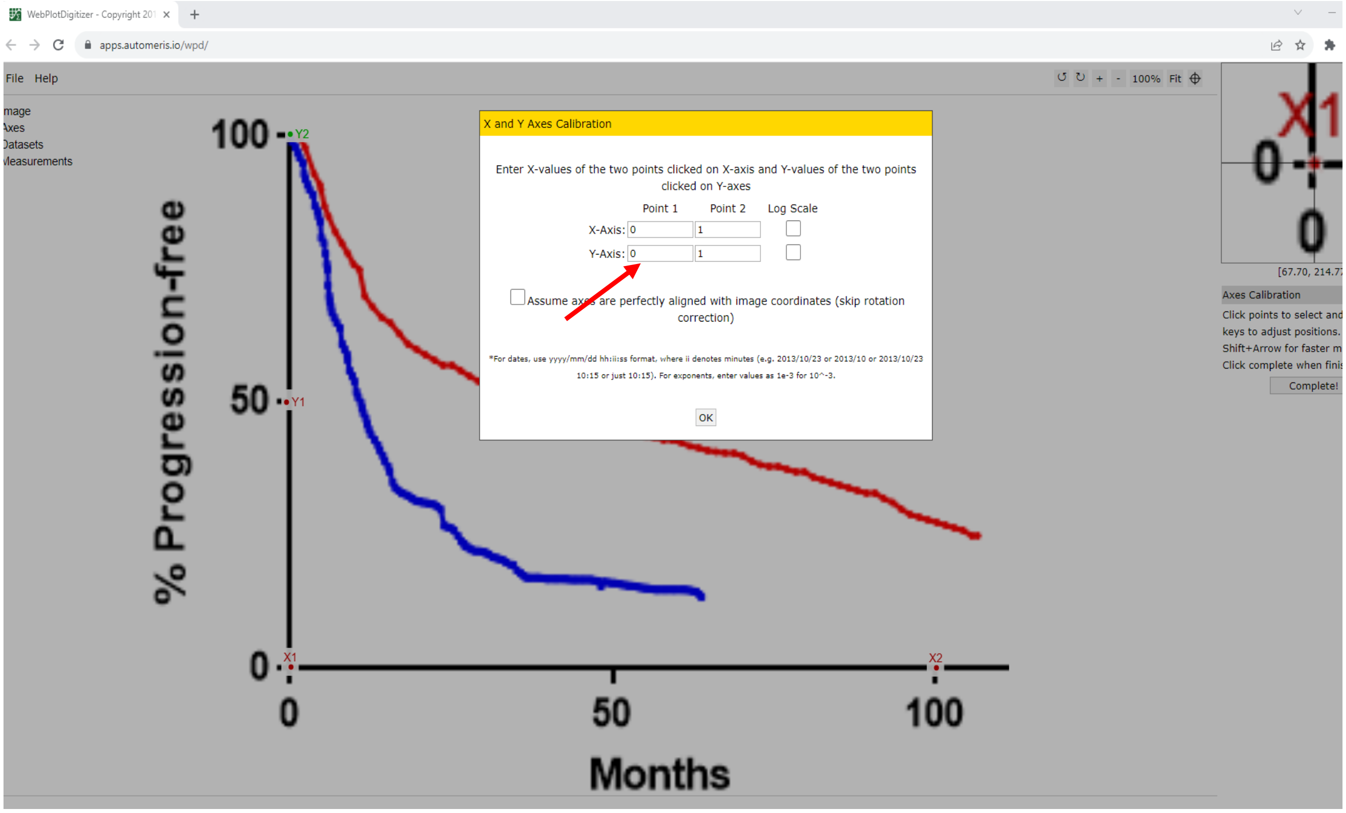


13. After the correct values have been inserted, click OK:

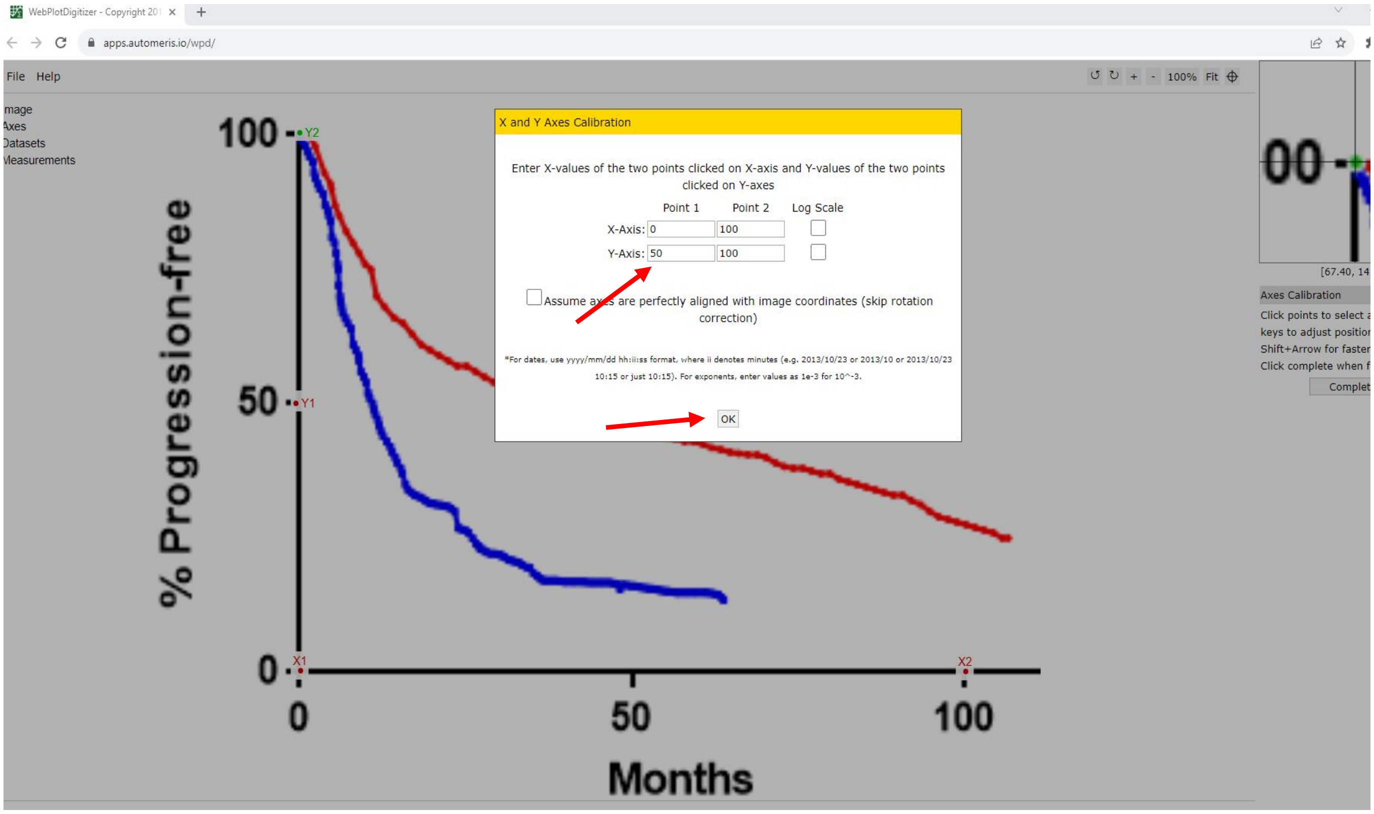


14. Rename the dataset, based on which curve you will analyze next.

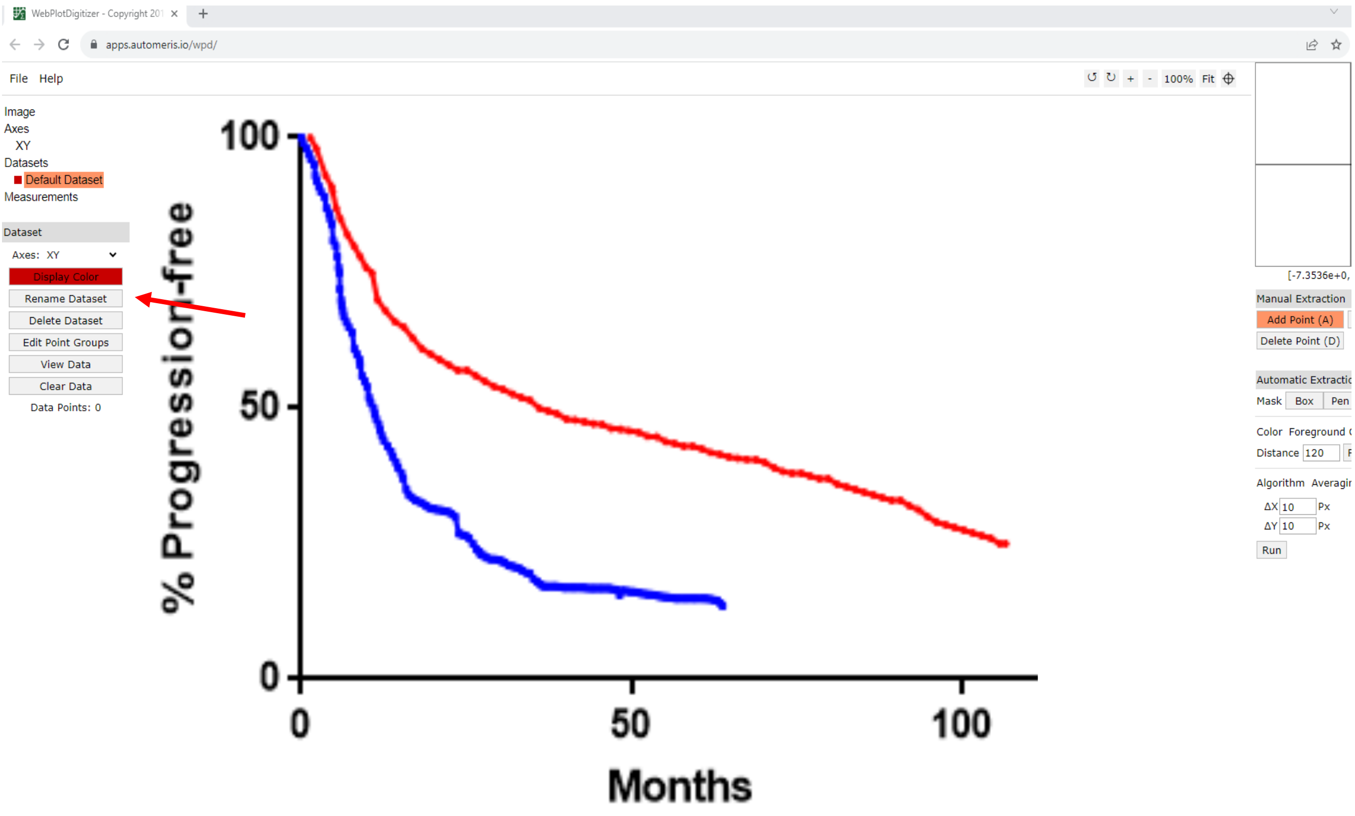


15. We will call the blue curve "Sample set A". Insert that name in the box, then hit "Rename":

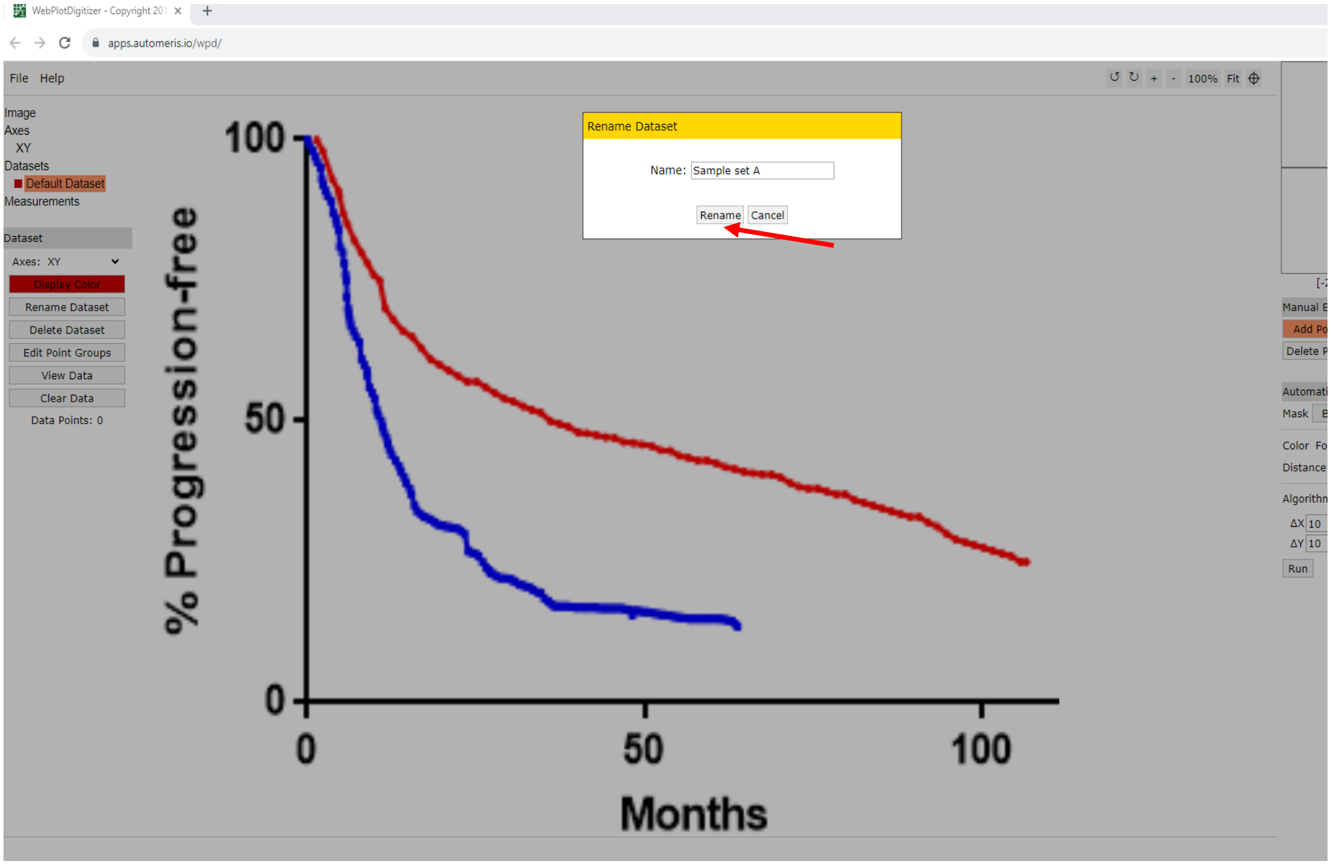


16. Then select the color of the first curve. Start by clicking on the color box:

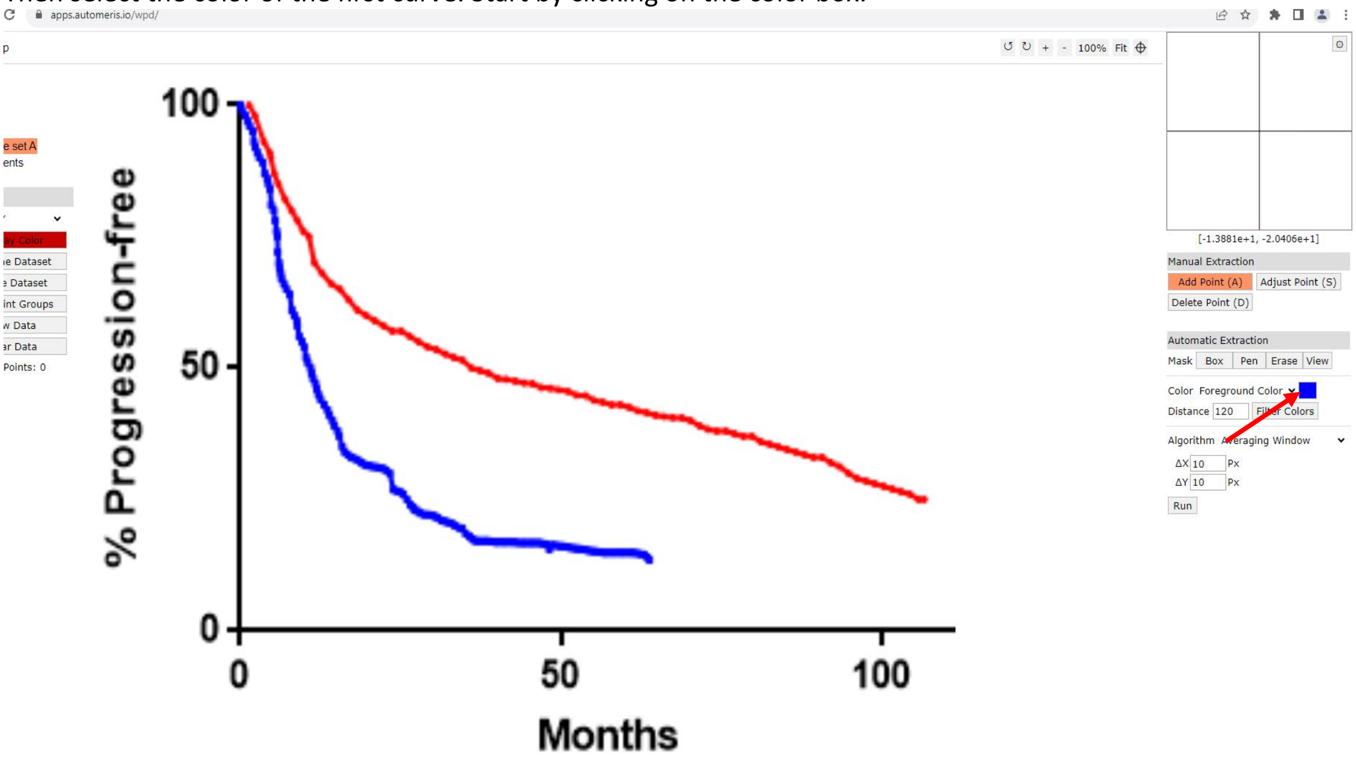


17. Then click on either the correct color as identified by the program (in this case, the blue option, which is also the program's default color), or else click on "Color Picker" to specifically target the color of one curve if you are not happy with the options offered by the program:

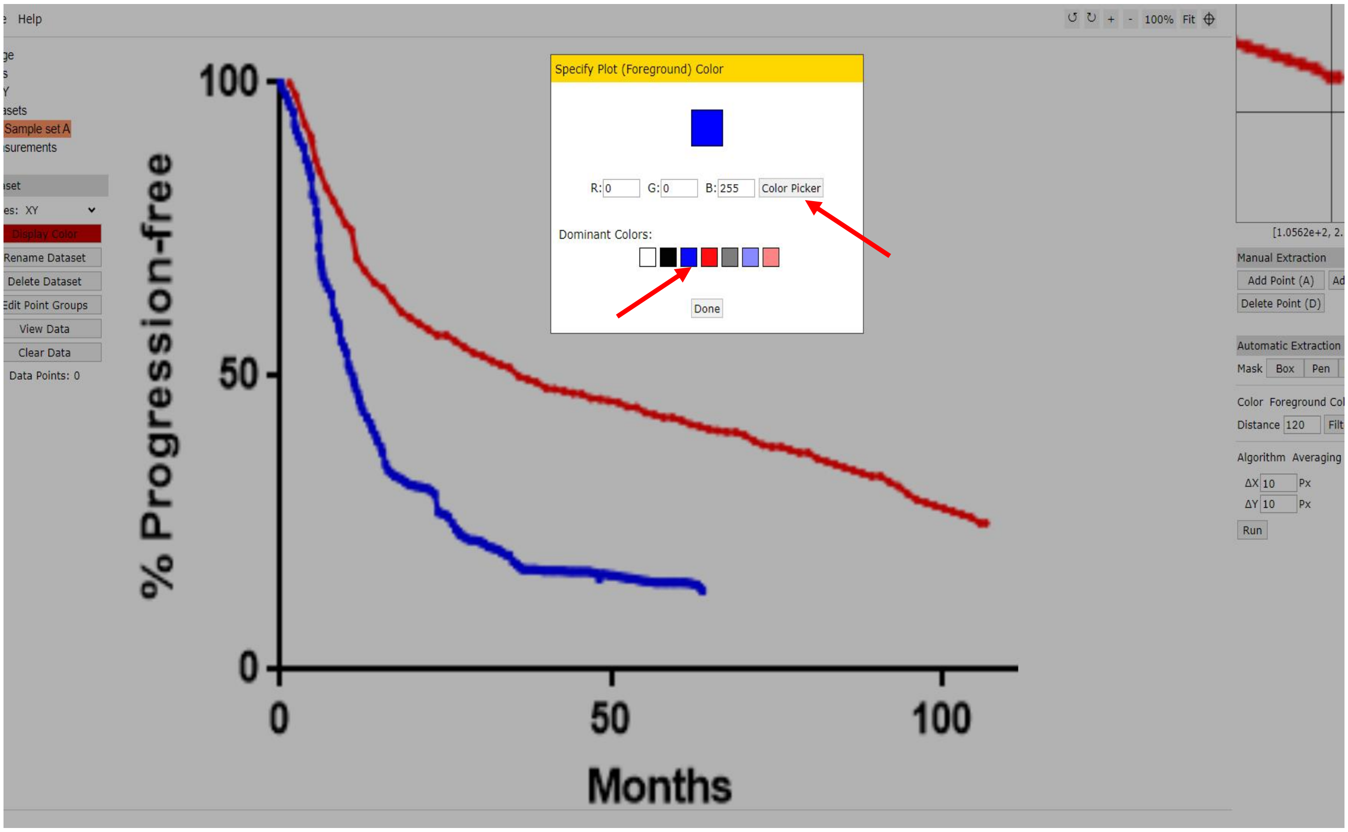


18. If you select “Color Picker”, put the cursor directly over the curve of interest (in this case, the blue curve) and hit enter. The color of interest will then appear in the upper box (although it was already there in this particular example since it is the program default color). Then hit “Done”.

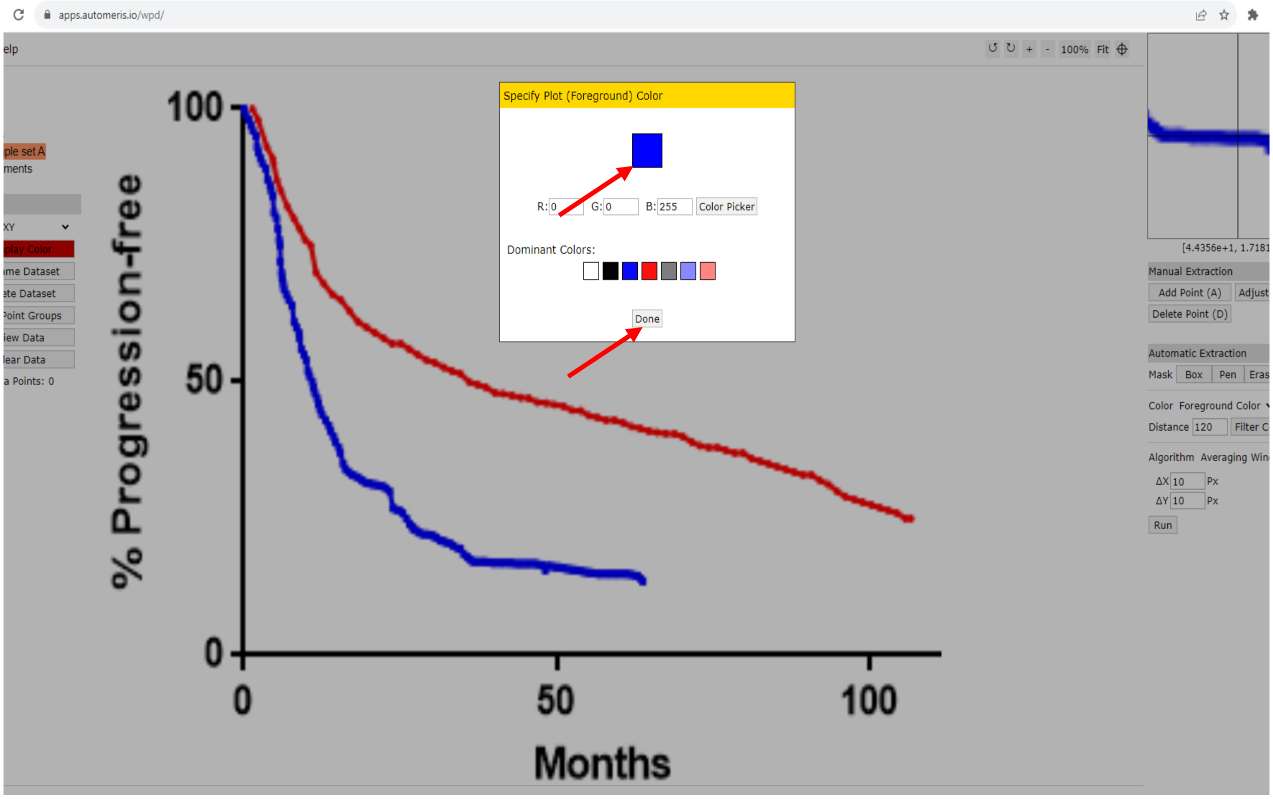


19. Then click on either “Pen” (which is most useful if different curves have similar colors) or “Box” (which can be used if there are no other factors on the graph that are similar to the color that you picked).

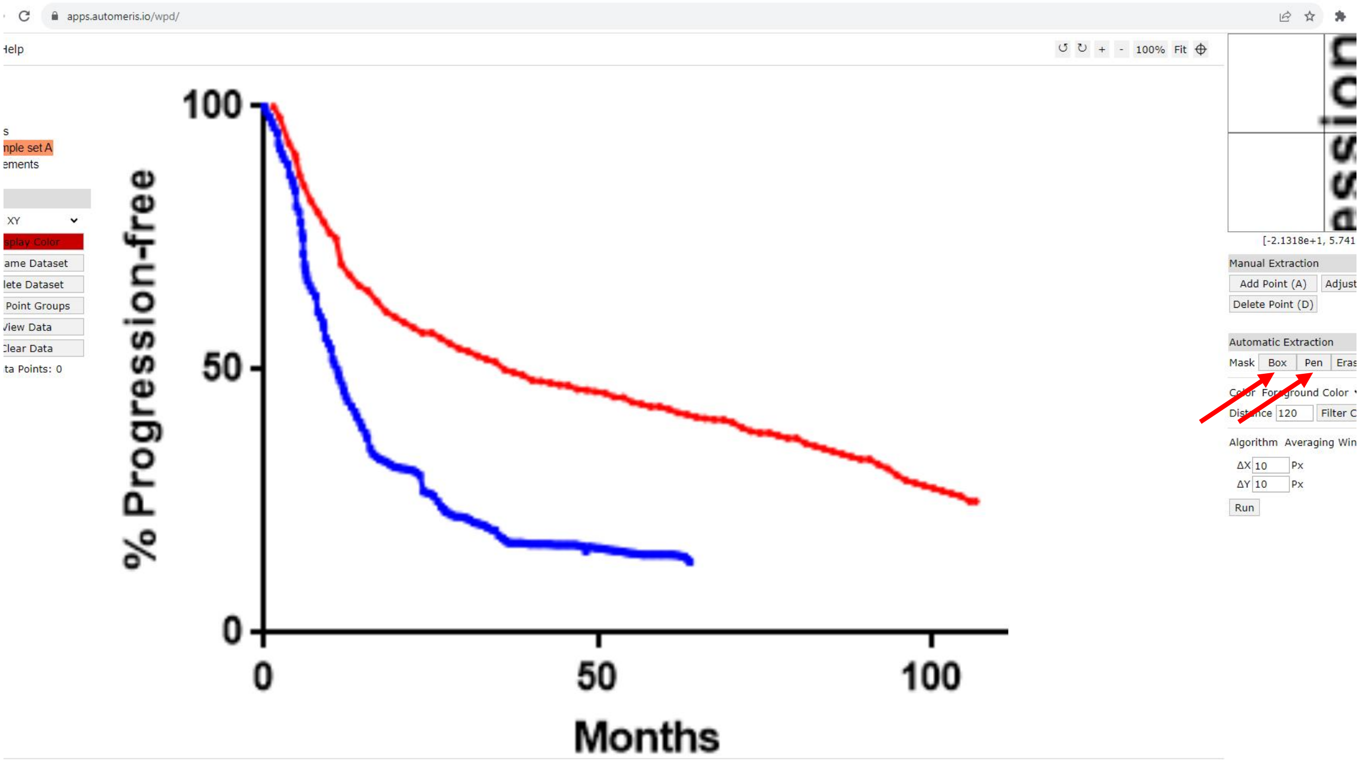


20. In this case we selected “Pen”. You then move the cursor down the curve of interest, and it colors it yellow.

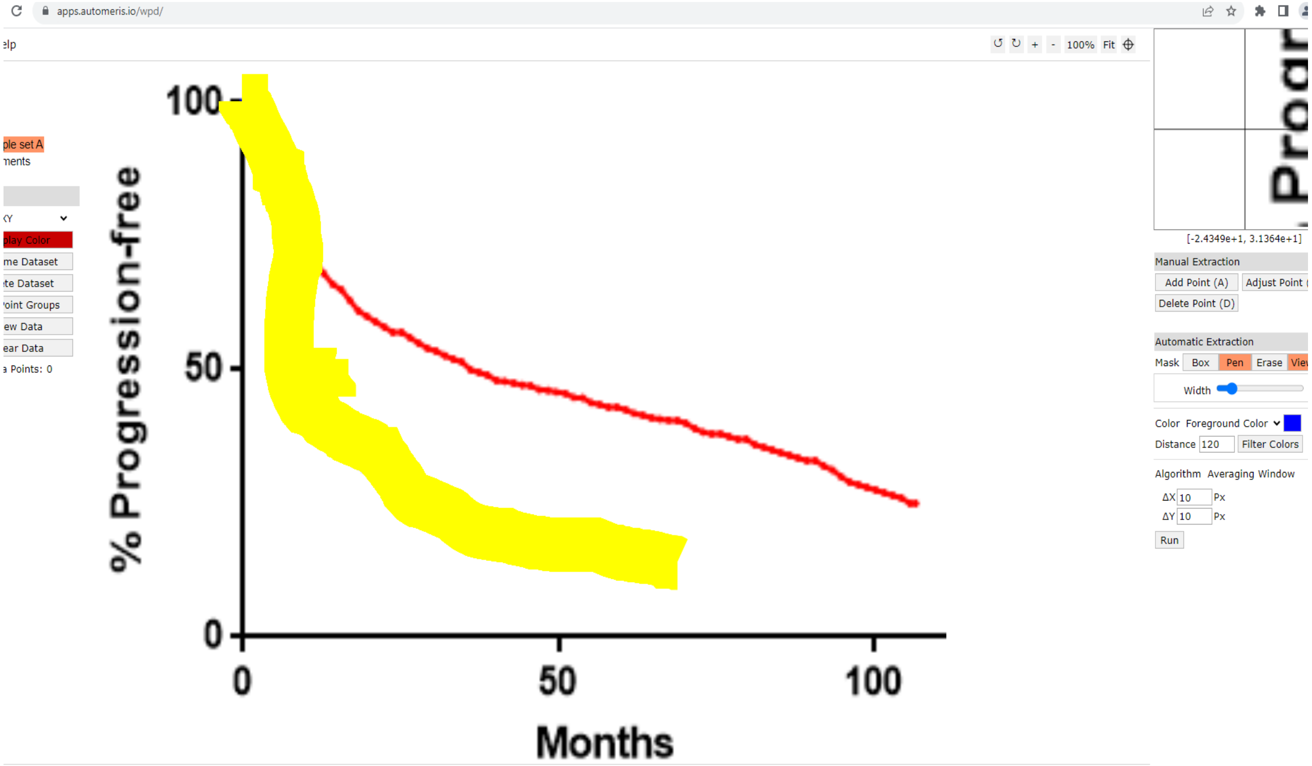


21. If we instead select “Box”, we can move the cursor over the entire area of interest, and it will select it all. This approach is a bit more efficient but can result in erroneous entries if there are other structures similar in color to the curve of interest.

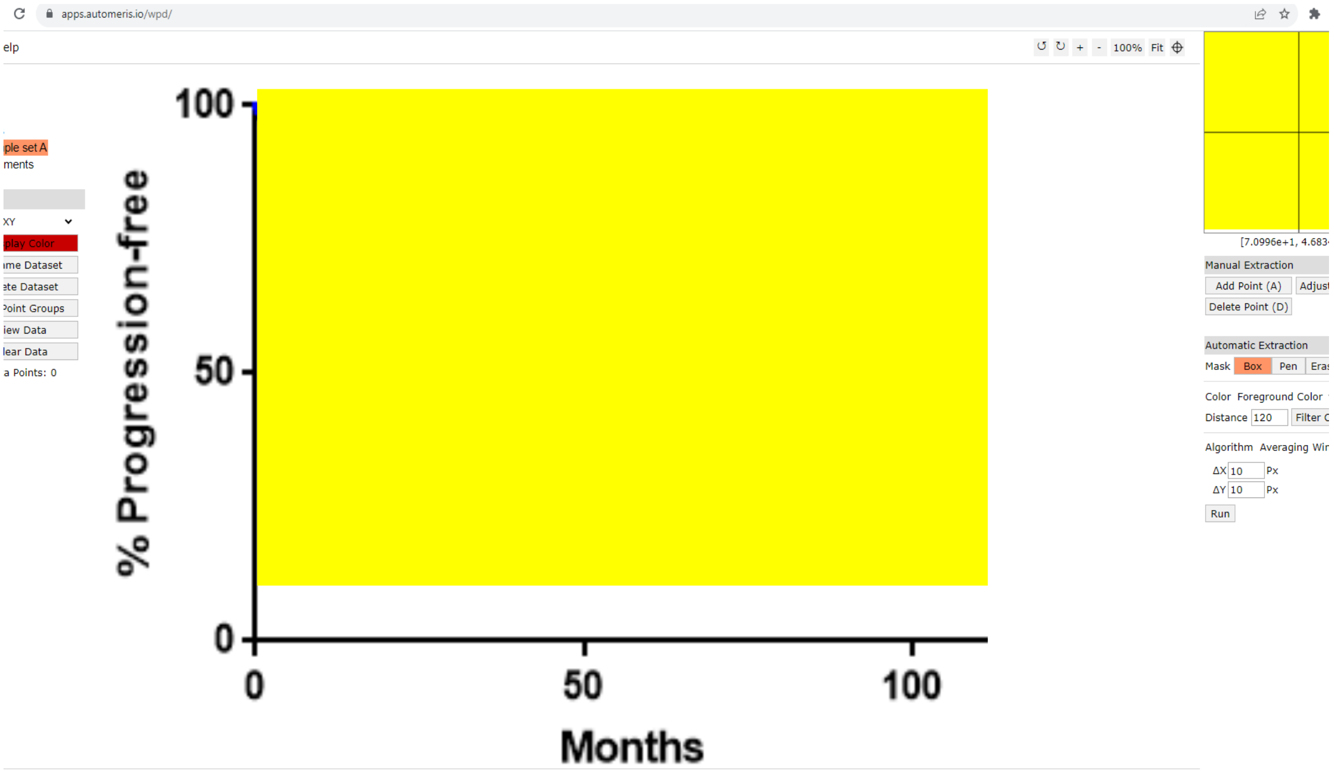

22. We could then elect to adjust the pixel size. The default setting is 10. Setting it smaller means data points will be closer together and setting it larger means they will be further apart. Then hit "Run" and the program will put red dots along the selected curve (in this case, the blue curve).

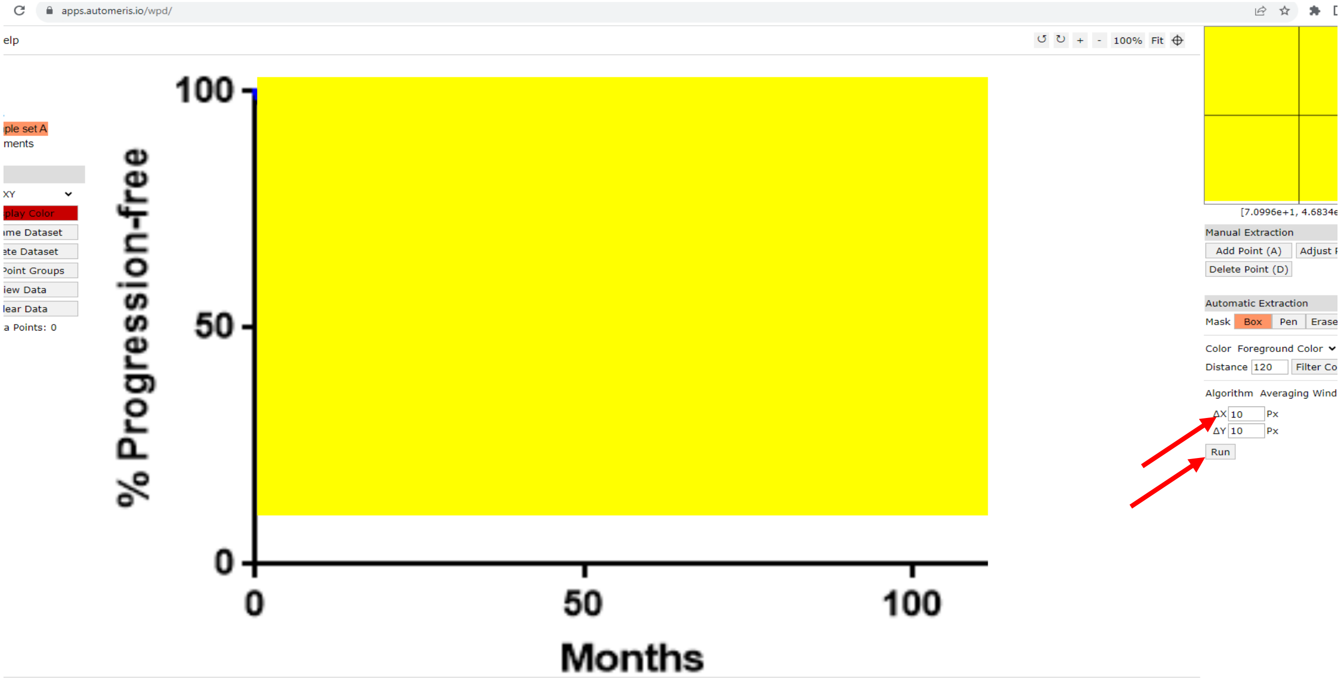

23. The data points appear as new red points along the curve. In this case, 4 points were added erroneously to the black y axis, and no points were added to the upper part of the blue curve.

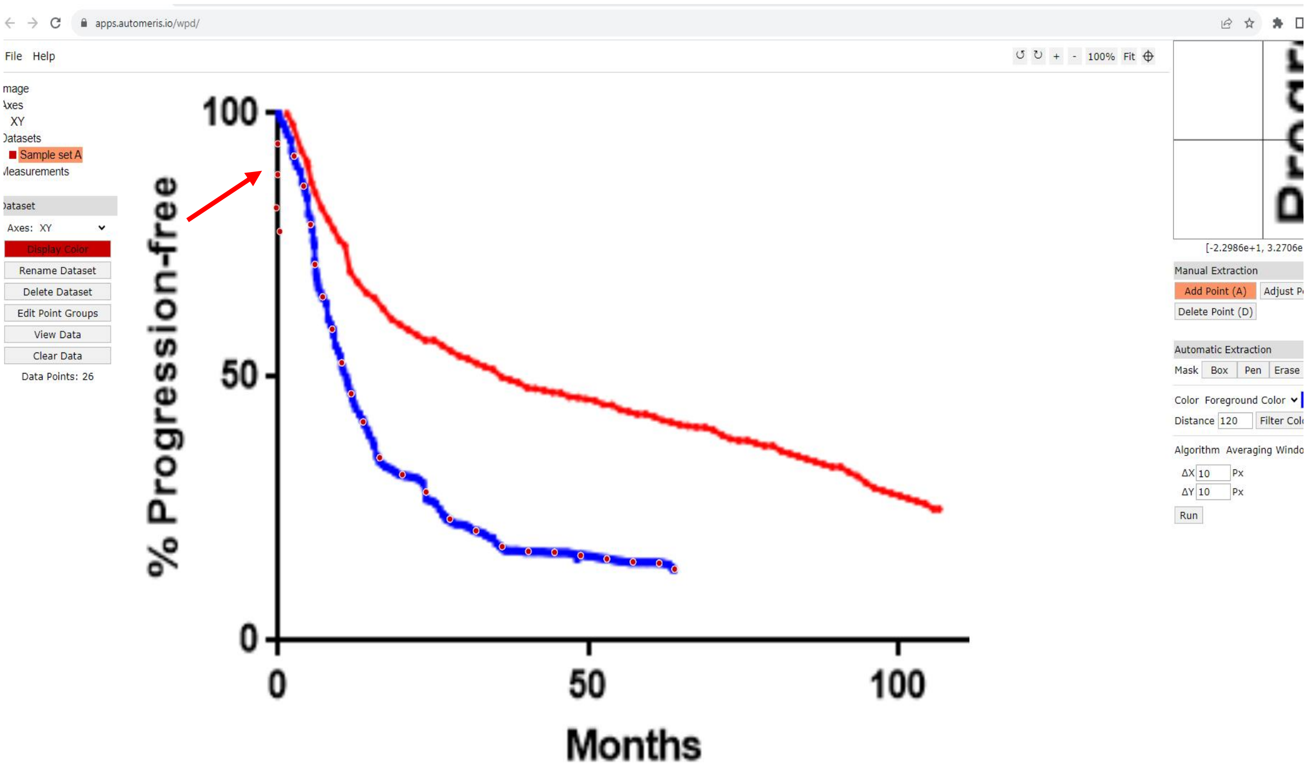


24. To remove the erroneous points, click on “Delete Point”, then place the cursor near the erroneous point and click enter. Here, the extra points have now been removed.

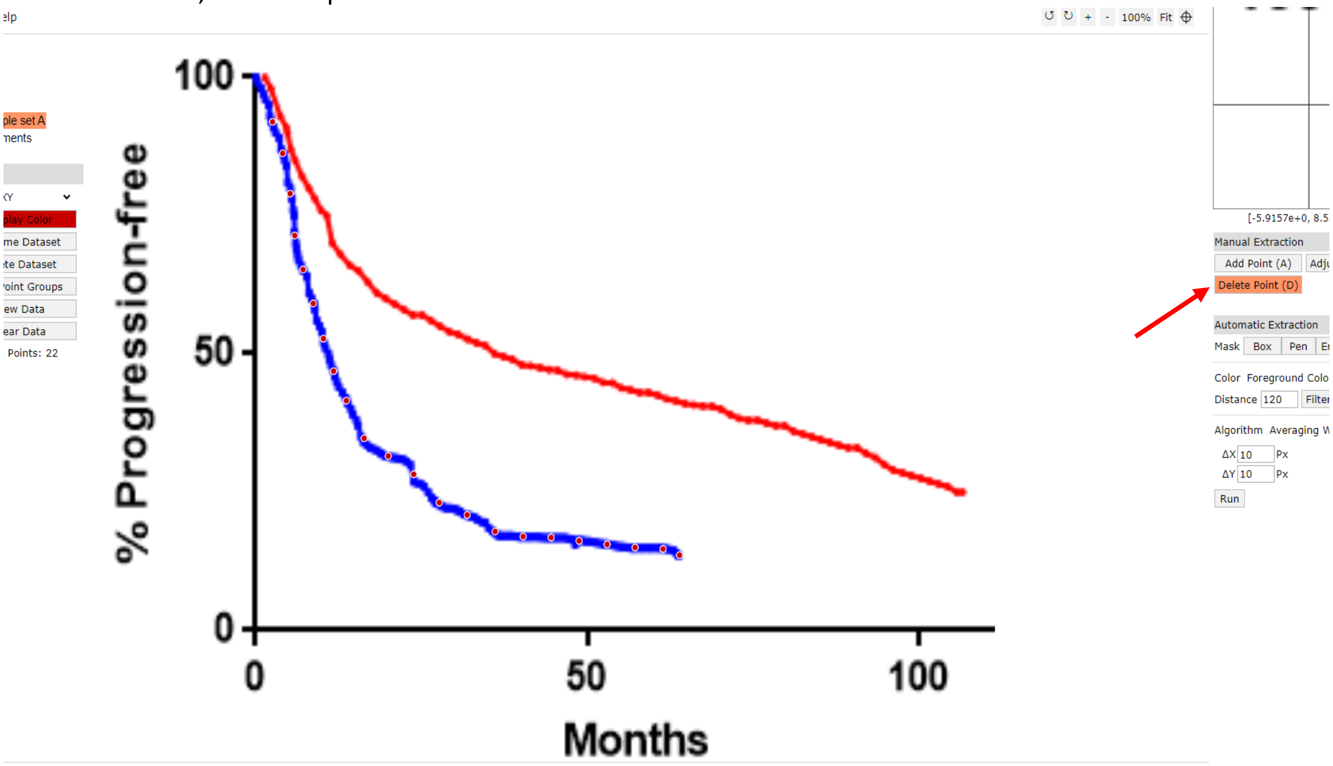


25. To add missing points, click on “Add Point”. Then place the cursor where you want a point added and click enter. We have now added 3 points to the upper part of the blue curve.

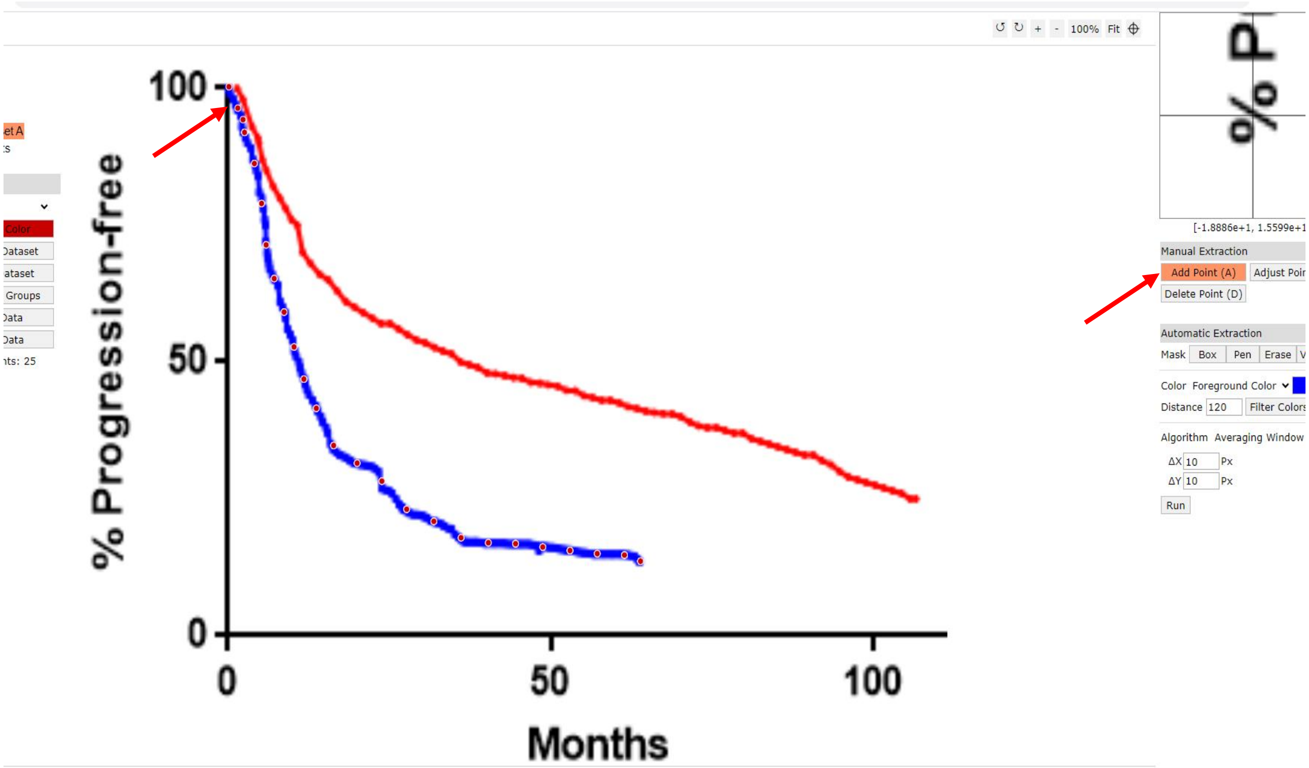


26. Then hit "View Data".

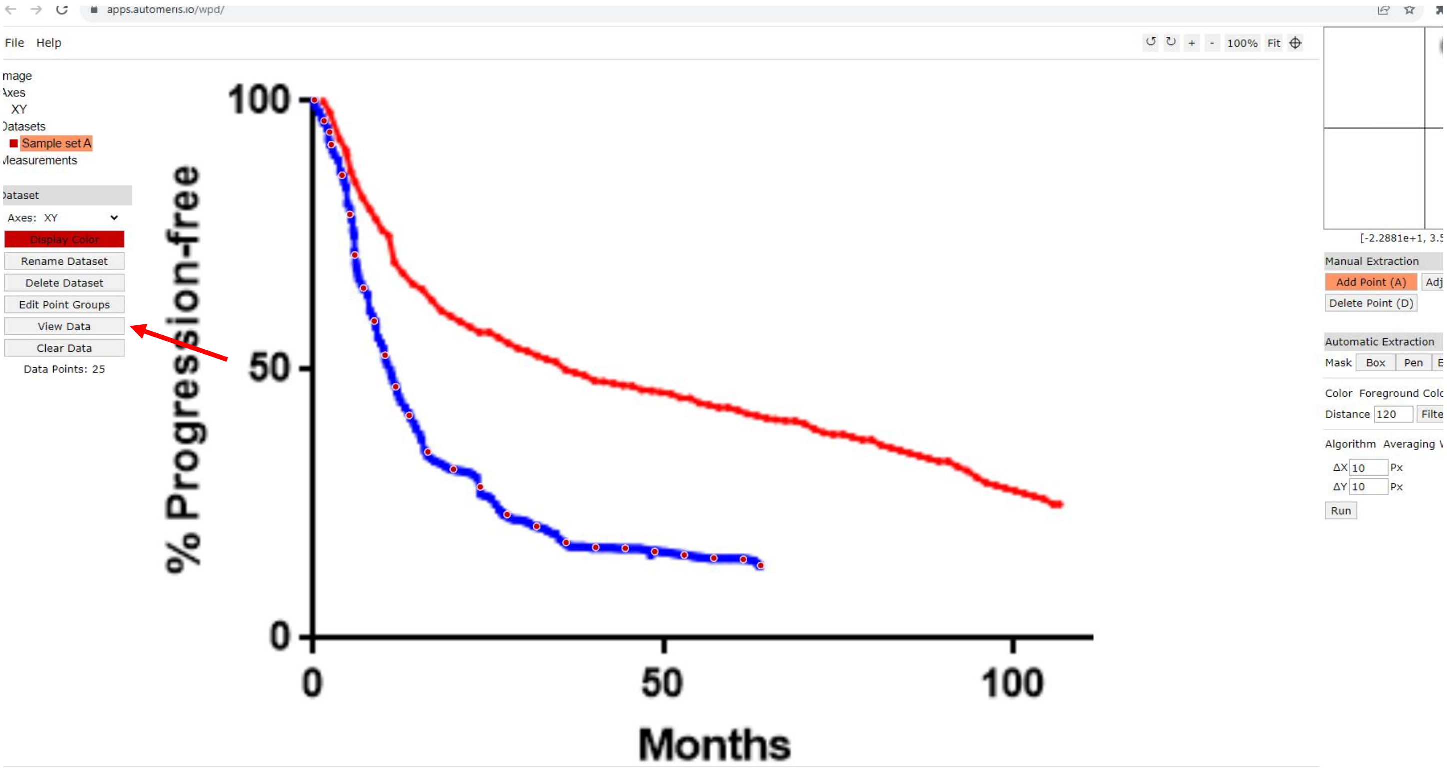


27. After hitting "View Data", the data co-ordinates ae displayed. Once they are displayed, hit "Download .CSV".

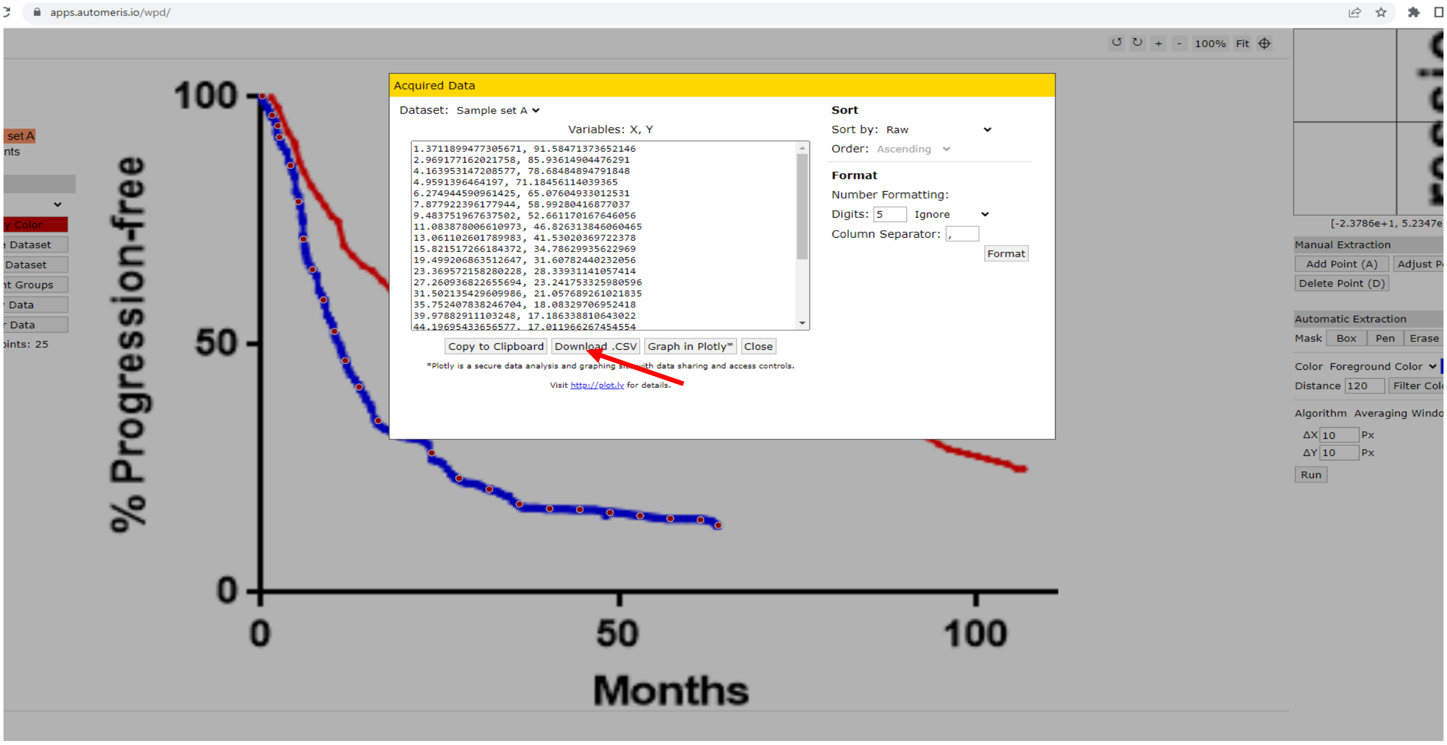


28. The downloaded .CSV data are displayed below. They can then be saved as an Excel file, and the data can be sorted by column A (time in months). We correct to 0 any time points that may have been reported as being slightly less than 0, and we correct to 100% any survival values that may have been reported as being slightly above 100%. These data can now be used with GraphPad Prism.

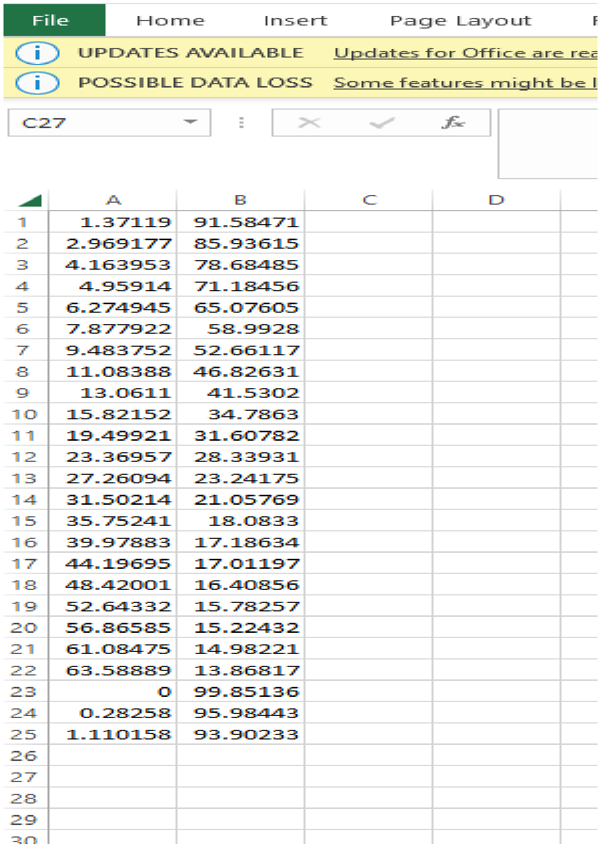


| | A | B | C | D |
|---|---|---|---|---|
| 1 | 1.37119 | 91.58471 | | |
| 2 | 2.969177 | 85.93615 | | |
| 3 | 4.163953 | 78.68485 | | |
| 4 | 4.95914 | 71.18456 | | |
| 5 | 6.274945 | 65.07605 | | |
| 6 | 7.877922 | 58.9928 | | |
| 7 | 9.483752 | 52.66117 | | |
| 8 | 11.08388 | 46.82631 | | |
| 9 | 13.0611 | 41.5302 | | |
| 10 | 15.82152 | 34.7863 | | |
| 11 | 19.49921 | 31.60782 | | |
| 12 | 23.36957 | 28.33931 | | |
| 13 | 27.26094 | 23.24175 | | |
| 14 | 31.50214 | 21.05769 | | |
| 15 | 35.75241 | 18.0833 | | |
| 16 | 39.97883 | 17.18634 | | |
| 17 | 44.19695 | 17.01197 | | |
| 18 | 48.42001 | 16.40856 | | |
| 19 | 52.64332 | 15.78257 | | |
| 20 | 56.86585 | 15.22432 | | |
| 21 | 61.08475 | 14.98221 | | |
| 22 | 63.58889 | 13.86817 | | |
| 23 | 0 | 99.85136 | | |
| 24 | 0.28258 | 95.98443 | | |
| 25 | 1.110158 | 93.90233 | | |
| 26 | | | | |
| 27 | | | | |
| 28 | | | | |
| 29 | | | | |

29. Once data for Sample Set A have been saved, the data set can be closed. When "Clear Data" is clicked, the data for Sample Set A are cleared. The Dataset can then be renamed (eg, Sample Set B), the color of the other curve (red) can be selected using the Color Picker, and the process can be repeated to obtain the data co-ordinates for the red curve. Once familiar with the program, it typically takes 10-20 minutes to digitize a curve.

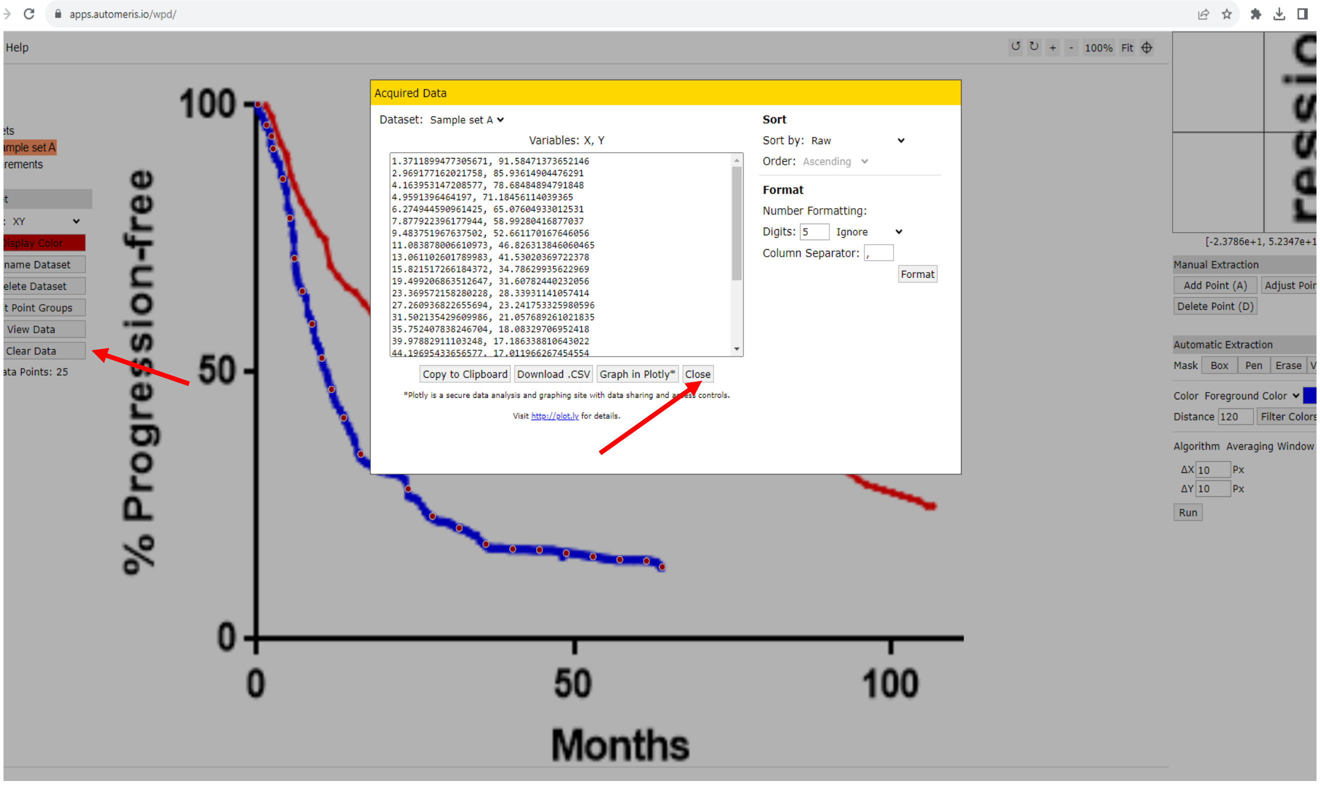

**How to replot data on a log-linear curve and perform exponential decay nonlinear regression analysis:**

1. We typically use GraphPad Prism Version 7 for these analyses, although other nonlinear regression analysis programs could also potentially be used. We have not compared other programs to GraphPad Prism.

2. After GraphPad is opened, click on “XY” in the left-hand column, and then click on “Y: enter and plot a single Y value for each point”, then on “Create”.

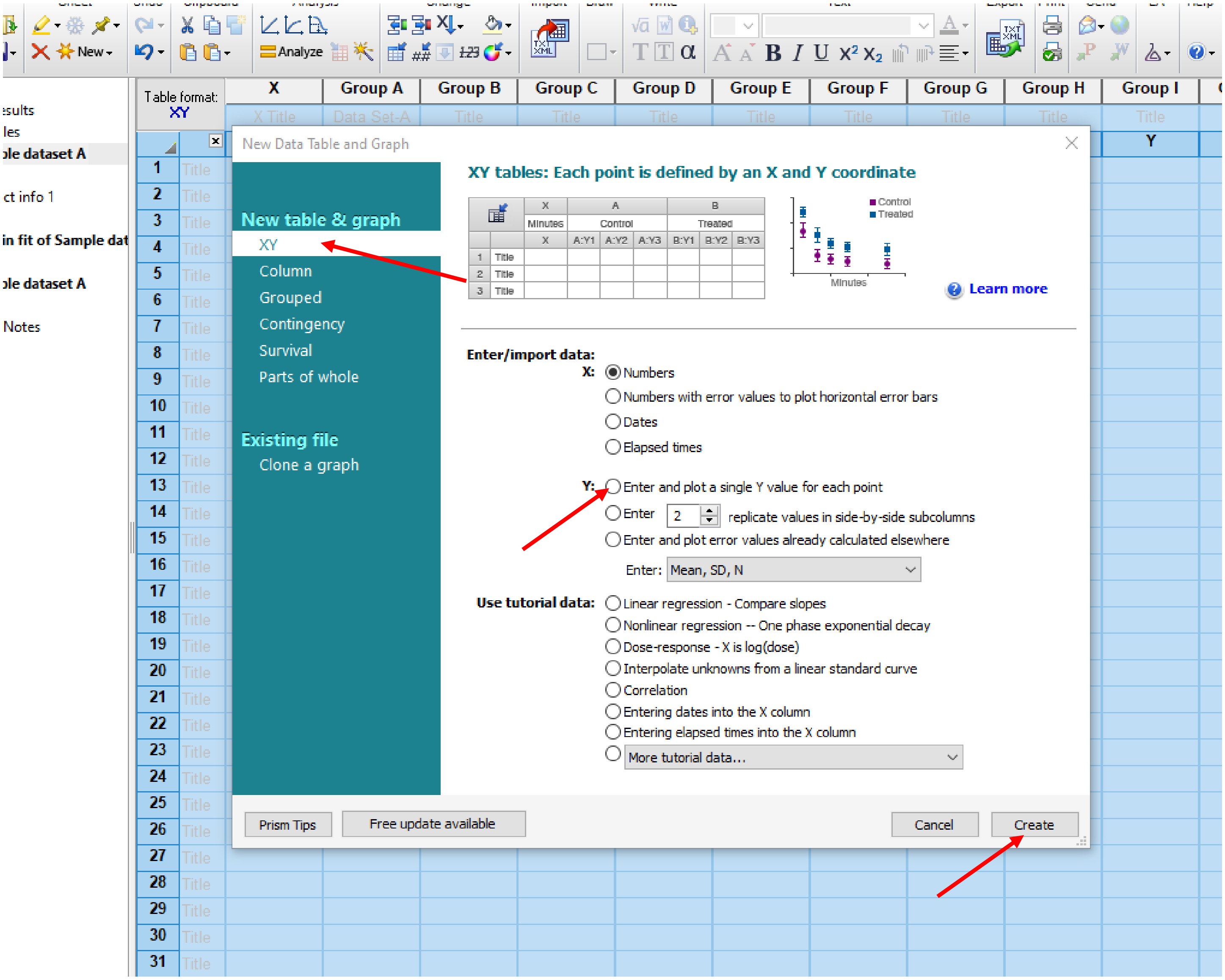

3. Enter the digitized survival data in GraphPad. The “x” column is time in months and the “y” column is proportion of patients who are event-free (eg, alive) at that time. Click on “Data 1” to name the data set, then click on “Analyze” to begin the analysis.

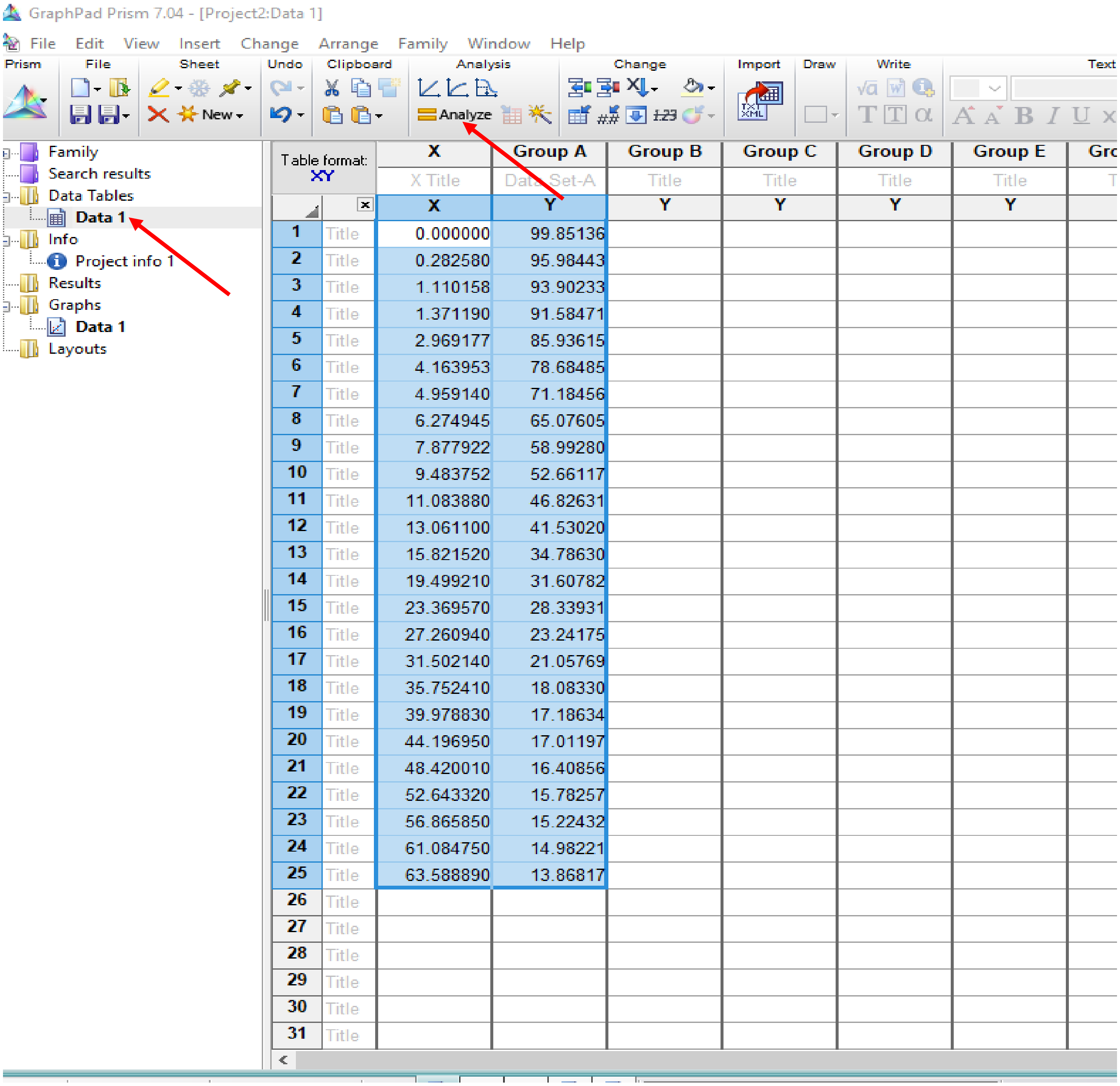


| | Title | X | Group A (Data Set-A) Y |
|---|---|---|---|
| 1 | Title | 0.000000 | 99.85136 |
| 2 | Title | 0.282580 | 95.98443 |
| 3 | Title | 1.110158 | 93.90233 |
| 4 | Title | 1.371190 | 91.58471 |
| 5 | Title | 2.969177 | 85.93615 |
| 6 | Title | 4.163953 | 78.68485 |
| 7 | Title | 4.959140 | 71.18456 |
| 8 | Title | 6.274945 | 65.07605 |
| 9 | Title | 7.877922 | 58.99280 |
| 10 | Title | 9.483752 | 52.66117 |
| 11 | Title | 11.083880 | 46.82631 |
| 12 | Title | 13.061100 | 41.53020 |
| 13 | Title | 15.821520 | 34.78630 |
| 14 | Title | 19.499210 | 31.60782 |
| 15 | Title | 23.369570 | 28.33931 |
| 16 | Title | 27.260940 | 23.24175 |
| 17 | Title | 31.502140 | 21.05769 |
| 18 | Title | 35.752410 | 18.08330 |
| 19 | Title | 39.978830 | 17.18634 |
| 20 | Title | 44.196950 | 17.01197 |
| 21 | Title | 48.420010 | 16.40856 |
| 22 | Title | 52.643320 | 15.78257 |
| 23 | Title | 56.865850 | 15.22432 |
| 24 | Title | 61.084750 | 14.98221 |
| 25 | Title | 63.588890 | 13.86817 |

4. We have named the dataset “Sample dataset A”. Next, click on “Nonlinear regression” and then on “OK”.

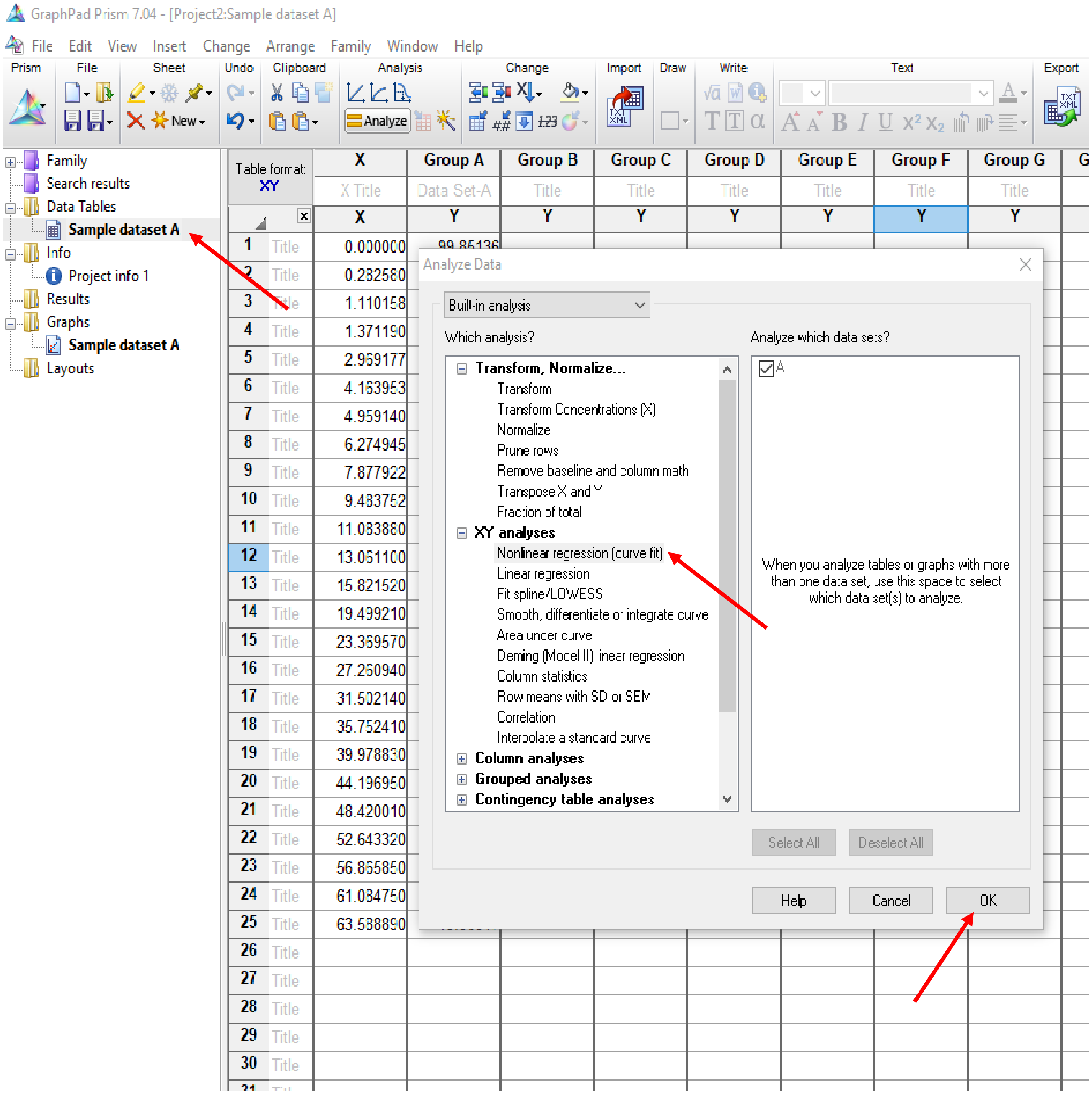

5. Click on "One phase decay" and then on "Constrain".

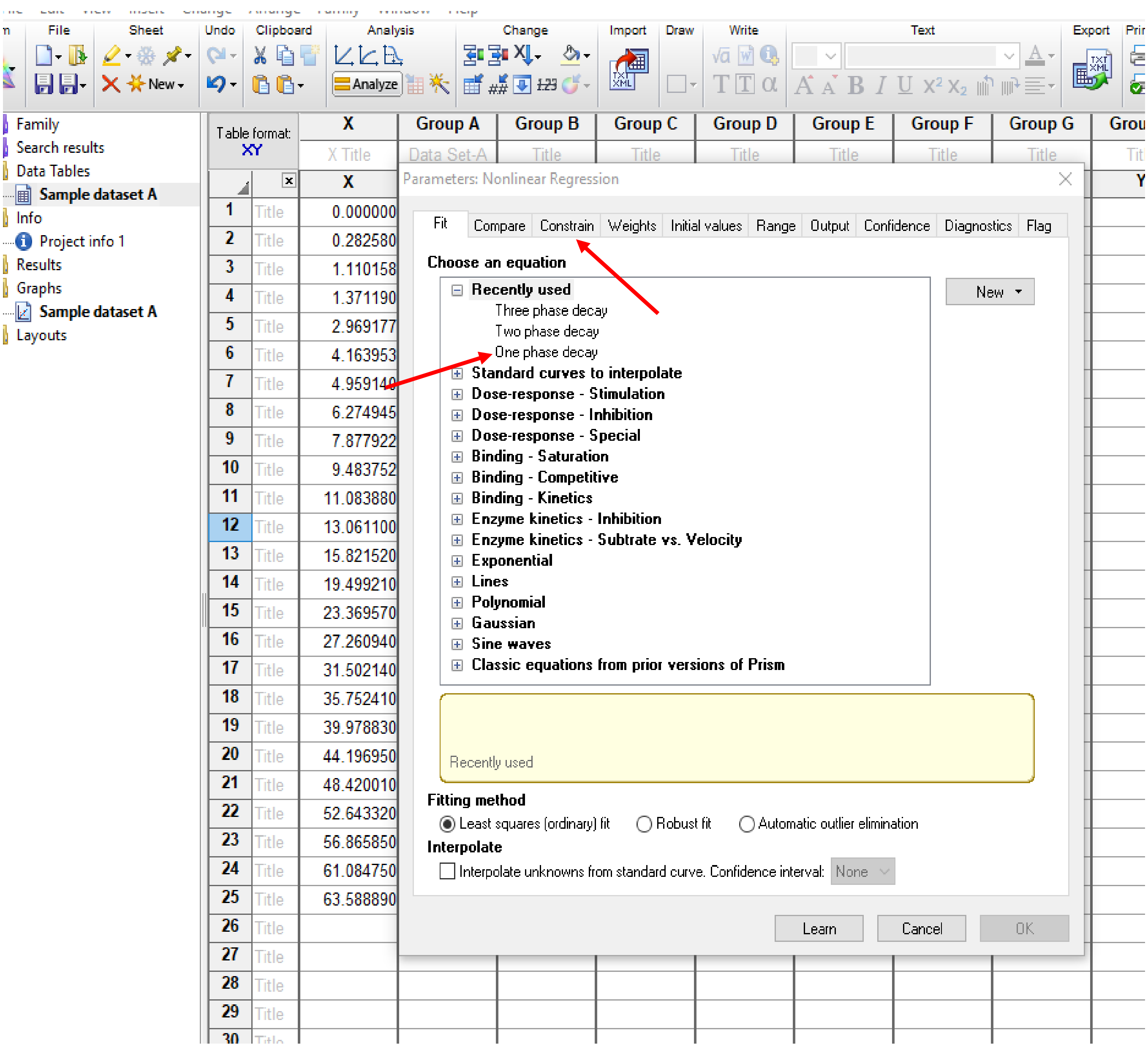

6. For Y0, change constraints from “No constraint” to “constraint equal to 100” and for Plateau, change constraints from “No constraint” to “constraint equal to 0”, then hit “OK”.

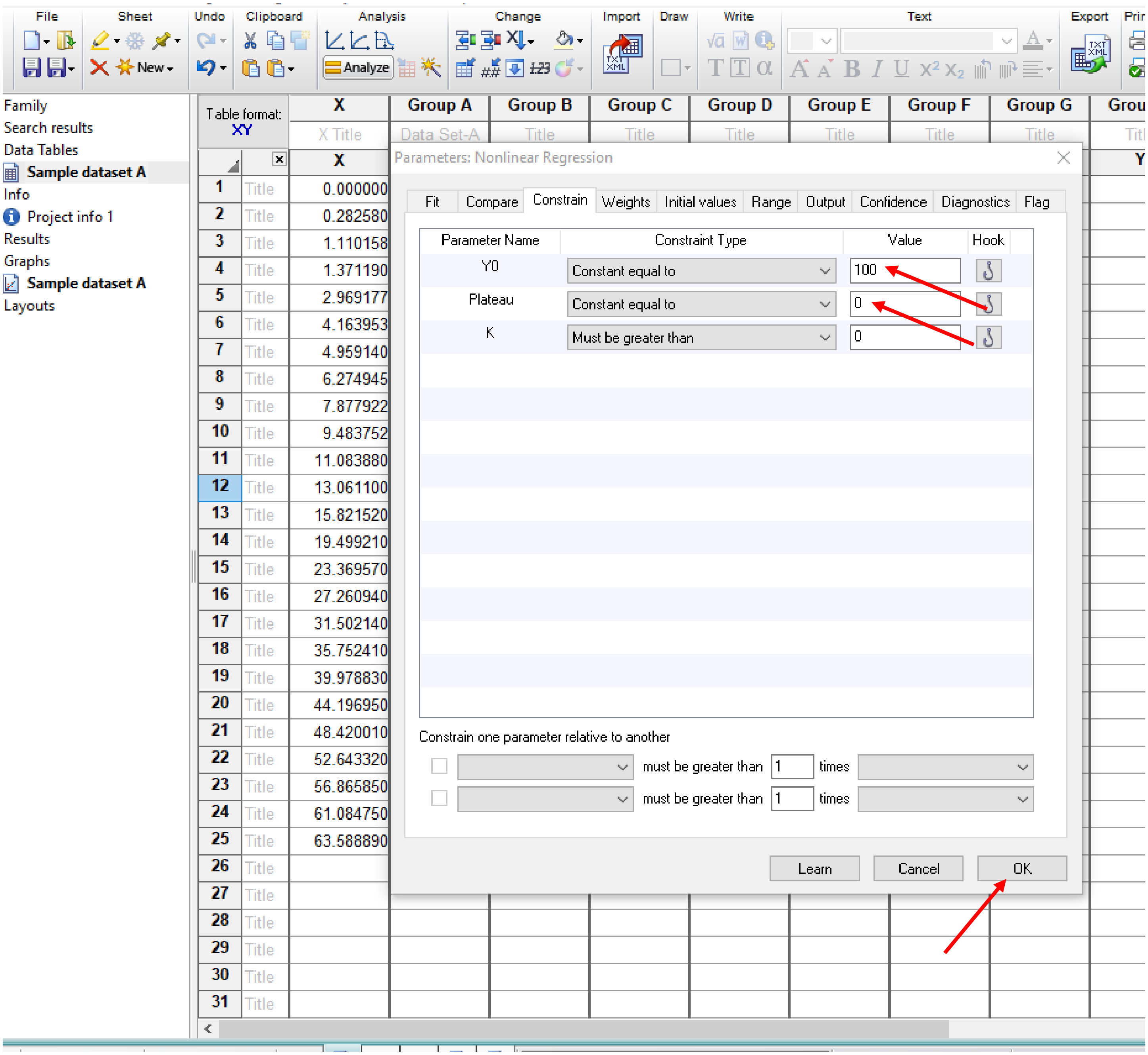

7. The analysis indicates that the half-life is 12.44 months, with 95% confidence intervals of 10.98 to 14.05 months, and $R^2$ 0.9556.

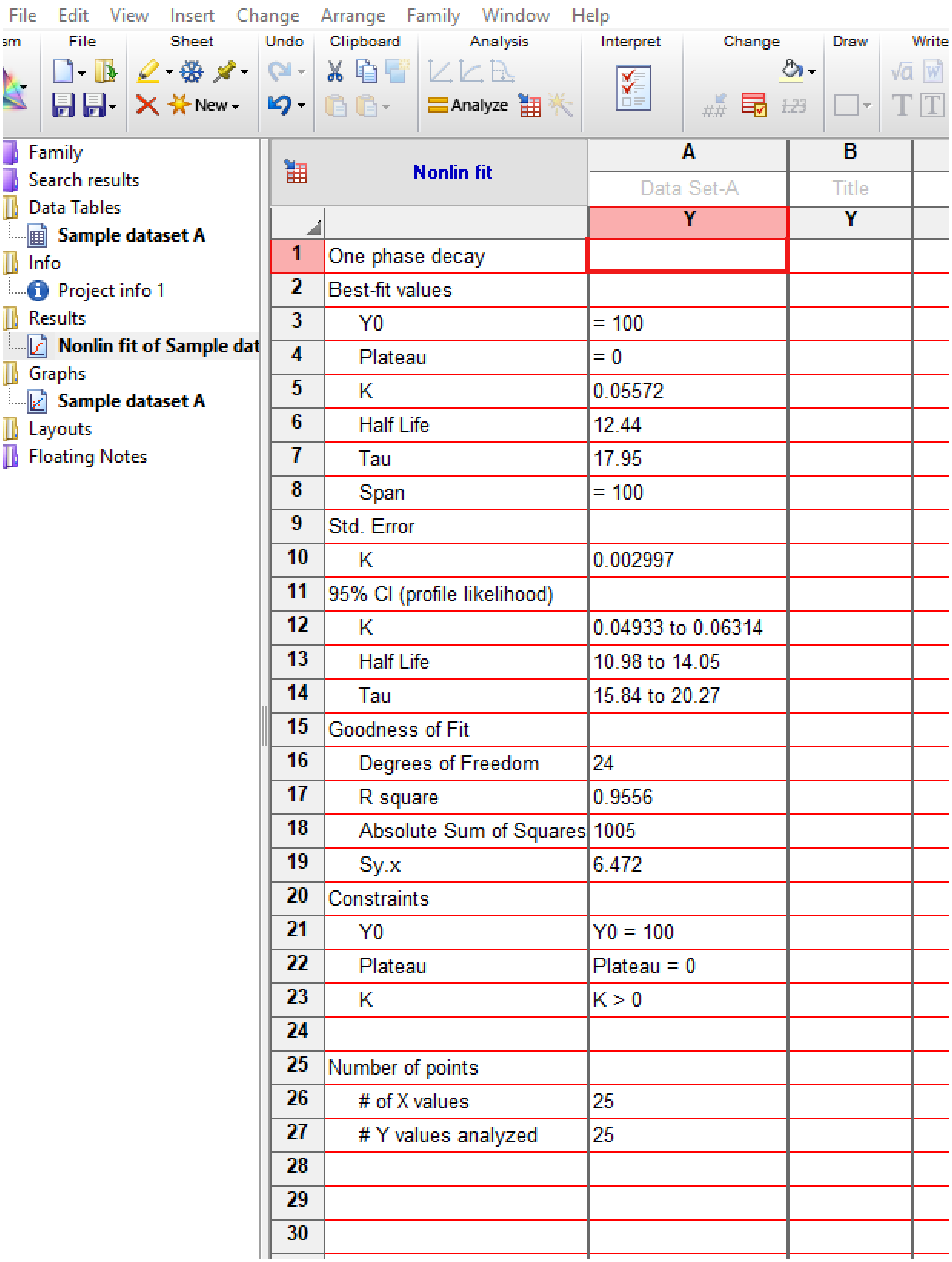


| | Nonlin fit | A<br>Data Set-A<br>Y | B<br>Title<br>Y |
|---|---|---|---|
| 1 | One phase decay | | |
| 2 | Best-fit values | | |
| 3 | Y0 | = 100 | |
| 4 | Plateau | = 0 | |
| 5 | K | 0.05572 | |
| 6 | Half Life | 12.44 | |
| 7 | Tau | 17.95 | |
| 8 | Span | = 100 | |
| 9 | Std. Error | | |
| 10 | K | 0.002997 | |
| 11 | 95% CI (profile likelihood) | | |
| 12 | K | 0.04933 to 0.06314 | |
| 13 | Half Life | 10.98 to 14.05 | |
| 14 | Tau | 15.84 to 20.27 | |
| 15 | Goodness of Fit | | |
| 16 | Degrees of Freedom | 24 | |
| 17 | R square | 0.9556 | |
| 18 | Absolute Sum of Squares | 1005 | |
| 19 | Sy.x | 6.472 | |
| 20 | Constraints | | |
| 21 | Y0 | Y0 = 100 | |
| 22 | Plateau | Plateau = 0 | |
| 23 | K | K > 0 | |
| 24 | | | |
| 25 | Number of points | | |
| 26 | # of X values | 25 | |
| 27 | # Y values analyzed | 25 | |
| 28 | | | |
| 29 | | | |
| 30 | | | |

8. These are the data plotted on a linear-linear scale. Click on “Change” on the top line to convert it to log-linear.

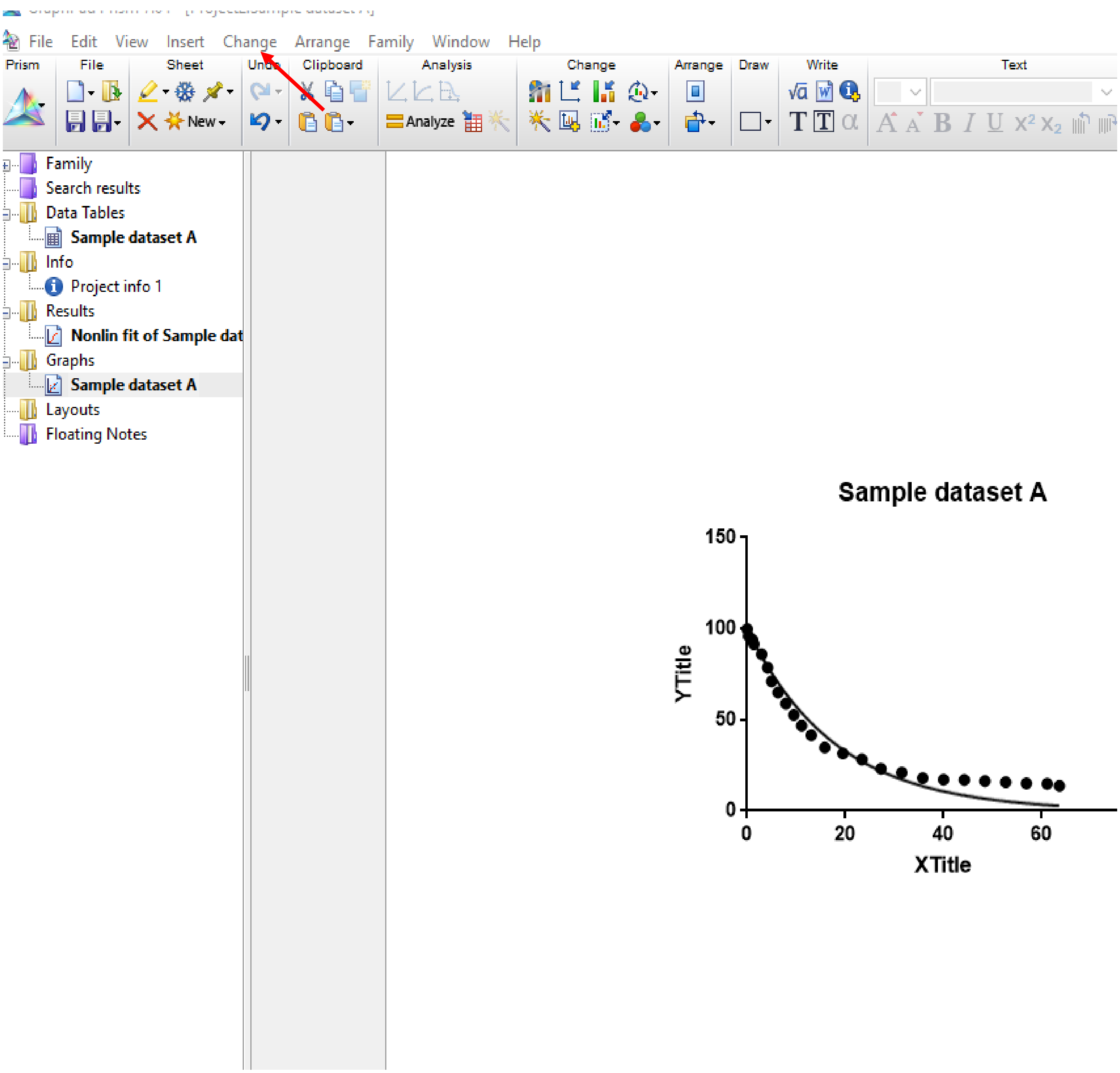

9. On the drop-down menu, click on “Y axis (left)”. From the pull-down menu for “Scale”, change “Linear” to “Log 10”. Click on “Automatically determine the range and interval” to turn it off. Then set the “Maximum” at 100. Then click “OK”.

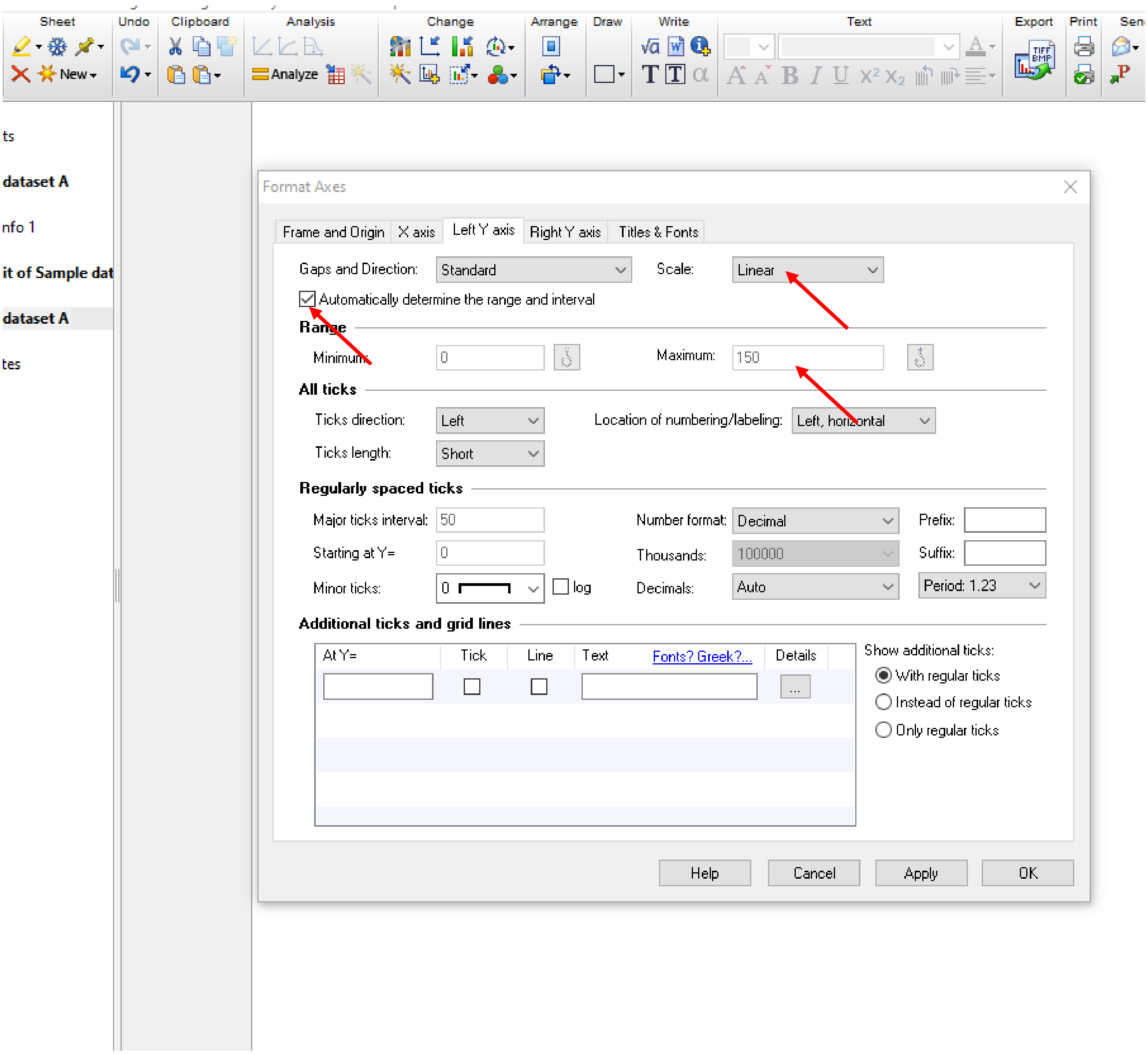

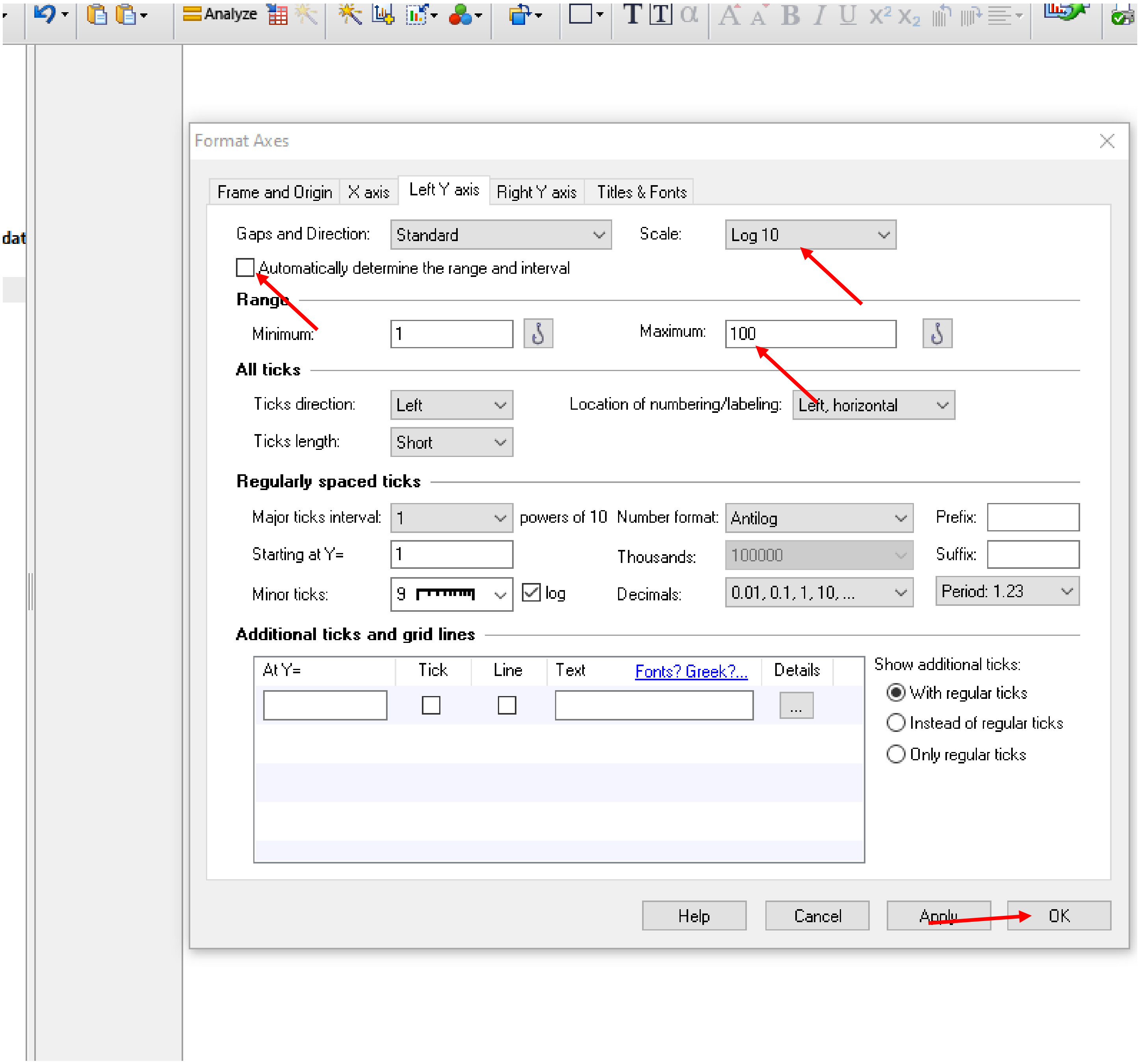
Analyze
Format Axes
Frame and Origin
X axis
Left Y axis
Right Y axis
Titles & Fonts
Gaps and Direction:
Standard
Scale:
Log 10
Automatically determine the range and interval
Range
Minimum:
1
Maximum:
100
All ticks
Ticks direction:
Left
Location of numbering/labeling:
Left, horizontal
Ticks length:
Short
Regularly spaced ticks
Major ticks interval:
1
powers of 10
Number format:
Antilog
Prefix:
Starting at Y=
1
Thousands:
100000
Suffix:
Minor ticks:
9
log
Decimals:
0.01, 0.1, 1, 10, ...
Period: 1.23
Additional ticks and grid lines
At Y=
Tick
Line
Text
Fonts? Greek?...
Details
...
Show additional ticks:
With regular ticks
Instead of regular ticks
Only regular ticks
Help
Cancel
Apply
OK

10. Below is the log-linear plot. The deviation of the log-linear plot to the right indicates that this curve is following 2-phase exponential decay. Next, click on “New” at the top to re-enter the same data to permit 2-phase decay EDNLRA.

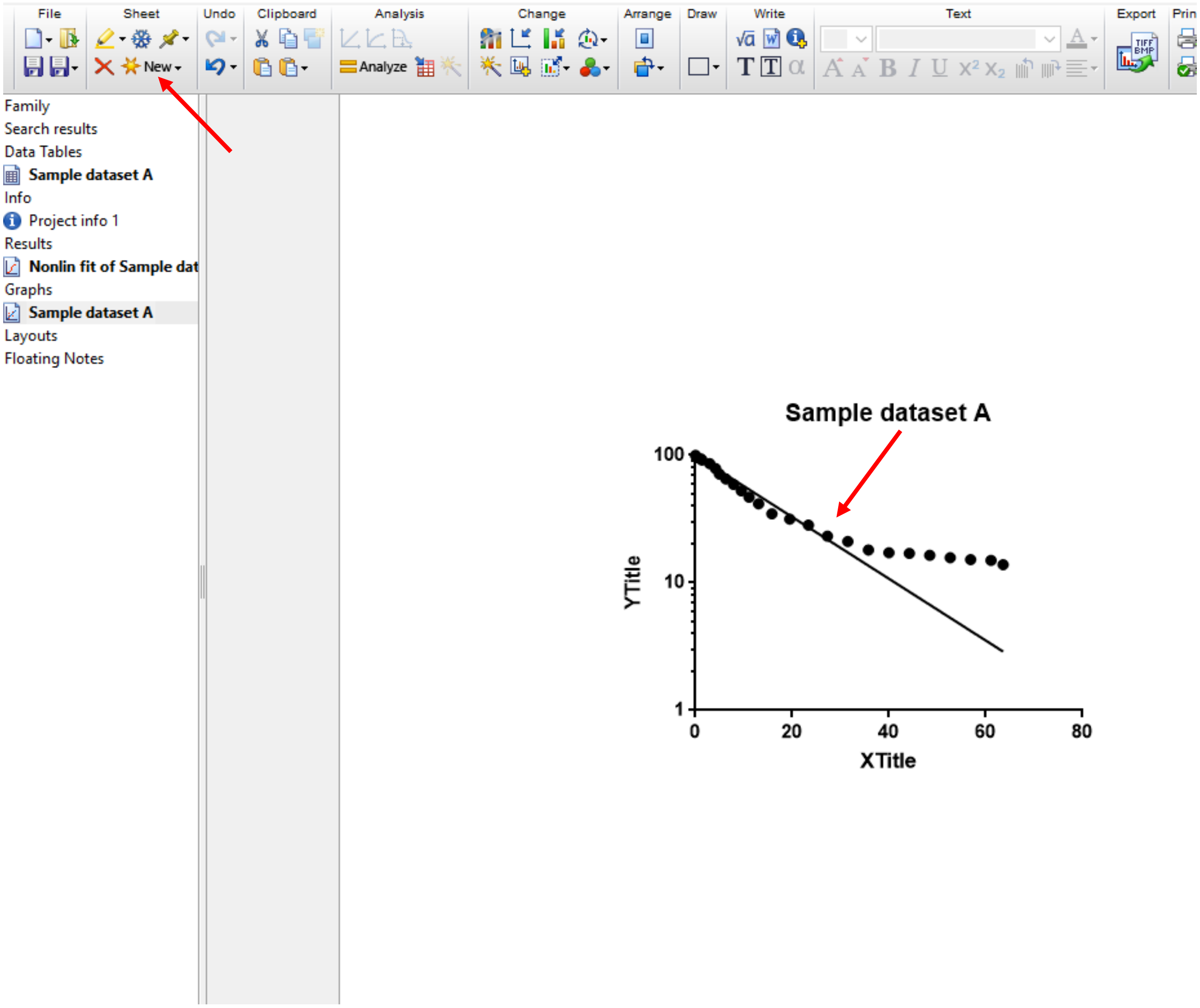

11. Again, click on “XY”, then on “Enter and plot a single Y value for each point”, then on “Create”.

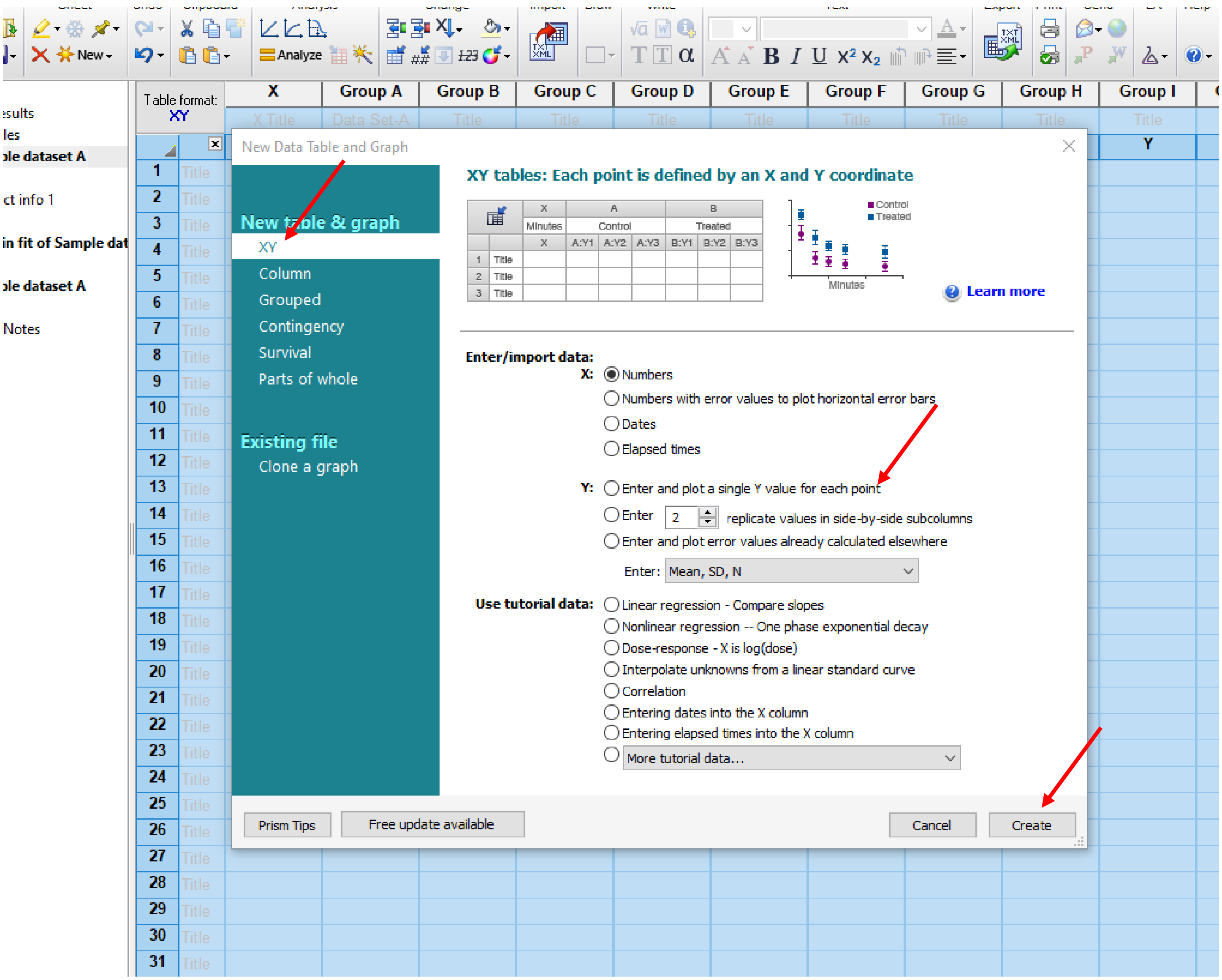

12. Enter the same data as for 1-phase decay, and rename the dataset indicating that it is for 2-phase decay analysis, then click on “Analyze”.

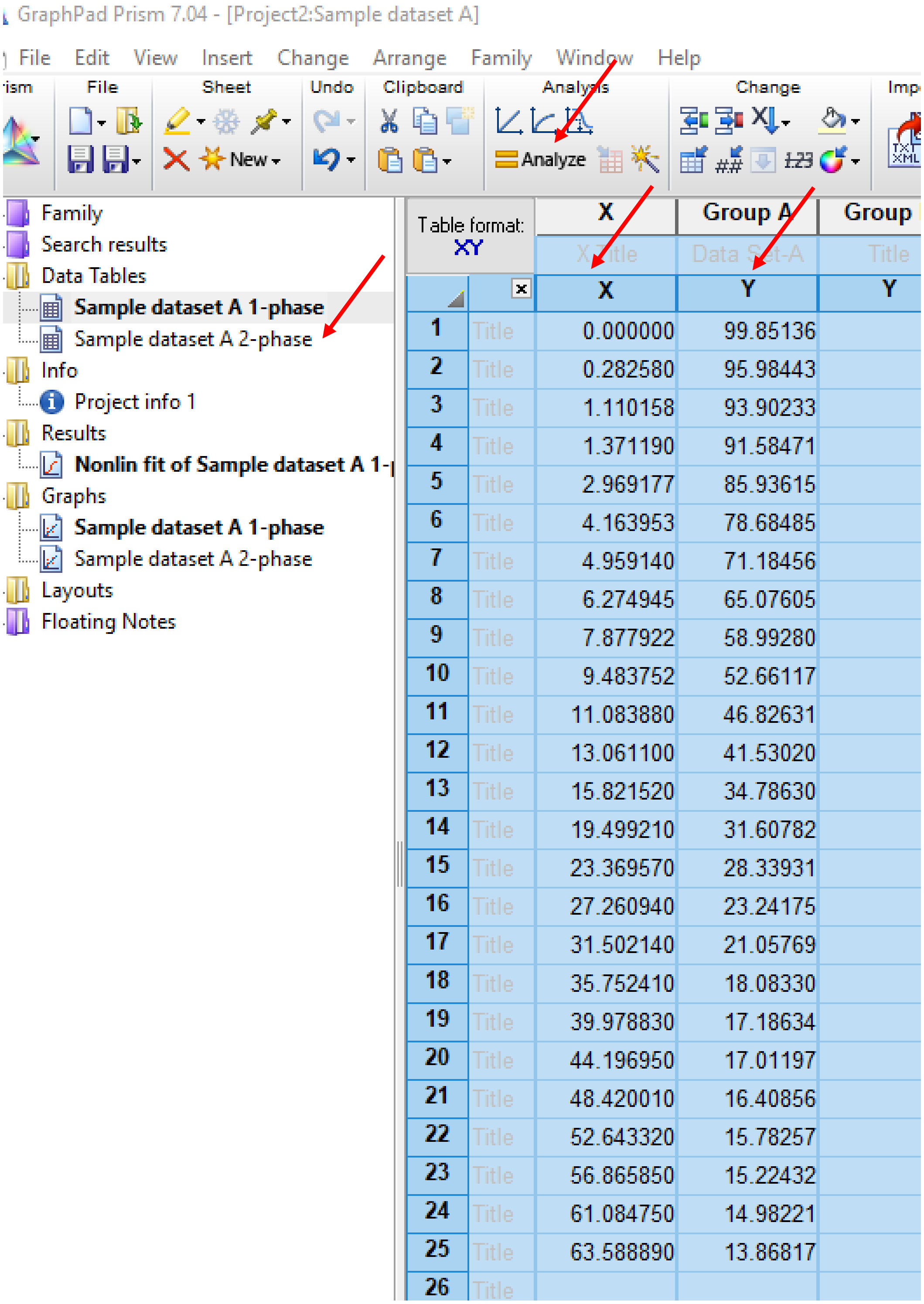


| | | X | Y |
|---|---|---|---|
| 1 | Title | 0.000000 | 99.85136 |
| 2 | Title | 0.282580 | 95.98443 |
| 3 | Title | 1.110158 | 93.90233 |
| 4 | Title | 1.371190 | 91.58471 |
| 5 | Title | 2.969177 | 85.93615 |
| 6 | Title | 4.163953 | 78.68485 |
| 7 | Title | 4.959140 | 71.18456 |
| 8 | Title | 6.274945 | 65.07605 |
| 9 | Title | 7.877922 | 58.99280 |
| 10 | Title | 9.483752 | 52.66117 |
| 11 | Title | 11.083880 | 46.82631 |
| 12 | Title | 13.061100 | 41.53020 |
| 13 | Title | 15.821520 | 34.78630 |
| 14 | Title | 19.499210 | 31.60782 |
| 15 | Title | 23.369570 | 28.33931 |
| 16 | Title | 27.260940 | 23.24175 |
| 17 | Title | 31.502140 | 21.05769 |
| 18 | Title | 35.752410 | 18.08330 |
| 19 | Title | 39.978830 | 17.18634 |
| 20 | Title | 44.196950 | 17.01197 |
| 21 | Title | 48.420010 | 16.40856 |
| 22 | Title | 52.643320 | 15.78257 |
| 23 | Title | 56.865850 | 15.22432 |
| 24 | Title | 61.084750 | 14.98221 |
| 25 | Title | 63.588890 | 13.86817 |
| 26 | Title | | |

13. Click on “Nonlinear regression”, then on “OK”.

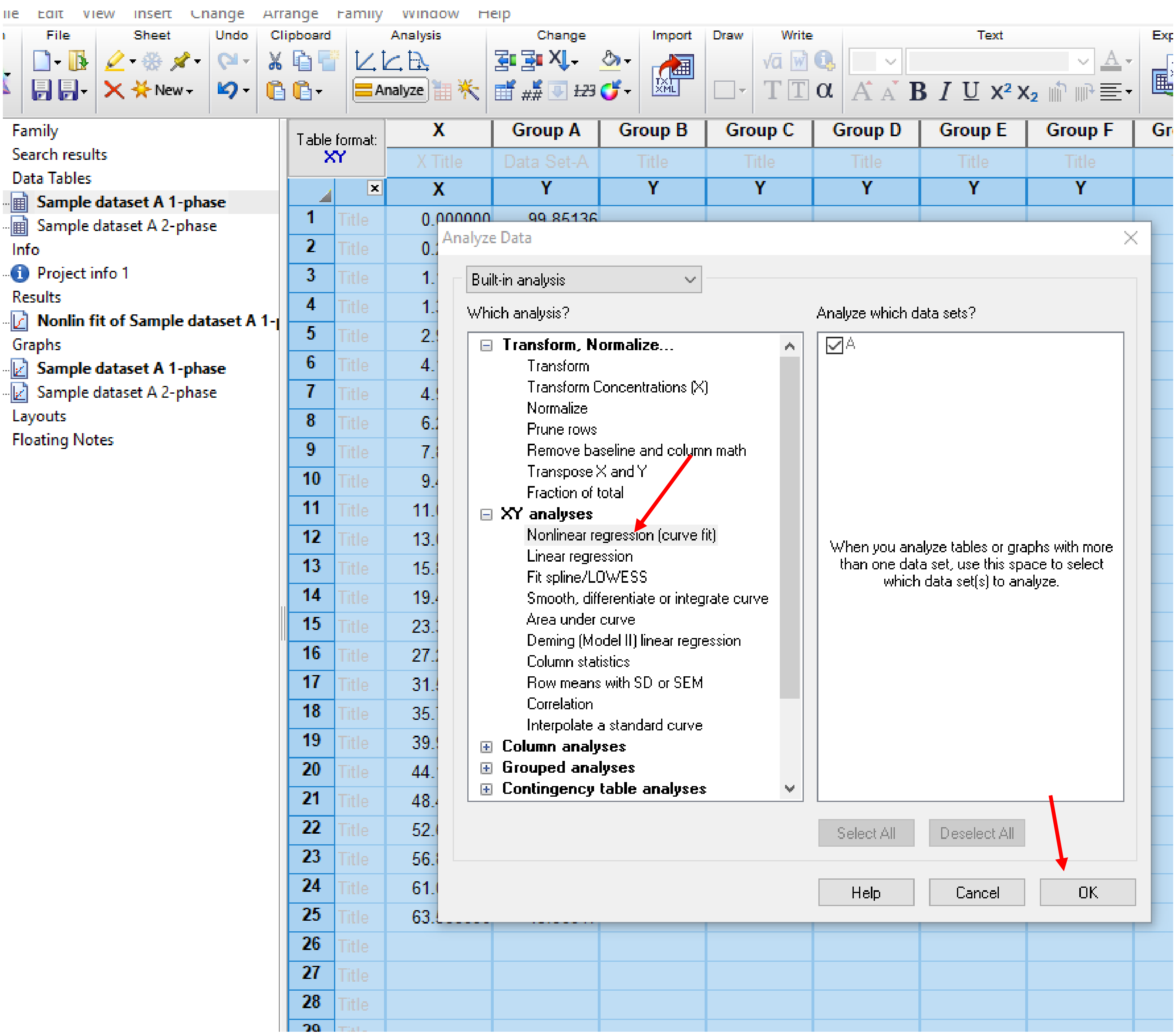

14. Click on “Two phase decay”, then on “Constrain”.

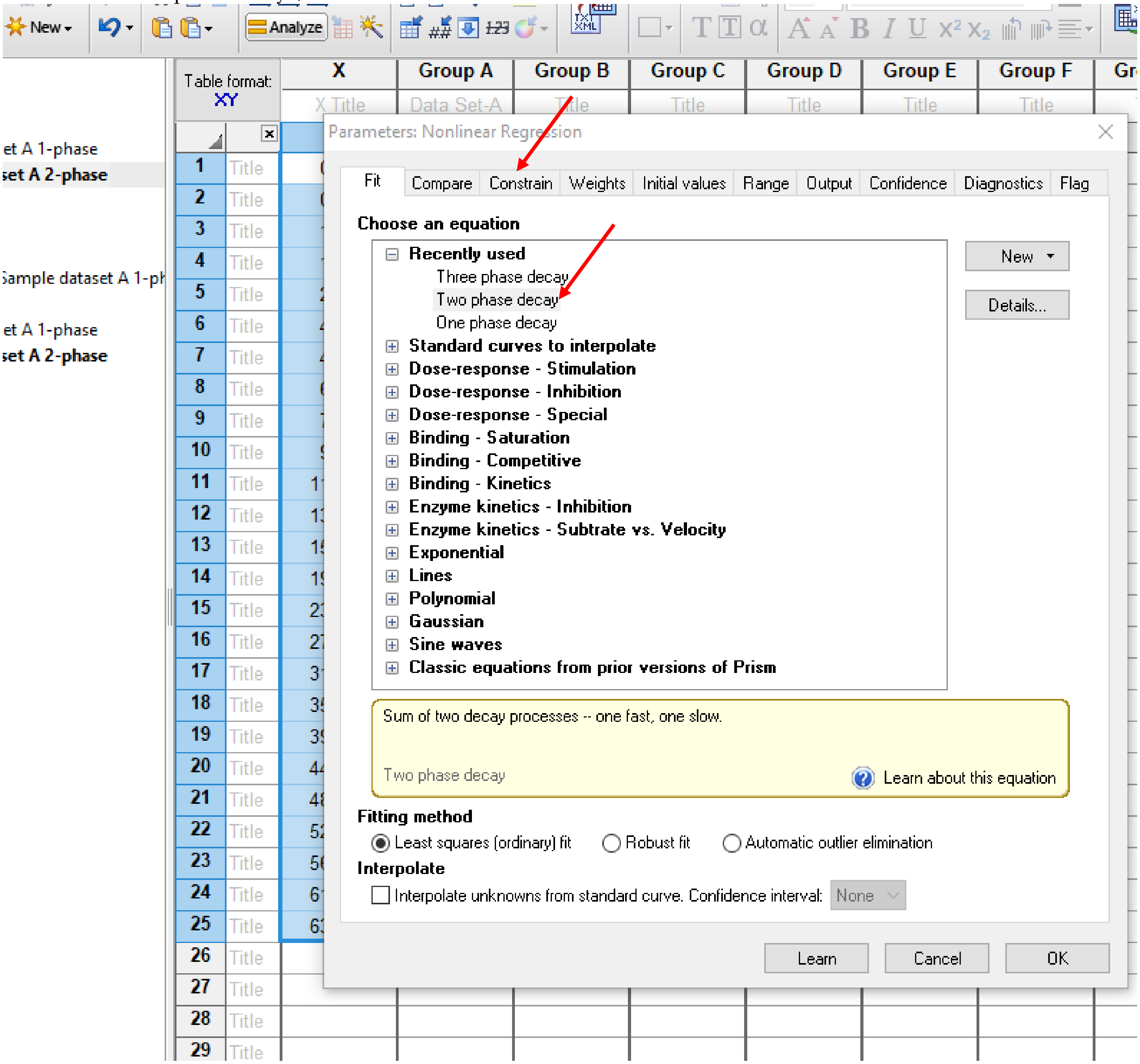

15. For Y0, change constraints from “No constraint” to “constraint equal to 100” and for Plateau, change constraints from “No constraint” to “constraint equal to 0”, then click on “OK”.

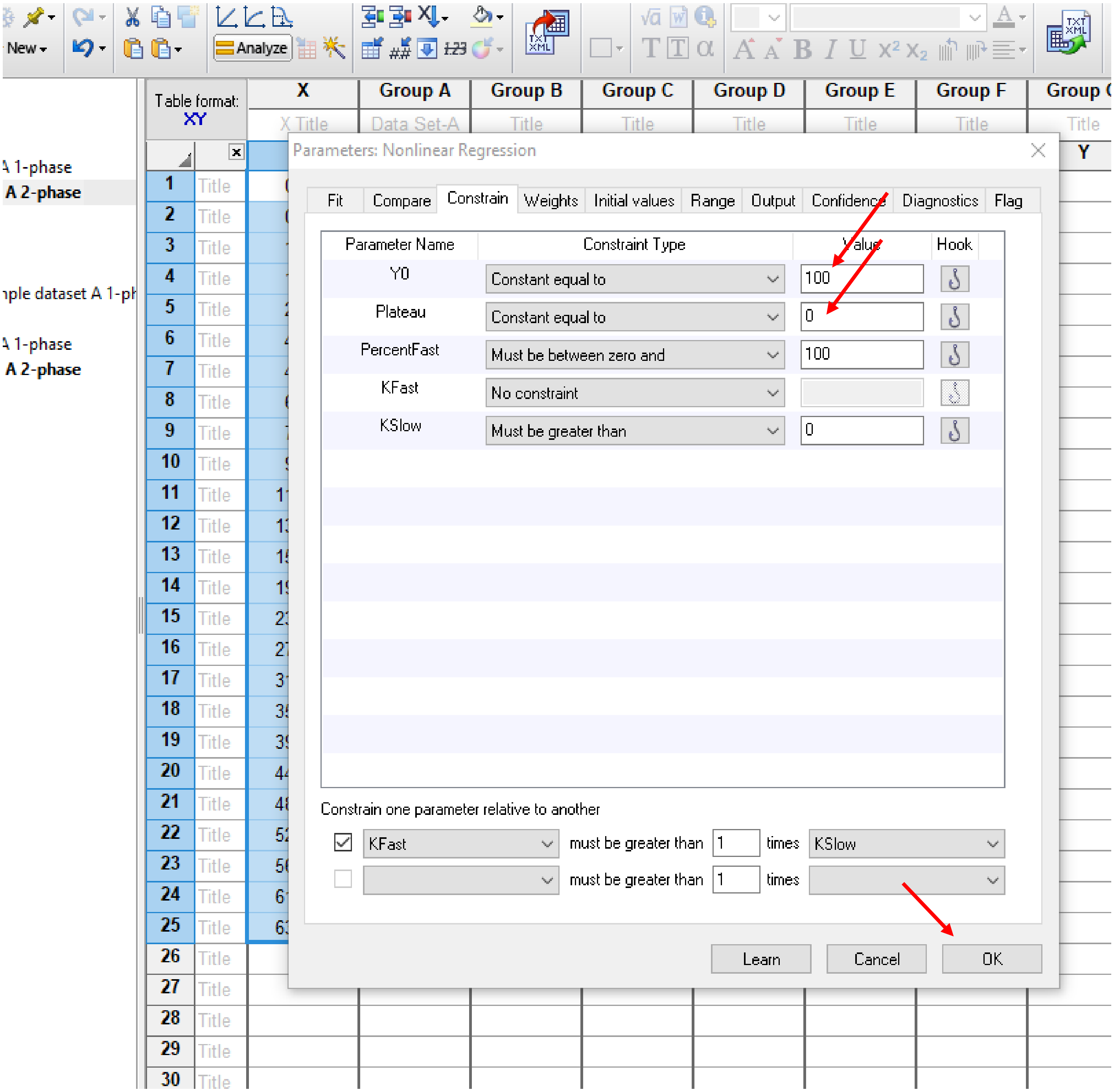

16. The 2-phase decay exponential decay nonlinear regression analysis fit our definition for a curve fitting a 2-phase decay model. We have defined curves as fitting 2-phase decay models if the "Percent Fast" is ≥ 1% and ≤ 99% (meaning that each of the "fast" and "slow" subpopulations will constitute at least 1% of the entire population. Note that most analyses will provide a value for "Percent Fast", but we do not define it as fitting a 2-phase decay model if this value is <1% or >99%. We also have required that the "Half Life (Slow)" be ≥ twice as long as the "Half Life (Fast)."

For this illustrative analysis, the proportion of the patients in the rapidly progressing group was 85.79%, with 95% confidence intervals of 78.2 to 87.14%. The PFS half-life for the rapidly progressing group was 8.4 months (7.5 to ? months). The PFS half-life for the slowly progressing group appears to be very long, but 95% confidence intervals cannot be defined since follow up is much too short. As in most of these analyses in patients with a potentially cured subpopulation, we can conclude that the PFS half-life for the potentially cured group overestimates the true PFS half-life for the group since the half-life is longer than human life expectancy.

set A 1-phase
set A 2-phase

1

Sample dataset A 1-ph
Sample dataset A 2-

set A 1-phase
set A 2-phase

| | Nonlin fit | A | B |
|---|---|---|---|
| | | Data Set-A | Title |
| | | Y | Y |
| 1 | Two phase decay | Hit constraint | |
| 2 | Best-fit values | | |
| 3 | Y0 | = 100 | |
| 4 | Plateau | = 0 | |
| 5 | PercentFast | 85.79 | |
| 6 | KFast | 0.08261 | |
| 7 | KSlow | ~ 2.009e-016 | |
| 8 | Half Life (Slow) | ~ 3.45e+015 | |
| 9 | Half Life (Fast) | 8.391 | |
| 10 | Tau (slow) | ~ 4.978e+015 | |
| 11 | Tau (fast) | 12.11 | |
| 12 | Rate constant ratio | ~ 4.112e+014 | |
| 13 | Std. Error | | |
| 14 | PercentFast | 4.03 | |
| 15 | KFast | 0.004935 | |
| 16 | KSlow | | |
| 17 | Rate constant ratio | | |
| 18 | 95% CI (profile likelihood) | | |
| 19 | PercentFast | 78.2 to 87.14 | |
| 20 | KFast | ??? to 0.09208 | |
| 21 | KSlow | | |
| 22 | Half Life (Slow) | | |
| 23 | Half Life (Fast) | 7.528 to ??? | |
| 24 | Tau (slow) | | |
| 25 | Tau (fast) | 10.86 to ??? | |
| 26 | Goodness of Fit | | |
| 27 | Degrees of Freedom | 22 | |
| 28 | R square | 0.9973 | |
| 29 | Absolute Sum of Squares | 60.41 | |
| 30 | Sy.x | 1.657 | |
| 31 | Constraints | | |

17. In this analysis, we also received the notification “hit constraint”. This indicates that the exponential decay nonlinear regression analysis calculations were impacted by one of our constraints (“Y=100” or “Plateau=0”). This is generally from the constraint “Plateau=0”. Removing the constraint usually eliminates the “hit constraint” notification, but it generally also results in the data no longer fitting a 2-phase decay model, even when log-linear plots display clear 2-phase decay with a deviation to the right at an inflection point. The calculation typically hits the constraint if the length of the PFS curve/ maximum patient follow-up is relatively short. When this constraint is hit, the program typically cannot calculate 95% confidence intervals for “Half-Life (Slow)”, but it generally can calculate 95% confidence intervals for “Percent Fast”. When constraints are hit, the program can generally calculate the lower boundary of 95% confidence intervals for “Half Life (Fast)” but could only calculate the upper boundary of 95% confidence intervals for “Half Life (Fast)” for 27% of curves hitting constraints in the analyses in this manuscript.  The bottom line: as with calculations of medians or hazard ratios or assessment of any other clinical trial data, confidence in population survival kinetics estimates improves with longer follow up and more mature data.

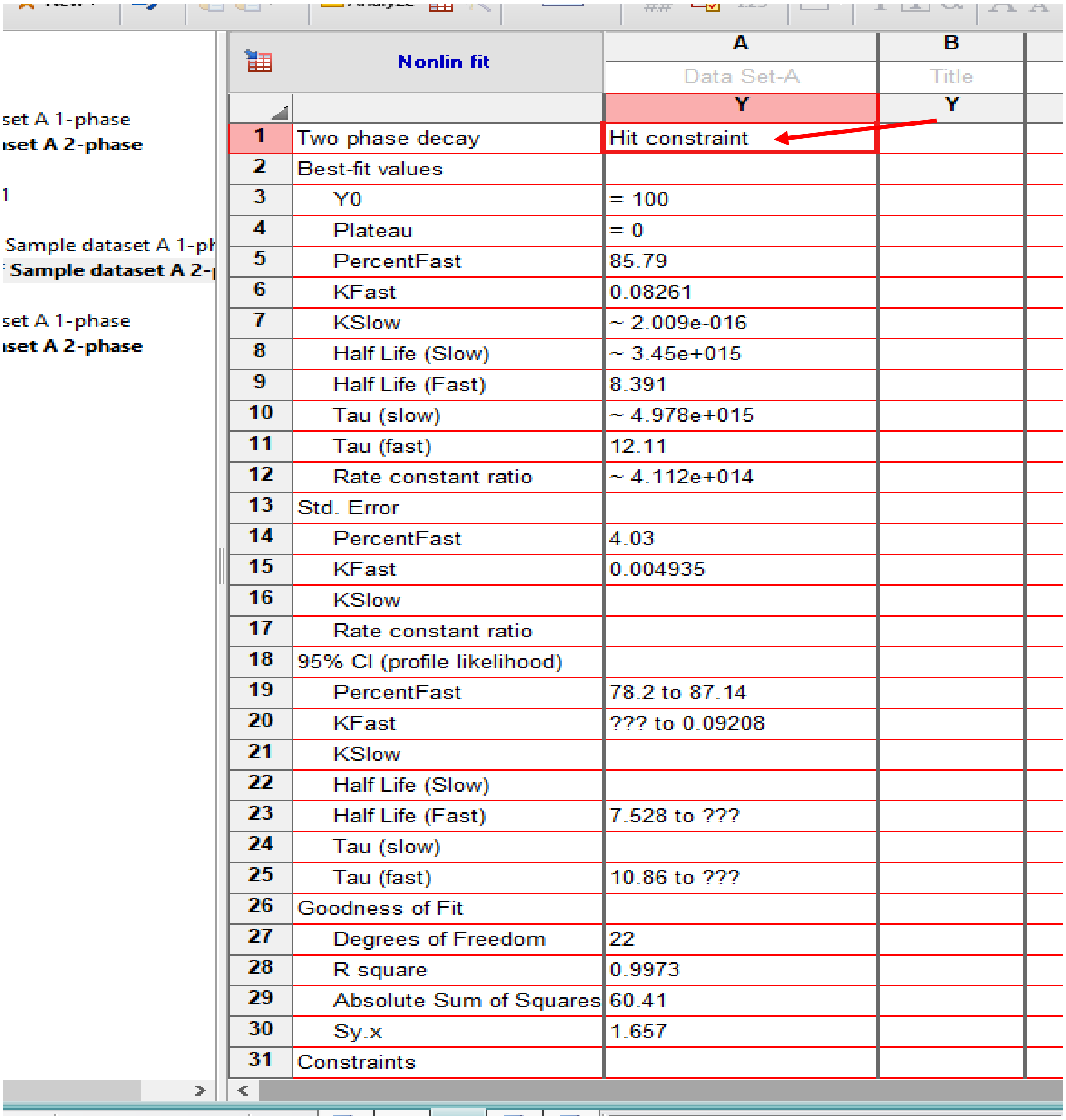


| | Nonlin fit | A | B |
|---|---|---|---|
| | | Data Set-A | Title |
| | | Y | Y |
| 1 | Two phase decay | Hit constraint | |
| 2 | Best-fit values | | |
| 3 | Y0 | = 100 | |
| 4 | Plateau | = 0 | |
| 5 | PercentFast | 85.79 | |
| 6 | KFast | 0.08261 | |
| 7 | KSlow | ~ 2.009e-016 | |
| 8 | Half Life (Slow) | ~ 3.45e+015 | |
| 9 | Half Life (Fast) | 8.391 | |
| 10 | Tau (slow) | ~ 4.978e+015 | |
| 11 | Tau (fast) | 12.11 | |
| 12 | Rate constant ratio | ~ 4.112e+014 | |
| 13 | Std. Error | | |
| 14 | PercentFast | 4.03 | |
| 15 | KFast | 0.004935 | |
| 16 | KSlow | | |
| 17 | Rate constant ratio | | |
| 18 | 95% CI (profile likelihood) | | |
| 19 | PercentFast | 78.2 to 87.14 | |
| 20 | KFast | ??? to 0.09208 | |
| 21 | KSlow | | |
| 22 | Half Life (Slow) | | |
| 23 | Half Life (Fast) | 7.528 to ??? | |
| 24 | Tau (slow) | | |
| 25 | Tau (fast) | 10.86 to ??? | |
| 26 | Goodness of Fit | | |
| 27 | Degrees of Freedom | 22 | |
| 28 | R square | 0.9973 | |
| 29 | Absolute Sum of Squares | 60.41 | |
| 30 | Sy.x | 1.657 | |
| 31 | Constraints | | |
